# Cell Fate Decision as High-Dimensional Critical State Transition

**DOI:** 10.1371/journal.pbio.2000640

**Published:** 2016-12-27

**Authors:** Mitra Mojtahedi, Alexander Skupin, Joseph Zhou, Ivan G. Castaño, Rebecca Y. Y. Leong-Quong, Hannah Chang, Kalliopi Trachana, Alessandro Giuliani, Sui Huang

**Affiliations:** 1 Department of Biological Sciences, University of Calgary, Calgary, Alberta, Canada; 2 Institute for Systems Biology, Seattle, Washington, United States of America; 3 Luxembourg Centre for Systems Biomedicine, Esch-sur Alzette, Luxembourg; 4 Corporación Parque Explora, Department of innovation and design, Medellin, Colombia; 5 5AM Ventures, Menlo Park, California, United States of America; 6 Environment and Health Department, Istituto Superiore di Sanità, Roma, Italy

## Abstract

Cell fate choice and commitment of multipotent progenitor cells to a differentiated lineage requires broad changes of their gene expression profile. But how progenitor cells overcome the stability of their gene expression configuration (attractor) to exit the attractor in one direction remains elusive. Here we show that commitment of blood progenitor cells to the erythroid or myeloid lineage is preceded by the destabilization of their high-dimensional attractor state, such that differentiating cells undergo a critical state transition. Single-cell resolution analysis of gene expression in populations of differentiating cells affords a new quantitative index for predicting critical transitions in a high-dimensional state space based on decrease of correlation between cells and concomitant increase of correlation between genes as cells approach a tipping point. The detection of “rebellious cells” that enter the fate opposite to the one intended corroborates the model of preceding destabilization of a progenitor attractor. Thus, early warning signals associated with critical transitions can be detected in statistical ensembles of high-dimensional systems, offering a formal theory-based approach for analyzing single-cell molecular profiles that goes beyond current computational pattern recognition, does not require knowledge of specific pathways, and could be used to predict impending major shifts in development and disease.

## Author Summary

A certain type of multipotent progenitor cell of the blood can commit to either the white (myeloid) or the red (erythroid) blood cell lineage, thus making a discrete binary cell fate decision. To test a theory on fundamental principles of cell fate dynamics (as opposed to the usually studied molecular mechanisms), we monitored such a fate decision in vitro using single-cell resolution gene expression analysis. We found that blood progenitor cells undergoing a fate decision to commit to either lineage after treatment with fate-determining cytokines, according to theory, first destabilized their original state. Cell states hereby diversified, manifesting the predicted flattening of an attractor’s potential well, which allows the increasingly vacillating progenitor cells to “spill” into adjacent potential wells corresponding to either lineage—myeloid or erythroid. This destabilization of an old stable state until suddenly opening access to new stable states is consistent with a critical transition (tipping point). We propose and demonstrate a new type of early warning signal that precedes critical transitions: an index I_C_ based on a change in the high-dimensional cell population structure obtained from single-cell resolution measurements. This index may be used to predict imminent tipping point–like transitions in multicell systems, e.g., before pathological changes in tissues.

## Introduction

A multipotent stem cell or a progenitor cell is in a state that poises it to be able to commit to one of multiple available options of predestined cell lineages and to differentiate. However, its state-characteristic gene expression profile is stably maintained because the cell is in a stable (or meta-stable) attractor (potential well) [1,2] generated by the gene regulatory network (GRN) in the high-dimensional gene expression state space. An attractor state represents a local minimum or, as sometimes referred to, a ground state [3], the lowest point in the basin of attraction of the attractor. The high-dimensional attractor guarantees that the state-characteristic genome-wide gene expression pattern is self-stabilizing, withstanding the stochastic molecular fluctuations. Therefore, as cells differentiate and alter their gene expression pattern in a coordinated manner to ultimately implement the expression pattern of the new cell type, they must first overcome this stabilization of the progenitor ground state imposed by the GRN.

Individual multipotent progenitor cells can, due to the stochastic gene expression fluctuations, temporarily and by chance, approach the border of the basin of attraction of their attractor and thereby be transiently primed to exit the progenitor state in a random direction, giving rise to the occasionally observed spontaneous, apparently stochastic differentiation into one of a set of alternative lineages. It is thought that the associated (unlikely) chance configurations of expression in the appropriate set of regulatory genes could place an outlier cell so as to facilitate its jump over basin boundaries into the neighboring basin of attraction of a destination lineage [4–6]. Once in the new basin, the cell will robustly establish the new gene expression pattern of the respective destination cell type as it enters the new attractor state [2].

In the qualitative parlance invoking gene regulatory circuitries, a committed cell’s robust, self-sustained, and apparently irreversible move toward a new (differentiated) state once it has left the old (undifferentiated) state is often explained by the reinforcing activity of a positive feedback control loop. By contrast, the formal approach that treats complex regulatory networks in an integrative manner as a high-dimensional dynamical system posits that stable cell states (cell types) are attractor states. This formalism naturally explains, without invoking specific gene regulatory circuitries, why spontaneous differentiation without instructive signal can produce the highly specific gene expression patterns of existing cell types [1, 2].

**Fig 1:**
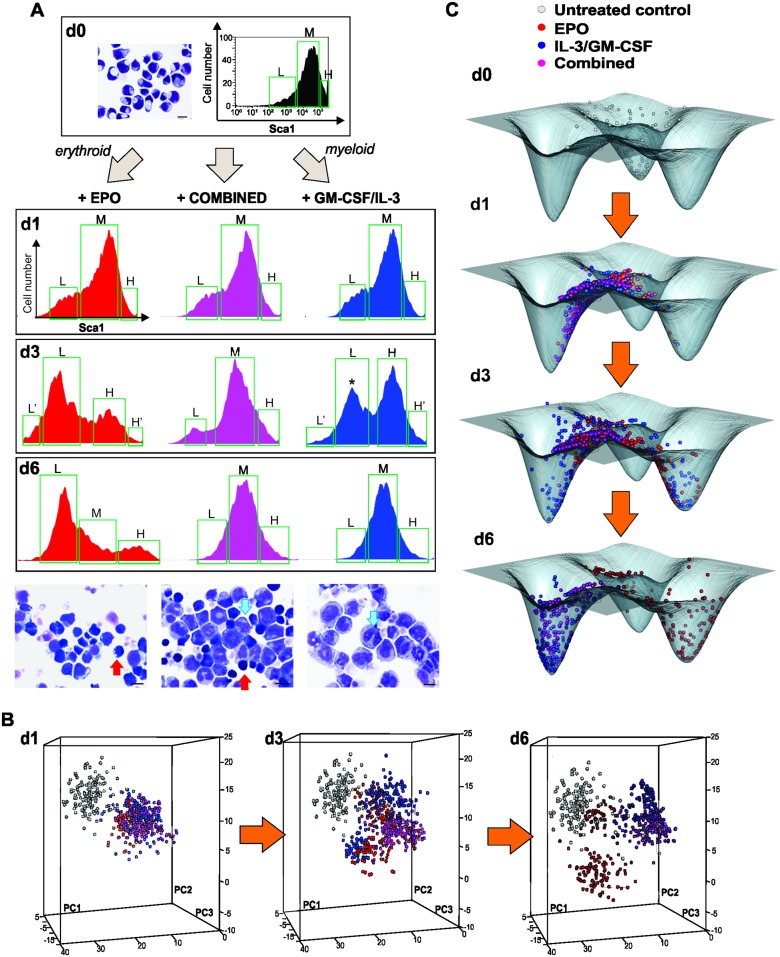
Single-cell analysis of transcript expression during binary fate decision in EML cells. **(A)** A progenitor EML cell population was stimulated with the cytokines erythropoietin (EPO) (left), interleukin-3/granulocyte macrophage-colony stimulating factor (IL-3/GM-CSF) (right), or with a combination of EPO and GM-CSF/IL-3 (center). Flow cytometry histograms of Sca1 surface expression were gated into Sca1^LOW^ (L), Sca1^MEDIUM^ (M), and Sca1^HIGH^ (H) fractions or subpopulations (green boxes) during fluorescence-activated cell sorting (FACS) of single cells at the indicated days for use in later analysis (Fig 2). At d3, further division to account for the extreme outliers (Lʹ, Hʹ)* indicates “rebellious cells” (see text). As previously reported [4], myeloid differentiation in EML cells is driven by the Sca1^HIGH^ fraction of cells and the global decrease of Sca1 expression is delayed, and can bounce back to intermediate state once cell have passed the bimodal (d3) stage. **(B)** For visualization of individual cells’ transcript expression patterns (of *m* = 17 genes), cells were projected onto a dimension-reduced state space spanned by the first three principal components (PC) following principal component analysis (PCA, see S1 Appendix). Each sphere represents a cell, colored according to treatment: untreated progenitors (grey); cells treated with EPO (red), cells treated with GM-CSF/IL-3 (blue); and combined-treated cells (purple). **(C)** To calculate a quasi-potential landscape for the three cell types for visualization of the idea of attractors as potential wells, a Gaussian filter with σ = 2 was applied to PC1 and PC2 coordinates of cells at d0 and d6 treated with EPO and GM-CSF/IL-3, leading to a smooth 2-dimensional distribution p. With the (quasi-)steady state assumption, the attractor landscape was visualized relative to a base level of 0 by − log(*p* +1). This time-invariant schematic is only a visual guide and does not model the landscape distortion during the bifurcation. Numerical data for the PCA graphs can be found in the supporting file S2 Data.

Under physiological conditions, spontaneous differentiation is rare. But appropriate tissue signals can trigger an efficient exit from the progenitor attractor and the commitment to a specific cell lineage. Here, the fundamental question remains as to how such signals overcome the stability of the progenitor attractor state and direct the fate decision toward another attractor representing a particular cell type. One possibility is that differentiation signals operate simply by coordinating gene expression changes in a deterministic manner so as to place the cells either at a specific site on the border of the basin of attraction of the progenitor cell, thus priming the cell to be susceptible to noise-driven entry into the desired destination attractor of the new lineage, or already at a state within the basin of attraction of that destination cell type. This older view [7], schematically intuited in Fig 1 (right panels), does not involve any distortion of the attractor basins. The alternative possibility is that the differentiation signal may cause a destabilization of the (high-dimensional) gene expression attractor state, thereby drastically facilitating the (noise-driven) exit from the progenitor attractor and entry into a new attractor state. The destabilization can be imagined as a flattening of the potential well. Then, a bias (tilt) in the direction of destabilization (asymmetric flattening of the attractor well) would allow the differentiation signal to influence the fate decision toward a given lineage [8].

A destabilization that results in the disappearance of an attractor state constitutes a bifurcation event in a nonlinear dynamical system: a “sudden” qualitative shift of a system’s configuration of steady states *x*(*t*) (here, from existence to nonexistence of the progenitor attractor) while a control parameter, the bifurcation parameter *μ* in the systems equations that describe the dynamics of the system, *ẋ*(*t*) = *F*(*x*(*t*), *μ*), is continuously altered [9–10]. Here, however, the system equations of the high-dimensional system (see S1 Fig), notably, the bifurcation parameters *μ*, are typically not known. In such cases, a *phenomenological* description of the bifurcation behavior is more appropriate, in which the bifurcation appears as a *critical state transition* [11–12]. Herein, a presumed stable attractor state is observed to gradually destabilize until the system (cell) suddenly passes a tipping point. At this critical point, the attractor basin is completely flattened (at least with respect to one state space dimension), and a new neighboring attractor state that is discretely distinct from the initial one becomes accessible. The system (the cell) can then rapidly descend into it.

Critical state transitions have been implicated in abrupt shifts in ecosystems, climates, and social systems, and also in disease transitions [13]. The preceding destabilization of the system is equivalent to a weakening of the stabilizing forces. Hence, it is manifest in increased (noise-driven) excursions of the system state *x*(*t*) away from its stable steady-state (equilibrium) point x*, as well as in a slowed return to it. These manifestations are plausible within the permissive image of a flattening attractor basin. Such observables of a system approaching a bifurcation event are the early warning signals of an impending critical transition and can often be quantified as an increase in the amplitude and temporal autocorrelation of the stochastic fluctuations of the systems variable *x*(*t*) around x* [11–12].

While critical transitions have been widely studied in systems whose system state can be described by a one-dimensional state variable *x*(*t*) [11–13], cell states are defined by a high-dimensional state vector **x**(*t*) [14], which, for practical purposes, can be defined by the reliably measurable transcript abundance of a set of *m* genes that participate in the shift of gene expression patterns associated with the cell state transition. Systems equations that describe the change in time of a GRN state **x**(*t*) could, in principle, be formulated to model all the regulatory interactions of the relevant regulatory genes and predict the existence of a bifurcation in the dynamics of **x**(*t*). However, because the specification of the regulatory interactions required for such dynamical models are typically not available in sufficient detail despite our increasing knowledge of the topology of GRNs (S1 Fig), the identity of the relevant bifurcation parameter whose gradual change would drive the differentiation process remains elusive. Nevertheless, numerous generic mathematical models of the dynamics of small GRNs driving cell differentiation for specific cell types have been proposed and successfully predict attractor states and bifurcations that map to the observed cell state behaviors [8,15–20].

Given the uncertainties in GRN specification for cell fate decisions, we depart from such explicit modeling of GRN dynamics and study them within the phenomenological framework of critical state transitions. This is warranted because cell lineage commitment by a multipotent cell is, in fact, characterized by a sudden, discontinuous shift of a stable cell state to another state. But instead of monitoring a single state variable *x*(*t*) continuously, we measure a set of *m* = 17 transcripts that serve as components of the high-dimensional state vector **x**(*t*) and are members of a core regulatory network that is involved in the commitment of a multipotent progenitor cell to either the myeloid or erythroid lineage (S1 Fig). Because high-dimensional gene expression profiles can currently only be measured in a destructive manner, their changes cannot be monitored in a continuous way but only at discrete time points in replicate systems. High-dimensional critical state transitions have been studied for global shifts in microarray-based transcriptomes [21], but this type of data on **x**(*t*) is an aggregate of heterogeneous mixtures of dynamical systems (i.e., cells).

Here we take advantage of single-cell resolution measurements of the state vector **x**(*t*) representing the GRN state of an actual system (a cell), defined by its abundance of the *m* species of transcripts but for a population of cells that represents a statistical ensemble of *n* systems (cells). We derive a generic quantity, the index *I*_*C*_(*t*), that is computed from the (*n* × *m*) data matrix **X**(*t*) at discrete time points *t* during the differentiation process. Thus, in essence, we make up for the lack of a continuous time series that captures the fluctuations of cell state by exploiting the availability of the individual states in a statistical ensemble of *n* cells, measured as time snapshots. We show formally and experimentally that a (relative) increase of *I*_*C*_(*t*) serves as an early warning signal of a critical transition that coincides with lineage commitment following a gradual destabilization of the multipotent progenitor state. Thus, we exploit high-dimensionality and the new granularity afforded by single-cell gene expression analysis of a cell population and take into consideration first principles from the theory of nonlinear dynamical systems to predict, without explicit modeling of the underlying regulatory interactions, an impending qualitative phenotype shift. Our theory also explains the observation of “rebellious cells” during binary fate decisions and, together with the findings, unites the old dichotomy between selection and instruction in cell fate determination.

## Results

### Single-Cell Resolution Gene Expression Analysis Suggests Preexisting Stable Attractor States

To determine if differentiation goes through a tipping point in high-dimensional gene expression state space, we studied the commitment of the murine multipotent hematopoietic precursor cell line EML [22] into an erythroid or a myeloid fate when released from the progenitor state and stimulated either with EPO (erythropoietin) or with GM-CSF (granulocyte macrophage colony-stimulating factor) / IL-3 (interleukin 3), respectively [4]. In a third experiment, we treated EML cells with a combination of EPO and GM-CSF/IL-3 to separate destabilization from fate choice, because we reasoned that the latter should be neutralized by the conflicting combination treatment. To ensure that heterogeneity of the starting cell population is strictly due to dynamic fluctuations and not due to preexisting, differentially preprimed cells of unknown developmental history (which then would merely be selectively enriched for particular fates by the respective growth factors) [23], we used a clonal cell line as opposed to purified primary cells. This permitted the study of the actual phenotypic diversification in maximally uniform cell populations of cells recently derived from a single common ancestor under invariant and homogenous conditions to assure common history and maximal phenotypic homogeneity. While such a progenitor cell line may not reflect biological reality because the cells are likely trapped in a ground state attractor not necessarily present in vivo [3, 24], as readily revealed by molecular profiling, it offers a robust model system to expose and study fundamental principles of dynamical systems that do not depend on idiosyncrasies of the specific biology.

We monitored transcript expression patterns at single-cell resolution using qPCR to acquire information about the stability of a nominal cell state **x**(*t*) presented by the cell population, which constitutes a statistical ensemble of (randomly distinct) replicates of a system. For instance, increase in cell state diversity would suggest destabilization of the nominal cell state. We found that qPCR was far more sensitive than single-cell RNA-seq in the detection of low abundance transcripts (S1 Appendix, A.7). The problem of low sensitivity is amplified by the low capture rate of transcripts in single cell–gene expression analysis (typically 60% to 90% of transcripts of a given cell are lost) and by technical (sampling) noise [25–27]. This can lead to false-positive sets of mutually exclusive expression of transcripts and thereby inflate cell-cell diversity, which is a crucial quantity in our analysis (S1 Appendix, A.8) [28].

Exit from the progenitor state was first verified by flow cytometry measurement of the downregulation of the stem-cell markers Sca1 and c-kit. The induction of a bimodal distribution with a new discrete subpopulation with lower Sca1 (and c-kit) surface protein expression confirmed the switch-like state transition to a committed state (Fig 1A). Fig 1B shows the time course of single-cell transcript patterns of 17 selected genes known to be functionally involved in or to mark the fate commitment of EML cells, plus two “housekeeping genes” (S1 Fig and S1 Table).

The single-cell states were visualized by plotting each cell as a point in the Cartesian space spanned by the three principal components (PC) from a principal component analysis (PCA) of concatenated expression data across all time points to reduce the 19-dimensional state space (17 genes of interest + 2 reference genes; see S1 Appendix). As seen in Fig 1B, the “cloud” of untreated cells (grey, depicted for reference for each time point) spread upon treatment (colored balls; where red and blue colors are a priori labels, indicating treatment with EPO or GM-CSF/IL-3, respectively), and reached highest diversity at day 3 (d3). The cells then coalesced into two distinct dense clusters at day 6 (d6), representing the cells committed to the erythroid (red) and myeloid (blue) lineages, which were identified by the characteristic expression of erythroid or myeloid transcript levels (S2 Fig and S1 Table). As shown in S3 Fig, in this single-cell qPCR, measurement noise was only a small fraction of biological cell-to-cell variability; thus, the dispersion of points in state space predominantly reflects biological diversity of cells. Loading of gene scores show that PC1 captures the erythroid–myeloid dichotomy, whereas PC2 reflects the stemness–differentiation axis (S4 Fig). For visualization purposes, single-cell resolution measurement, which provides the local cell density for each position in state space, can be depicted as the elevation of an approximate (fixed) quasipotential landscape (Fig 1C, legend) [29], which serves as visual guide and shows the three attractor states as minima (potential wells) compressed into one landscape.

Interestingly, progenitor cells receiving a combined treatment also diverged at d3 but stayed in an intermediate, undecided region of the state space before consistently joining the myeloid cluster (Fig 1B). Thus, the conflict of signals delayed the fate decision, but a uniform decision was eventually made. This decision-making, in view of ambiguous signals, corroborates the notion that gene expression change during lineage determination is not simply instructed by external growth factors but also governed by intrinsic constraints that channel cells toward predestined fates—the attractors of the GRN. Importantly, this model does not allow for stable intermediates, as Waddington first observed [30]. In this case, it appears that the attractor for the myeloid fate is more readily accessible or that, in our combined differentiation protocol, the myeloid signal somehow dominates, although the cells that resolved the conflict still remained distinguishable from the pure myeloid cells.

### Destabilization of the Progenitor State and the Critical State Transition Index I_C_

Independent of the (unknown) detailed dynamics of the underlying GRN, a destabilization and disappearance even of a high-dimensional attractor state is a bifurcation event and, therefore, should display the signatures of an approach to a critical state transition [11] at which cells would undergo a discontinuous switch toward the destination state. While the bimodal distribution of Sca1 (Fig 1A) after d3 indeed suggests a quasi-discrete transition, it cannot reveal a destabilization of a high-dimensional state **x**(*t*) prior to the switch. Recently reported cases of critical transitions in stressed ecosystems and disease processes (refs. in [13]) pertain to low-dimensional systems in which, typically, one system variable *x*(*t*) was observed longitudinally over time. By contrast, here we examine time snapshots of states of a high-dimensional system (*m* = 17-dimensional cell state vector) embodied by the GRN.

Based on theoretical consideration, we showed that a critical destabilization and transition to a new attractor will be manifest in two changes in the correlation statistics (as explained and derived in S2 Appendix, B.1–B.3) of the (*n* × *m*) data matrix **X**(t) [31]:

The first change is (trivially) a decrease of cell–cell correlation *R*(cell *k*, cell *l*) between all pairs of the *n* cell state vectors in the *m* = 17-dimensional gene space. This reflects the expected increase of amplitudes of random fluctuation of gene expression due to the weakening attracting force in the flattening basin of attraction prior to the bifurcation [32]. This decrease in the coefficients of correlation *R* between all pairs of *n* cell state vectors captures an increase in cell–cell diversity.

The second change is the concomitant increase of gene–gene correlation *R*(gene *i*, gene *j*) between all pairs of the *m* gene vectors that describe the gene expression values of each gene across all the cells. Unlike the former, this change is less intuitive. The increase in the correlation coefficients between all pairs of the *m* gene vectors (with *n* components each), as mathematically derived in S2 Appendix, arises because of the symmetry-breaking destabilization in a high-dimensional attractor. In short, this can be made plausible from two different perspectives:

1. In statistical terms, this change is a consequence of the range restriction effect on correlation, when the dominance of the symmetric stochastic fluctuations of gene expression around the attractor state, which minimizes gene–gene correlations, yields to the nonsymmetric, regulated change of gene expression (S2 Appendix, B.2) [31,33].
2. In the dynamical system description, this change is a formal consequence of the appearance of a saddle point in the *m*-dimensional state space through which the individual cells pass during the bifurcation, and in doing so align along a reaction coordinate, thus occupying a subspace of reduced dimensionality (see S2 Appendix, B.3).

Note that here, “correlation” (between cells or genes) does not simply have the usual function of indicating an association between two variables but is derived from elementary principles of the constraints in data and of dynamical systems theory. The above considerations concerning the two opposite changes in the two correlation statistics then motivate an index for critical transitions, *I*_*C*_:

$$I_{C}\left(t\right)=\;\frac{\langle \left|R\left(g_{i},\;g_{j}\right)\right|\rangle }{\langle R\left(S^{k},\;S^{l}\right)\rangle },$$

where ***g*** are gene vectors, ***S*** are the cell state vectors at sampling time *t*, and 〈*R*(…,…)〉 denotes the average of all Pearson’s correlation coefficients of respective pairs of vectors. We postulate that *I*_*C*_ increases toward a maximum when cells go through the critical state transition (S2 Appendix). Recently, Chen et al. proposed a similar index for full transcriptome time courses, which, for lack of single-cell resolution state vectors, has to estimate state diversification and requires a separate explicit selection of a subset of genes among which the correlation is computed [21]. Here we start from a predefined set of *m* = 17 selected genes that are known to change significantly (as actuator or as marker) during fate commitment in order to demonstrate the signature of a high-dimensional critical state transition.

**Fig 2:**
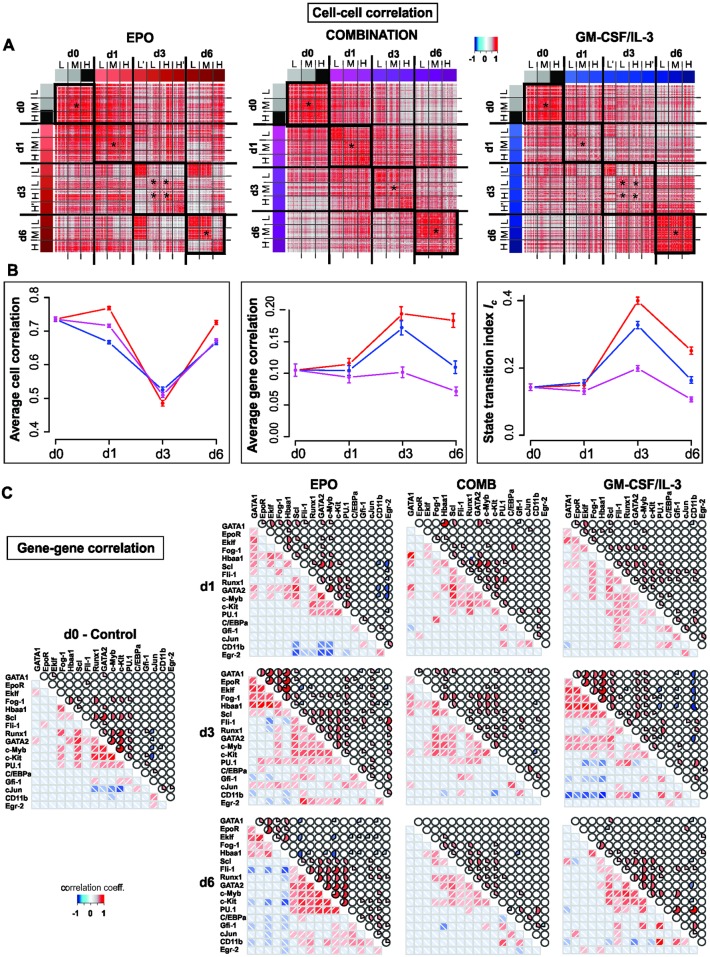
Critical transition during lineage commitment. **(A)** Cell–cell correlation matrices displaying the Pearson correlation coefficient *R*(*S*^*k*^, *S*^*l*^) for all pairs of cells in states *S*^*k*^ and *S*^*l*^ (see S2 Appendix). *R* calculated for a set of 150 progenitor cells, 500 EPO-treated, 500 GM-CSF/IL-3-treated, and 450 combination-treated (COMB) cells from data used in Fig 1. Black squares (diagonal) emphasize the higher correlation between cells within the nominally same population. Two control genes (GAPDH and TBP) were excluded from this analysis. Lʹ, L, M, H, Hʹ indicate the Sca1 fractions shown in Fig 1: extremely low, low, medium, high, and extremely high level of Sca1 expression, respectively. **(B)** Average Pearson correlation coefficients for cell–cell pairs (left) and gene–gene pairs (center) as well as the state transition index *I*_*c*_ = 〈|*R*(*g*_*i*_, *g*_*j*_)|〉 / 〈*R*(*S*^*k*^, *S*^*l*^)〉 at various time points. Correlation coefficients were calculated for the central fractions/subpopulations in panel A(*). Error bars indicate SEM. **(C)** Gene–gene correlation matrices for the 17 genes of interest and the two endogenous control genes for the three treatments at various time points where correlation is indicated either by color (lower matrix triangle) or solid color segment in pie chart. Color values for magnitude of correlation coefficient for both matrices (A, C) are shown in color bar. Numerical data for the graphs in (B) and (C) can be found in S2 Data.

Fig 2A shows the *n* × *n* heat map for cell–cell correlation coefficients *R*(***S***^*k*^, ***S***^*l*^) for all pairs of the *n* = 1,600 cells for the three treatments (EPO, GM-CSF/IL-3 and combined) at each time point *t*. The diagonal shows that correlation of cells within the populations decreases at d1 and notably at d3, compared to d0, and increases again at d6, indicative of a transient diversification of cell states and a return to a more homogenous population consistent with an attractor state. Because we also recorded the cells’ position with respect to the Sca1 surface marker expression (roughly partitioning the population into three fractions, Sca1-high (*H*), Sca1-medium (*M*) and Sca1-low (*L*) [see Fig 1A]), one can see that the decrease of correlation was not due to comparing cells across subpopulations in bimodal populations (Fig 1A). The higher correlation among the cells within the extreme-low Sca1 fraction (*Lʹ*) in both EPO and GM-CSF/IL-3 treatment is consistent with advanced commitment of cells that are enriched in the Sca1-low fraction toward the erythroid fate, as previously reported [4]. By contrast, the high correlation among the *H* cells at the end of EPO treatment reflects “rebellious” cells that became myeloid under EPO treatment (see below).

The second criterion of a critical state transition, the increase in gene–gene correlation 〈*R*(*g*_*i*_, *g*_*j*_)〉, is shown in Fig 2B. Both EPO and GM-CSF/IL-3 treatment resulted in almost a doubling of 〈*R*(*g*_*i*_, *g*_*j*_)〉 at d3, which returned toward baseline at d6. The heat maps (Fig 2C) show that the increase of 〈*R*(*g*_*i*_, *g*_*j*_)〉 resulted from correlated (red) as well as anticorrelated gene pairs (blue) at d1 and, more pronounced, at d3. By contrast, genes were mostly uncorrelated in the progenitor state, consistent with the dominance of random fluctuations around the attractor state (explained in S2 Appendix).

Together, the cell–cell and gene–gene correlation computed from the single-cell gene expression level data matrix **X**(*t*) indeed gave rise to a temporal course of the index *I*_*C*_ that increased toward (and culminates around) d3 after induction of both fate commitments, as predicted by theory if the cell population approaches a critical transition. The maximum of *I*_*C*_ at d3 coincided roughly with the beginning of lineage separation in state space (Fig 1B). The decrease after d3 is plausible if one considers that cells enter a new attractor hereafter. However, this decrease is not strictly predicted by the theory because the assumption of ergodicity is not necessarily met if cells in distinct attractors are considered (S2 Appendix, B.2).

### Robustness of the Index *I*_*C*_

We next analyzed published single-cell gene expression data to further examine the robustness of index *I*_*C*_. Due to the intrinsic structure of the formula for *I*_*C*_, which is the ratio of the result of applying the same operation (averaging of all Pearson correlation coefficients) to a data matrix and to its transpose, it is expected that significant deviations of the value of *I*_*C*_ from 1 in random data is extremely improbable, as bootstrap analysis of our data confirms (*p* < 10^−10^; see S2 Appendix, B.1).

We first examined whether the increase of *I*_*C*_ precedes discontinuous cell phenotype transitions in other systems. Few studies monitor a cell developmental process at multiple time points prior to the key phenotype transition event, but one publicly available dataset [34] used single-cell RNA-seq for whole transcriptome profiling of the differentiation of bipotent lung epithelium progenitor cells into the AT1 and the AT2 subtypes during embryonic development and appeared to be suited for our purpose. The fate commitment to AT2 cells takes place between E16.5 and E18.5. Computing *I*_*C*_ for all reported transcripts at the various time points showed that *I*_*C*_ indeed increased significantly between E16.5 and E18.5, which was indeed due to concomitant decrease of cell–cell correlation and increase of gene–gene correlation (S3 Appendix, C.1).

Because, in this case, transcriptome-wide data was available, we next asked whether the number *m* of genes analyzed could affect *I*_*C*_. Note that *I*_*C*_ is derived under the assumption that the genes defining the cell state vector are members of the dynamical system (the core GRN) that drives or is affected by the critical transition at study, and that only the change of *I*_*C*_ but not its absolute value has a biological meaning. Thus, we examined a situation similar to the problem in reference [21], when the core set of genes is not known a priori. What can be expected when a transcriptome-wide gene set is considered? Because the majority of genes in the transcriptome are not members of the relevant core GRN, one possibility is that including a larger number of genes for computing *I*_*C*_ would decrease the sensitivity of *I*_*C*_. On the other hand, because the expression behavior of all genes in the transcriptome are already largely correlated overall, this could, due to increased possibility of the range restriction effect (S2 Appendix, B.2) [35], boost the changes in 〈*R*(*g*_*i*_, *g*_*j*_)〉 and increase sensitivity of *I*_*C*_ when more genes are considered. We thus compared randomly selected subsets of 2,000, 200, and 20 genes in the lung cell differentiation data for computing *I*_*C*_ and performed bootstrap analysis to determine the significance of change of *I*_*C*_. As shown in the Supporting Information (S3 Appendix, C.1), in all cases, *I*_*C*_ increased significantly toward the point of fate commitment (and also decreased afterwards as cells terminally differentiated to virtually the same extent for all three cases); however, the error was smaller when the number of genes considered was larger. Thus, the fact that, in our data from the EML cells, we see a drastic and significant increase of *I*_*C*_ by more than two-fold (Fig 2B) suggests that considering a small number of genes as we did here—entailed by the use of the more sensitive qPCR—sets the bar higher for statistical significance.

To test a case in which the range restriction effect does not hold because of large cell–cell variability (minimal baseline cell-cell correlation), we analyzed the single-cell transcriptome data of the most heterogeneous natural cell population we could find: glioblastoma cells [36]. Random sampling of increasingly larger sets of genes to compute *I*_*C*_ indeed showed that, with increasing number of genes considered, the average cell–cell correlation 〈*R*(*S*^*k*^, *S*^*l*^)〉 decreased—the opposite of the above cases. This elevated the absolute value of *I*_*C*_ as well as its statistical fluctuations. Thus, in case of low inherent cell–cell correlation, increasing the number of (randomly chosen) genes increases noise. However, even with 2,000 genes used and higher variance of *I*_*C*_, a change of *I*_*C*_ by two-fold as we observe (Fig 2B) would still have been significant at *p* < 0.01 (S3 Appendix, C.2).

In summary, *I*_*C*_ is robust to varying the number of genes *m* used for its computation and for a wide range of preexisting intrinsic correlation between the cells. But sensitivity is higher in less heterogeneous cell populations (as are cell lines) and is increased by the use of a selected set of genes known to participate in the phenotype transition.

### An Alternative Projection of Differentiation Trajectory Also Shows Evidence of Critical Transition

**Fig 3:**
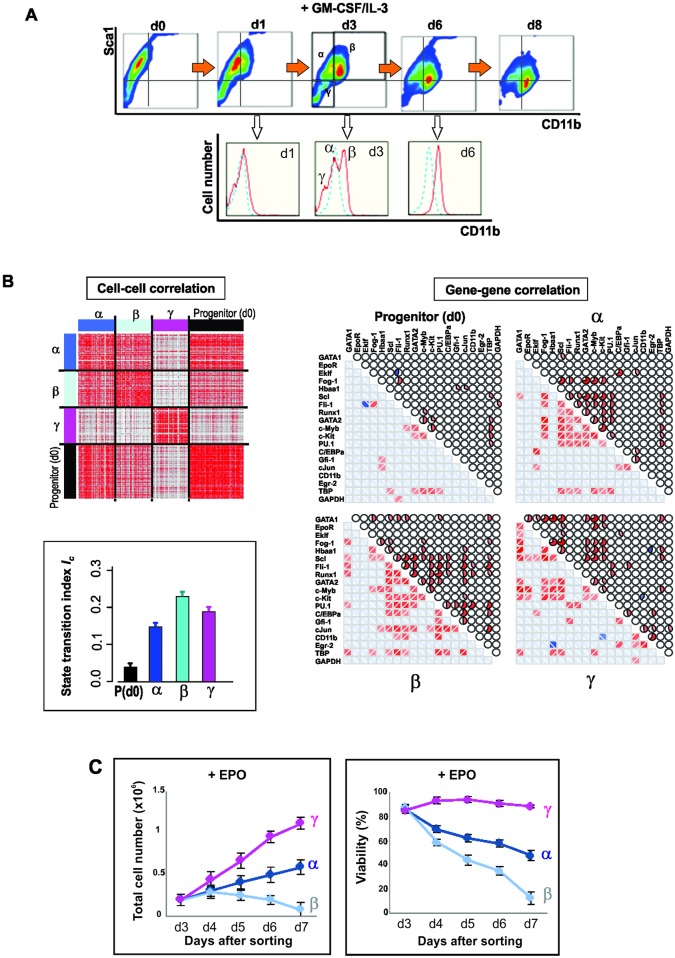
Intermediate stage of myeloid commitment along CD11b dimension exhibits destabilization of progenitor state. **(A)** Flow cytometry dot plot of expression of Sca1 and CD11b upon treatment of the progenitor EML cells with GM-CSF/IL-3. Three distinct subpopulations on d3, designated, α, β and γ, in the tri-modal distribution of CD11b flow cytometry histogram underneath (red line, treated; blue line, untreated). **(B)** Cell–cell correlation for 72 progenitor cells and 48 cells from each of the α, β, and γ subpopulations, and gene–gene correlation for all 17 genes of interest and two endogenous control genes. Pearson correlation coefficient displayed as heatmap; same color scheme as in Fig 2. Bar graphs in box show the drastic increase of *I*_*C*_ for all the three subpopulations at d3 compared to the untreated progenitor cells, P(d0). *I*_*C*_ computed as in Fig 1. **(C)** Rescue by EPO of the “rebellious” = unintended γ subpopulation (pink curve) during myeloid differentiation. Three subpopulations (α, dark blue; β, light blue; γ, pink) were FACS sorted and antibodies were removed and stimulated with EPO. Total cell number and viability were quantified on day of sorting (d3) and four subsequent days. Viability was determined based on percent of cells excluding trypan blue. Each point represents average +/- STD for two biological replicates.

To exclude the possibility that the observed pattern of gene expression changes indicating a critical transition is an idiosyncrasy linked to monitoring the exit from the progenitor attractor along the particular state space direction of decreasing Sca1 expression, we also monitored and dissected differentiation along the axis of the increase of differentiation marker CD11b, a reliable indicator of myeloid differentiation (Fig 3A). Following GM-CSF/IL-3 treatment, first CD11b surface expression increased and then Sca1 decreased; that is, the cells moved from the CD11b^LOW^/Sca1^HIGH^ to the CD11b^HIGH^/Sca1^LOW^ state. We observed that at d3, the time around which maximal destabilization was expected, the entire cell population split into three subpopulations with respect to CD11b: Sca1^HIGH^/CD11b^LOW^ (termed α), Sca1^HIGH^/CD11b^HIGH^ (β), and, unexpectedly, Sca1^LOW^/CD11b^VERY-LOW^ (γ) (Fig 3A). Single-cell transcript analysis suggests that the α-subpopulation corresponds to the destabilized but not yet fully committed cells because it displays the highest cell–cell diversity and high correlation of the gene vectors (Fig 3B, S5 Fig). The cells of subpopulation β were most advanced toward the myeloid lineage (high expression of Gfi1, CEBPα and cJun transcripts), consistent with the high CD11b expression, whereas cells of subpopulation γ correspond to rebellious cells that moved in the opposite direction from that intended by the treatment with GM-CSF/IL-3 (see below) and, thus, displayed erythroid gene expression patterns, including a large number of EpoR positive cells (S5A–S5D Fig). The index *I*_*C*_ is drastically increased in all three cell populations at d3 (Fig 3B, inset), indicating a destabilization of the progenitor attractor. Because *I*_*C*_ was computed separately for each subpopulation, this also suggests that its increase was not driven by the increase in gene–gene correlation as a trivial consequence of separation into distinct cell types.

At d6, the γ population disappears (Fig 3A), consistent with the rebellious cells in the PCA analysis of Fig 1B. However, addition of EPO to sorted subpopulations in growth factor–free cultures rescued the γ cells (Fig 3C) and, to a lesser extent, the α cells, but not the myeloid committed β cells. This finding not only confirms that the γ cells have aberrantly moved toward the erythroid lineage despite instruction for commitment to the myeloid lineage but also corroborates the notion of cell selection in fate control in which cytokines act as growth factors to determine lineage by providing the survival and mitogenic signals to the early committed cells that express the cognate receptor, in this case the EpoR [37–41].

### Critical Slowing Down of Relaxation of Outlier Subpopulations

**Fig 4:**
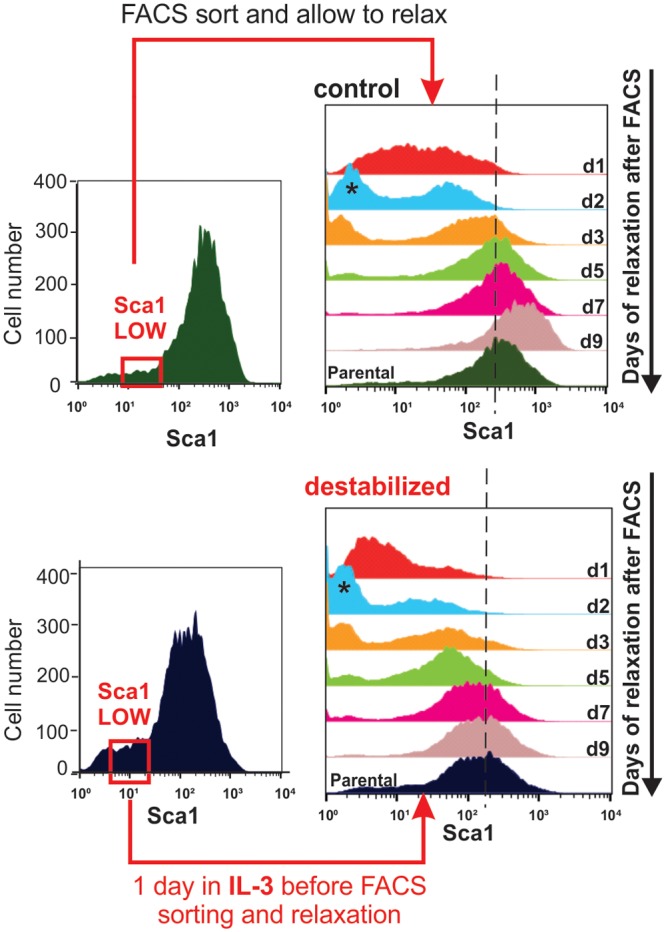
Critical Slowing down of state relaxation during fate commitment. Apparent critical slowing down of relaxation and restoring of parental distribution of the sorted Sca1-low outlier fraction in the treated population. Clonal EML progenitor cells were stimulated (top) with GM-CSF/IL-3 or not (bottom), and cells with lowest 15% Sca1 expression were FACS-sorted one day after stimulation.

We next examined a dynamical signature (early warning signal) of an approach to a critical transition used in low-dimensional systems: the slowing down of relaxation back to the original attractor states due to reduced attracting force [13, 41]. Here, critical slowing down was exposed by measuring the relaxation of sorted outlier cells, which are (transiently) in an extreme state with respect to projection into just one dimension, that of Sca1 [4]. We thus isolated the Sca1^LOW^ tail of populations either treated for 1 d with GM-CSF/IL3 to destabilize the progenitor state, or in untreated populations. As previously shown, the Sca1^LOW^ fraction re-establishes the parental distribution within 5 to 6 d [4]. By contrast, cells exposed to GM-CSF/IL-3 for just 1 d (which had not yet visibly altered Sca1 expression) required at least 9 d to reconstitute the parental Sca1 expression distribution (Fig 4). Although there could be many reasons for the impaired relaxation, including any nonspecific, nonphysiological perturbation of the progenitor state by the cytokines, these reasons may themselves be seen as a manifestation of attractor destabilization, and this finding is at least phenomenologically in line with a critical slowing down.

### Rebellious Cells as Manifestation of Noise at Bifurcation

Intriguingly, in both projections of monitoring differentiation along the axis of decreasing Sca1 (Fig 1B) as well as increasing CD11b (Fig 3A, the γ-cells), at d3 in both cases some cells consistently went in the “wrong” direction, opposite to the instruction by the respective cytokines (i.e., some EPO-treated cells were associated with the myeloid cell cluster and vice versa). Consistent with previous observations [4], the lineage of rebellious cells mirrored their Sca1 expression levels in the untreated population: EPO-treated cells moving toward the myeloid fate at d3 stemmed from the Sca1^HIGH^ fraction in the progenitor population, whereas GM-CSF/IL3-treated cells fated toward the erythroid cells originated in the Sca1^LOW^ fraction (S6 Fig). This suggests that the priming of cells in the progenitor population toward the erythroid or myeloid fate (as reflected in the Sca1 surface expression [4]), respectively, predisposes the cells to react in a rebellious way if the differentiation signal is opposed to their priming. This is consistent with an initially more or less symmetrical destabilization of the progenitor attractor state, such that wrongly primed cells are pushed toward the opposite lineage as the basin of attraction flattens and vanishes. Note that the rebellious cells disappeared at d6, possibly by transdifferentiating to the correct lineage or by dying out (if they are not rescued by providing the commensurate growth factor; Fig 3C). The existence of rebellious cells may correspond to the observation of mixed colonies in early colony assays for hematopoietic differentiation [8,42,43].

The repeated observation of rebellious cells is consistent with a bifurcation at which two new attractors become accessible, representing the dichotomy between the two sister lineages [18]. The destabilization of the progenitor attractor, unlike in the canonical saddle-node bifurcation [11], opens up a choice of two attractors, and despite a bias toward either one imposed by the lineage-determining growth factors, this still allows cells to spill into the “wrong” attractor if molecular noise overcomes the instructive bias toward the intended lineage. Thus, the existence of rebellious cells is also a signature of a critical transition.

**Fig 5:**
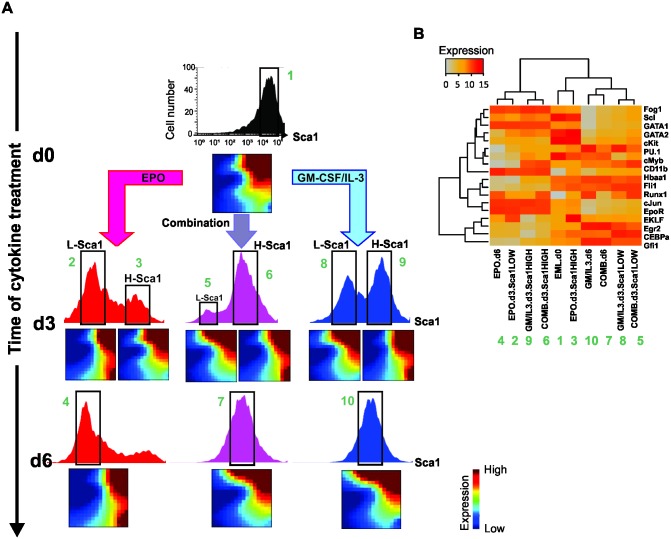
Whole-population transcriptome analysis reveals transient alternative program (rebellious cells). **(A)** Sca1 surface expression population distribution in progenitor and cytokine-treated cells and transcriptomes of sorted subpopulations at indicated treatments/time points, displayed as GEDI self-organizing maps [44]. Progenitor EML cells were stimulated with EPO alone, with GM-CSF/IL-3 alone or with the combination of the two, and the Sca1-Medium (M) fractions (d0 and d6) and/or the Sca1-Low and -High subpopulations (d3) were FACS sorted for microarray analysis. **(B)** Hierarchical cluster analysis of the microarray-based transcriptomes of samples in A (columns, correspondence indicated by the green numbers) for a subset of the 17 genes analyzed in single-cell qPCR (rows). The entire experiment was performed twice in two laboratories with similar results. Color bar represents transcript expression values in log-scale (see Materials and Methods).

To show that this polarized behavior is not an artifact of projection in one state space dimension (in this case, with respect to Sca1 or CD11b surface expression) but holds in the high-dimensional state space, we measured the transcriptomes of the subpopulations that have either responded to the growth factor or appeared to have not responded, at least with respect to change in Sca1 expression (Fig 5). As shown earlier (Fig 1), all three treatments, with either cytokines as well as combined, triggered a split of the population into two distinct subpopulations with respect to the progenitor marker Sca1 (bimodal distribution at d3, Fig 5A).

Intriguingly, cells from the Sca1^HIGH^ subpopulation that appeared to have not responded after 3 d in EPO because Sca1 stayed high (fraction #3 or H-Sca1 in Fig 5A) had a transcriptome that resembled that of the cells that had responded to GM-CSF/IL-3 treatment and had down-regulated Sca1 (fraction #8 or L-Sca1 in Fig 5A). Conversely, Sca1^HIGH^ cells that had apparently not responded yet at d3 to GM-CSF/IL-3 (fraction #9 in Fig 5A) displayed a more pronounced change of the transcriptome that was remarkably similar to that of Sca1^LOW^ cells (fraction #2) that had responded to EPO (for quantitative analysis of transcriptome similarities, see S2 Table). Extraction of those 17 genes in the microarray that were used in the single-cell qPCR analysis and hierarchical cluster analysis with these genes (Fig 5B) recapitulated these relationships, confirming that this set of genes represented the genome-wide high-dimensional dynamics well. In the combined treatment cells exhibited a transcriptome behavior similar to that of the nominally myeloid fated (i.e. GM-CSF/IL-3 treated) cells, in agreement with the single-cell transcript analysis (Fig 1).

Thus, transcriptome measurements of subpopulations that appear to have not responded to the differentiation signal with respect to downregulating the progenitor state marker suggest that they actually had responded but by altering gene expression in the non-observed state space dimensions, underscoring the importance of considering high-dimensional dynamics. The intriguing crosswise similarity of the transcriptome changes in the non-responders in one treatment to that of the responders in the other treatment strongly supports the model of a constrained dynamics with a finite number (here: two) of fate options. These are embodied by attractor states that establish the predestined developmental potentials, and they become accessible once the progenitor state is destabilized. The aberrant but highly defined behavior of rebellious cells exposes the poised instability and a stochastic, non-instructive component in fate determination.

We suspect that the rebellious cells represent those cells that, following the flattening of the progenitor attractor initiated by the external differentiation signal, erroneously enter the non-intended attractor because the stochastic gene expression fluctuations may, in some cells, overcome the instructive signal that biases the destabilization toward a specific lineage attractor. Nevertheless, the rebellious cells, being in the “wrong” fate, should eventually die because the lack of survival signals provided by the presence of the respective growth factor, as their disappearance in the measurement in Fig 1 implies. The rescue of the rebellious cells by the opposite cytokines confirms this model (Fig 3C).

Thus, instruction (extrinsic determination) and selection (driven by intrinsic stochasticity of responsiveness) synergize in fate control in a two-step scheme: cells must both be instructed and be selected for by the differentiation signal in order to adopt a particular phenotype [38–40]. This two-step process increases the fidelity of fate determination in the tissue. The attractor destabilization concept unites these two mechanisms of lineage commitment that has historically been opposed to each other but logically are not mutually exclusive [37,42,45].

## Discussion

**Fig 6:**
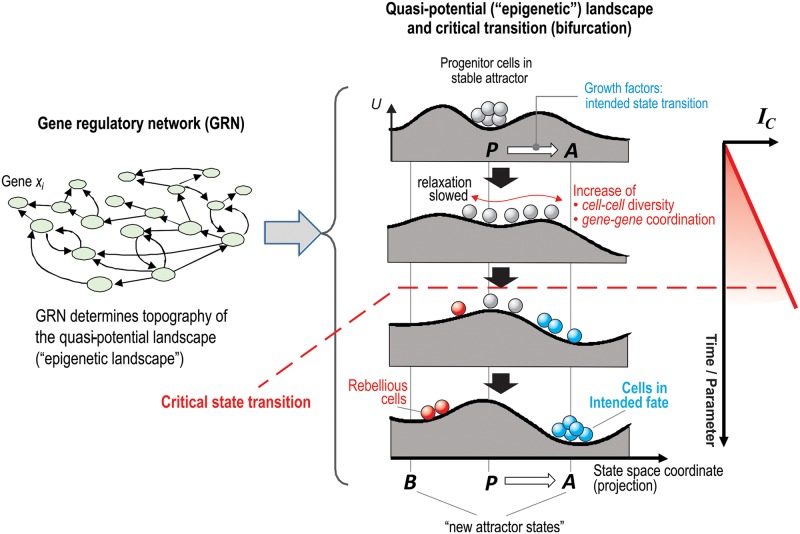
Epigenetic landscape model of symmetry-breaking bifurcation event. The architecture and the specification of the individual interactions of the gene regulatory network (left) determines the topography of the epigenetic landscape (center) in which the elevation (formally, a quasi-potential *U*) [29] visualizes the relative stability of individual cell states, represented by the position with respect to the *x*-axis (state space). Thus, valleys represent stable attractor states. Fate commitment is induced by external differentiation signals, which initiate the deformation of the landscape and can be divided into two phases: first, the destabilization of the (meta)stable attractor of the progenitor cells (grey balls) and generation of a poised unstable state; and second, the opening of access to the destination attractors for both the intended and non-intended fate, biased toward the former by the differentiation signal. This allows the cells (balls) to descend to the new attractors. The disappearance of the progenitor attractor marks the critical transition (tipping point). As explained in S2 Appendix, the increase in cell–cell variation and in gene–gene correlation as the progenitor attractor destabilizes and cells spread and align along the particular direction (reaction coordinate) to exit the old attractor gives rise to a gradual increase of the index *I*_*C*_ (right) as cells approach the critical transition. This event coincides with lineage separation in state space and a major shift in cellular transcriptome. (Note, however, that there is no proof that *I*_*C*_ reaches a peak just at the tipping point.)

Here we present a novel use of single-cell gene expression analysis that is phenomenological but informed by dynamical systems theory to make predictions (Fig 6). We show that exit from the multipotent progenitor state and commitment to a particular cell lineage exhibit signatures of a critical state transition that can be exposed by single-cell resolution gene expression analysis of a cell population undergoing cell fate commitment. This phenomenological approach does not require detailed modelling of the dynamics of the underlying gene regulatory pathways and a definition of a bifurcation parameter, which is currently not realistic given our insufficient knowledge of the GRN architecture. The key formal assumption is only that the GRN state change implementing the cell fate decision and commitment is due to a monotonical gradual alteration of the value of an (unidentified) bifurcation parameter that drives the change of the attractor landscape through a bifurcation (without specifying which type). Its chief consequence, the destabilization of a high-dimensional attractor state, can be observed. To do so, we treat the cell population as a statistical ensemble that (ergodically) explores the structure of a high-dimensional gene expression state space. The assessment of the latter directly confirmed that the notion of a critical state transition and associated early warning signals also extends to high-dimensional dynamics, as recently suggested [20,22]. Single-cell resolution analysis of a statistical ensemble at discrete time points makes up for the technical challenge of measuring the temporal fluctuations in cells, and the phenomenological but formal framework of critical transitions obviates the need for explicit modeling of the stochastic dynamics of gene regulatory circuits.

We show that high-dimensional critical dynamics of mammalian GRN can be captured by measuring the transcript levels of a set of 17 genes in individual cells at multiple time points *t* and by computing from such data the index *I*_*C*_(*t*), a quantity derived from the theory of non-linear dynamical systems that measures concomitant changes in cell–cell diversity and gene–gene coordination. Ideally, the genes considered for computing *I*_*C*_ will encompass those that undergo coordinated changes during the critical transition, as dictated by the GRN that drives the critical transition. However, we show that 20 randomly selected genes from the transcriptome may also suffice, likely because of the basal genome-wide coordination of gene expression.

*I*_*C*_ is particularly useful for single-cell resolution snapshots of molecular profiles provided by burgeoning RNA-seq and CyTOF technologies and taken in statistical ensembles of cells (i.e. cell populations) at multiple time points during a biological time course. *I*_*C*_ captures the information immanent in both the *m* gene vectors (the expression level of a gene across a large number *n* of individual cells) and the *n* cell vectors. Thus, *I*_*C*_ does not require time-continuous monitoring of fluctuations as in many studies of critical state transitions because the information needed is in the high dimensionality (*m*) and in the statistical ensemble (*n*). Although the decrease of cell–cell correlation (denominator of *I*_*C*_) as a sign of attractor destabilization is intuitively obvious, *I*_*C*_ is not an ad hoc statistical measure that identifies patterns in the data but is grounded in first principles of non-linear dynamical systems [46], as is particularly evident in the numerator, the gene–gene correlation. Conceptually, it is possible to speculate on a correspondence between the increase of gene–gene correlation to the appearance of long-range correlations of state variables in time (autocorrelation) and/or space used in the classical phenomenological analyses of critical state transitions [11] or of stressed populations [31], although such an equivalence has yet to be formally shown. A recent study also examined the approach to the bifurcation as a critical transition and its phenomenological manifestation in the presence of stochastic fluctuations, albeit in a continuous two-dimensional system representing a version of the canonical toggle switch with two mutually suppressing genes. The authors found that not only the cell diversity increases but also the anticorrelation of the two genes [20].

On the biology of cell fate commitment, we show that bifurcation dynamics and single-cell expression analysis naturally integrate the two opposing classical models of cell fate determination [38]: instruction by extrinsic factors that explicitly regulate the gene expression change in each cell so as they adopt a particular cell fate [42] and selection of cells that have entered an intrinsically predestined gene expression state (attractor) [37] by promoting their survival and proliferation. Both models are supported by our observations. The deterministic bias toward a prospective lineage in the destabilization of the progenitor state, as manifest in the early changes of the transcriptome (Fig 5A), confirms that instruction plays a role in fate determination. Conversely, the survival of the rebellious cells when the appropriate growth factors are later provided (to promote the non-intended fate) exposes a role for selection in cell lineage determination (Fig 3C).

In the case of selection, the promotion of one differentiation fate over the other is straightforward. But how does instruction steer a cell toward the desired state if leaving the multipotent state is driven by attractor destabilization? What causes the asymmetry of outcome that ensures that cells in the desired lineage dominate over the rebellious cells? The term “biased destabilization” used herein is more than a metaphoric explanation. It is grounded in the underlying bifurcation dynamics and could be elaborated in formal ways if one were able to explicitly model the dynamics of the relevant gene circuit for which many generic theoretical models have been proposed [8,15–20]. In the simplest case, binary lineage branching has often been modeled as a symmetrical supracritical pitchfork bifurcation [47]; here, the instructive signal could alter the values of other parameters in addition to the bifurcation parameter, such that one of the two post-bifurcation stable steady states (depending on the signal) is more stable than the other. In an alternative class of models, the bifurcation is inherently asymmetric, e.g., modelled as an imperfect pitchfork bifurcation [48]. Such models are more realistic because these nonsymmetrical bifurcations are structurally robust. Here, one of the two post-bifurcation stable states is detached from the destabilizing progenitor attractor state and, thus, is less accessible (requiring stronger gene expression noise) than the other, which can be directly accessed from the vanishing progenitor attractor, as depicted in landscape diagrams, as in Fig 6 and references [11–13]. The instructive signal could introduce the imperfection, such that either one of the new attractors becomes separated. Future detailed analysis using measurements of single cell states at much higher time-resolution could address these questions and distinguish between these possibilities.

In this study, we did not consider cell-to-cell communication mediated by local and soluble signals, which can modulate the internal GRN dynamics and, hence, affect phenotype transitions. This is an important aspect because the nonlinear, high-dimensional dynamics of cell states driven by a GRN poises cell populations at metastable states, making them akin to an excitable medium that, upon external perturbation, can respond in nonintuitive ways, as most lucidly epitomized by the rebellious cells. This response introduces cell population heterogeneity, underscoring the importance of cell population dynamics in which subpopulations coexist and shift their relative abundances in many ways that are affected by cell–cell communication between cells of distinct types. Indeed, a transition from cell type A to cell type B often does not follow first-order kinetics, suggesting non-cell autonomous effects (S.H., unpublished observation). For instance, cells that have reached an attractor state may secrete signals that can either promote or suppress the transition into it. A cell–cell interaction network would add an additional layer of dynamics to that of the intracellular GRN. The resulting (multiscale) cell population dynamics would be manifest as an accentuated modulation of the attractor landscape (Fig 6) but could still be captured using the phenomenological framework of critical transitions because attractor destabilization preceding the phenotype switch does not depend on the specific (physical) implementation of the bifurcation.

As single-cell resolution molecular profiling of cell states become routine, it will be important to analyze this new type of data, embodied by the matrix **X**(*t*), in a fashion that is hypothesis-driven or is informed by the underlying first principles of dynamical systems (Fig 6), as opposed to solely using the current palette of ad hoc computational data analytics tools to find cell clusters and reduce dimensionality [46]. Although still phenomenological, invoking concepts of critical transitions provide a formal link to the fundamental principles.

It remains to be seen whether critical transitions can be as readily detected in other cell systems as in the few examples examined in this study. Moreover, the significance for diseases and response to drugs of cell population dynamics with instabilities and critical behaviors could be further elucidated as single-cell analysis becomes commonplace and we move beyond descriptive data analysis. The notion of critical behaviors could be of practical utility for predicting major shifts in cell populations and tissues relevant in development and disease using data from single-resolution measurements at multiple discrete time points.

**NOTE ADDED IN PROOF: During the submission of this manuscript we became aware of the work of Richard, et al., 2016 (doi:** **10.1371/journal.pbio.1002585****) which was motivated by a different conceptual framework and used different analysis methods but arrived at a similar conclusion**.

## Materials and Methods

### Culture and Differentiation of EML Cells

Blood progenitor EML cells (ATCC CRL-11691) were cultured and maintained as described previously [26]. Multipotent EML cell population was stimulated with either EPO (to differentiate into erythroid cells), GM-CSF/IL-3 and ATRA (to obtain myeloid cells), or a mixture of all cytokines for the “combined” treatment as previously reported [4,26]. Wright-Giemsa staining was performed with some modification following a reported protocol [27]. In brief, 60,000 cells in 250 μl of PBS + 1% FBS buffer were cytospun at 350 rpm for 5 min per slide and allowed to air dry for 10 min. Slides were subjected to five 1-second dips in methanol, followed by Wright-Giemsa staining solution (0.4% [w/v], Sigma). After a final rinse with water, slides were allowed to air dry for 30 min. Colored phase contrast images were obtained using a Zeiss Axiovert 200M microscope.

### Flow Cytometry and Fluorescence-Activated Cell Sorting (FACS)

Cell surface protein immunostaining and flow cytometry measurements were performed using established methods [4]. Briefly, the antibodies Sca1-PE (BD Pharmingen #553335), ckit-FITC (BD Pharmingen #553355), and CD11b-FITC (BD Pharmingen #557396) were used at 1:1,000 dilutions in ice-cold PBS containing 1% fetal calf serum with (flow cytometry) or without (FACS) 0.01% NaN3. Appropriate unstained and single-color controls were used for gate definition and compensation setup. Isotype control antibodies (BD Pharmingen #553988 for FITC and #553930 for PE isotype) were used to establish the background signal caused by nonspecific antibody binding. Propidium iodide (Roche #11348639001) staining was used to identify dead cells that were removed from analyses. Flow cytometry analysis was performed on a BD FACSCalibur cell cytometer with 10,000 viable events for each sample. Data were acquired using CellQuest Pro (BD) software and analyzed in FlowJo.

For FACS sorting, the Sca1 protein distribution was measured and the expression distribution was gated into three regions according to the Sca1 expression level as Sca1-Low, Mid, and High on day 0, 1, and 6 or four regions on day 3 after differentiation initiation (Fig 1A). Single cell sorting was conducted on a BD Biosciences FACSAria III in lysis buffer (see below). For myeloid differentiation, cells were stained with antibodies for both Sca1 and CD11b protein markers, and cell subpopulations were gated as illustrated in Fig 3A. For studies involving the dynamics of sorted subpopulations, antibodies were removed after sorting using brief incubation in a low-pH buffer [4].

### Single-Cell Gene-Expression Analysis Using OpenArray qPCR

Single cells were directly sorted into 5.0 μl of lysis buffer (CellsDirect kit, Invitrogen) containing 4.25 μl Resuspension Buffer and 0.25 μl Lysis Enhancer using a FACSAria III (BD Biosciences). 0.5 μl RNaseOut (Invitrogen) was added to the lysis solution to protect the RNA from degradation. To ensure that liquid droplets containing single cells were deposited at the center of the well and not at the wall, the position was checked on the plastic film covering the PCR plate. To reduce the possibility of cells sticking to the wall of the PCR well plate, we used low-binding PCR plates (Axygen, #6509). As control sample, a small population of 100 cells were sorted into a single well for qPCR analysis. To test for contamination of sorted cells with mRNA from lysed dead cells, 5.5 μl liquid from the FACS instrument was collected and analyzed. After sorting, the samples were heated to 75°C for 10 min to accelerate the lysis process, and samples were stored at -80°C. From these single-cell lysate samples, cDNA was directly synthesized as described previously [26]. The obtained cDNA was preamplified by 18 cycles [26] and subsequently diluted with Tris-EDTA buffer at a ratio of 1:10, resulting in templates for the real-time PCR analysis. This protocol led to fewer than 30 quantification cycles (C_q_) during the single-cell qPCR analysis on an OpenArray system (Life Technologies). On this system, each qPCR plate consists of 12×4 subarrays and each subarray contains 8×8 reaction chambers of 33 nl volume (S7A Fig) [28]. Each sample was divided into a subarray with 64 reaction chambers prior to qPCR quantification. No-template (water) control was also run on each plate to check for nonspecific products and/or presence of contaminants in the master mix. Following the amplification, the corresponding curves and C_q_ values were processed using the OpenArray Real-Time qPCR Analysis software (version 1.0.4) with a quantification threshold of 100(+/-5). Specific PCR primers were preimmobilized in the chambers (S7B Fig) and released in the first cycle by heat. For each subarray, 2 μl of target sample was loaded into each well of a 384-well plate (Applied Biosystems); subsequently, 3 μl of the master mix reaction consisting of TaqMan OpenArray Real-time PCR Master Mix (Applied Biosystems) was added to each well. Target and master mix were combined and centrifuged, and the 384-well plate was processed in the OpenArray AccuFill system (Applied Biosystems). During processing, 2.1 μl of the reaction solution was transferred automatically from each well into the corresponding subarrays of a qPCR plate, where the reaction solution retains into the reaction wells due to the differential hydrophilic–hydrophobic coating between wells and surface of the qPCR array [28]. The qPCR step was performed using thermocycling conditions of 50°C for 2 min, 95°C for 10 min, 40 cycles of 95°C for 15 sec, and 60°C for 1 min.

### Testing Taman qPCR Assays

We used off-the-shelf primers designed by Applied BioSystems (Life Technologies) for the analysis. The primers are usually designed to span exon–exon junction to target multiple splice variants of one transcript and to target only and specifically the gene of interest, avoiding amplification of genomic DNA. S3 Table lists all genes of interest, the inventoried TaqMan assay IDs (Applied Biosystems), and further relevant information when the manufacturer does not provide primer and probe sequences. To evaluate qPCR assay performance, calibration (standard) curves were generated by performing qPCR on a serial dilution of a prepared template. Each of these dilutions was dispensed into two subarrays of OpenArray plate, leading to six technical qPCR replicates for each single cell sample. To minimize the effect of sampling errors on quantification precision, only sample/assay combinations with at least three quantifiable replicates were considered for preparing the standard curves. The GAPDH assay was not preimmobilized on OpenArray plate but was independently tested on BioRad qPCR platform.

### Analysis of Single-Cell Gene Expression Data

Data analysis is described in more details in S1 Appendix. Single-cell expression data were initially analysed with OpenArray qPCR analysis software. For quality control, amplification curves were quality filtered and Ct thresholds were set for each assay with the same thresholds used across all experiments and cell populations. Data were subsequently exported to Excel as csv files. All of Cq values are available in S1 Data. Samples not expressing any gene were excluded from the analysis. Experimentally determined LODs were used as cutoff Cqs (S3 Table). Each assay was performed in triplicates, and the median of the triplicates was used for subsequent analysis. After this preprocessing, ΔCq was calculated as previously described [29]. Higher level of analysis such as correlation, clustering, and PCA was performed on log2-transfromed expression data.

### Gene Expression Profiling with Microarrays and Data Analysis

Microarray analyses were performed by the Vancouver Prostate Centre. EML progenitor cell population was stimulated with EPO alone, IL-3/GM-CSF alone, or a combination of all cytokines. On d3 and d6 after stimulation with different cytokines, the main “peaks” in the Sca1 distribution were gated and cell subpopulations were sorted using FACSAria III. Fig 5A illustrates the experimental design for the microarray experiments. Total RNA was extracted from 1×10^6^ of sorted subpopulations using mirVana miRNA Isolation Kit (Ambion) following the manufacturer’s instructions. Genomic DNA was removed from the isolated and purified RNA using DNase I. Total RNA quality was assessed with the Agilent 2100 Bioanalyzer prior to microarray analysis. Samples with a RIN value equal to or greater than 8.0 were deemed acceptable for microarray analysis. Samples were prepared following Agilent’s One-Color Microarray-Based Gene Expression Analysis Low Input Quick Amp Labeling v6.0. An input of 100 ng of total RNA was used to generate Cyanine-3 labeled cRNA. Samples were hybridized on Agilent SurePrint G3 Mouse GE 8x60K Microarray (Design ID 028005). Arrays were scanned with the Agilent DNA Microarray Scanner at a 3 μm scan resolution, and data was processed with Agilent Feature Extraction 11.0.1.1. To filter out genes that were not expressed above the background noise, a raw intensity cutoff value of 25 was applied because the correlation between the technical replicates decreases for higher levels. Green processed signal was quantile-normalized using the “normalize.quantiles” function in R that takes care of inter-chip variability. To filter out genes which did not change between the samples, the distribution of each gene across all samples was analyzed. Therefore, the standard deviation (STD) distribution was calculated and only genes with STD > 10% were selected. As a result, 6,297 genes passed the criteria and were selected as the top 10% of genes among the samples. Self-organising maps (SOM) of the top 10% of most varied genes (6,297 genes) were generated using the Gene Expression Dynamics Inspector program (GEDI) [44]. Cluster analysis was performed using the “clustergram” function in Matlab R2012a Bioinformatics toolbox using hierarchical clustering with Euclidean distance metric and average linkage to generate the dendrogram. Input data was log2-tranformed values of normalized fluorescent intensity signals for genes of interest extracted from the samples and plotted as a heatmap. Data represented the average of *n* = 2 independent biological replicates. The normalized fluorescent intensity values of 17 genes of interest in the curated network were extracted from each sample.

## Supporting Information

S1 Fig Manually curated model of gene regulatory network governing fate decision of CMP. Network of experimentally verified regulatory interactions of transcription factors involved in multipotency of the CMP state, fate decision and differentiation to the erythroid and myeloid lineages (S1 Table). The canonical GATA1-PU.1 circuit is highlighted in green. A few surface markers including c-kit (progenitor, grey box), EpoR (erythroid, red box) and CD11b (myeloid, blue box) were included in the network to control the cell differentiation behavior and used as markers for lineage commitment in experiments. The numbers point to the row in the S1 Table that contains the references.
(JPG)

S2 Fig Gene expression profile of single-cell samples during differentiation. Expression profiles of 17 transcription factors and control genes (rows) in individual cells (columns) are visualized as a heatmap. Cell columns are arranged for days d1, d3 and d6 with respect to different treatments where grey shades correspond to untreated progenitors (d0), red shades to EPO treatment, blue shades indicate cells treated with GM-CSF/IL-3 and purple shades to combined treatment EPO+GM-CSF/IL-3 cytokines. The different shades of each color indicate the different Sca1 marker expression levels Sca1^Low^ (L), Sca1^Mid^ (M) and Sca1^High^ (H) determined during FACS sorting where darker shades denote higher Sca1 expression. Gene rows were ordered according to their biological role as indicated on the left.
(JPG)

S3 Fig Technical noise associated with single-cell RT-qPCR is significantly smaller than biological cell-cell variability. **(A)** Quantification cycles (Cq) of 80 individual EML cells for GATA1 expression is reported. Values are means ± STD for up to 128 technical replicates. **(B)** Quantification cycles (Cq) of up to 110 technical replicates are presented for 3 selected single-cells. Single-cell Cqs of biological samples clearly show a broader distribution relative to that of technical replicates. **(C)** Box plots represent the variability in terms of CV for technical replicates averaged over 110 realizations of the real-time PCR-steps on the ds-cDNA and the distribution of CV across all 80 individual EML progenitor cells for the GATA1 expression. The biological variation was significantly larger than the technical noise (p-value 2.2e-28, Mann-Whitney U test). Similar results were obtained for PU.1 (not shown).
(JPG)

S4 Fig Distinct trajectories of cell differentiation are observed upon stimulation of progenitor cells with cytokines in the PCA state space. Principal component projections in a total of ~1600 haematopoietic cells including progenitor (black), single-EPO treated (red-shades), single-IL3/GM-CSF treated (blue-shades) and combined-treated (purple-shades) in the first three components determined from expression of all 17 transcription factors and endogenous control genes. **(B)** Principal component loadings for PC 2 and 3 indicate the extent to which each gene contributes to the separation of cells along each component. **(C)** PCA weights of genes for the first three PCs reveals the importance of the individual genes to explain the difference between the different treatments and corresponding cell fate. **(D)** Cells in their attractor states still exhibit heterogeneous transcription profiles that can be traced back to individual genes. Cells treated with GM-CSF/IL-3 for 6 days are clearly located within the state space defined by the myeloid genes and cells treated by EPO exhibit 2 clusters where the lower one is governed by erythroid genes and the higher one by stemness genes. **(E)** Variance explained by principal components show that the first three components jointly explain more than 70% of variation in the data.
(JPG)

S5 Fig Gene expression in individual cells from the progenitor population and the α, β, and γ subpopulations. **(A-D)** Heatmap representation of gene expression profiles for the set of 17 genes of the curated network and 2 endogenous genes as control in total 216 single cells including 72 progenitor cells (panel A) and 48 single cells from each of the three subpopulations in the tri-modal Sca-1 population distribution on day 3 after GM-CSF/IL-3 treatment (Fig 3 in main text), α (B) β (C) and γ (D). Genes are ordered according to their reported biological role, as erythroid-associated (red box), stemness (green box), myloid-associated (blue box) and endogenous genes in all subplots. Based on the expressed genes, the β subpopulation seems to be committed to the myeloid lineage while the γ subpopulation is committed to the erythroid lineage. The α subpopulation exhibits an indeterminacy with a bias towards the myeloid lineage. **(E)** PCA of all attractor cells (d0 and d6) as shown in the S4 Fig combined with the cells from the α (yellow), β (green), and γ (pink) subpopulations support the above described similarity to the untreated EML, the GM-CSF/IL-3 stimulated and the EPO-stimulated cells, respectively. **(F)** Coefficient of variation CV of expression levels of distinct genes is used as a cell-specific quantity to expose population dispersion and has no direct physical meaning; it was calculated for each cell from the expression levels across all genes for each subpopulation. Histograms represent the number of cells at different level of the CV measure and show that cells in α subpopulation have higher spread of cellular CV values.
(JPG)

S6 Fig Single-cell gene expression analysis: PCA from Fig 1 recolored for origin in the Sca1 population fraction. Colors of cells again (as in Fig 1) indicate treatment, but in addition, their provenience from the respective Sca-1 fraction in the progenitor population (d0). Rebellious cells are cells that shift toward the non-intended fate, i.e. EPO-treated cells moving towards the destination region of the differentiated myeloid cells in the top/right region and IL-3/GM-CSF-treated cells moving toward the prospective destination region of the differentiated erythroid cells in the bottom left region (see day 6 in top panel). To illustrate that the rebellious are recruited from the respectively primed states, the Sca1-fraction (LOW, MID, HIGH) in the progenitor population from which the cells originated was indicated by the color hue (see legend on left) for the day 3 stage: the darker, the higher was d0-Sca1 expression. Note that the perspective is slightly shifted from that of Fig 1 to show that there is no clean separation at d3 into two disjoint cluster. As previously noted (see MAIN TEXT), the Sca1^HIGH^ cells are primed towards the myeloid cells, whereas Sca1^LOW^ cells are primed towards the erythroid cells. Note that although at this time point at d3 the cloud spreads and begins to split, and there is no separation between IL-3/GM-CSF and EPO treated cells. However, rebellious cells tend to originate from the Sca1-fraction for which they are primed. For instance, Sca1^LOW^ cells in the progenitor population which are known to be primed towards erythroid fate [4], are the source of the rebellious cells which despite treatment with IL-3/GM-CSF move towards the erythroid direction at d3 (light blue cells). Thus, priming determines fate more than instruction by the external signal.
(JPG)

S7 Fig Representation of an OpenArray plate used for single-cell qPCR. **(A)** Each OpenArray (Applied Biosystems) is the size of a microscope slide. It holds 48 groups (subarrays, red rectangular) of 64 holes of 33 nl volume in which one PCR reaction occurs. A hydrophilic layer is at the interior surface of each hole and a hydrophobic layer is at the exterior surface of the plate allowing for filling the hole by surface tension. In total, each array carries 3072 qPCR reactions. **(B)** Specific PCR primers are pre-immobilized in individual holes (by manufacturer, for customized assay patterns) and released by heat in the first cycle. **(C)** An example of the distribution of single-cell samples (SC) along with NTC (no template water control), IRC (inter-run calibrator) and 100-cell control (PC) samples on an OpenArray chip.
(JPG)

S8 Fig Quality control of single-cell qCPR. **(A)** Inter-chip variability is evaluated using inter-run calibrator (IRC) sample. Each curve represents the distribution of Cq values of each gene across all OpenArray chips. The flat black curve represents the distribution of all genes across all chips. The inter-gene differences are up to 2 orders of magnitude larger than the inter-chip variability of the same gene. The inter-run calibrator was a 10-fold diluted sample of 18 cycles pre-amplified cDNA of 10 ng isolated RNA from EML progenitor cell population. **(B-D)**. Correlation between gene expression in an ensemble of 48 individual cells and 6 replicates of 100-cell pools is plotted. Cells used were from subpopulations α, β and γ (subplots b-d) as presented in Fig 3 and 19 genes as listed in S3 Table were measured in triplicate in all single cells and bulk (100-cell) samples from each subpopulation. Mean expression for each gene was calculated across all single cell or pool samples. Note that the scaled mean expression for 100-cells pool was plotted against mean expression for single-cells. In all cases a high correlation between single-cell data and bulk data with correlation coefficient of > 0.86 was observed.
(JPG)

S1 Table Regulatory interactions in the curated GRN model of binary fate decision in CMP. Table of the regulatory interactions (either activating (A) or inhibiting (I)) between the genes. For each interaction, the literature is referenced (numbered list in the right panel). All interactions have been reported in for murine hematopoiesis.
(JPG)

S2 Table Quantified dissimilarity between transcriptomes from micro-arrays between samples. Pair-wise dissimilarity between expression profiles (samples) was calculated based on the normalized gene expression levels for 6297 filtered genes (see Material and Methods) with 1–*R* where *R* is the Pearson’s correlation coefficient which ranges from 0 to 1, meaning that 0 correspond to highest similarity and 1 to most different expression. Bootstrapping was performed by randomly selecting 30% of the genes in any sample to calculate the pair-wise dissimilarity metric and repeating the procedure 10,000 times to generate the reported standard deviations.
(JPG)

S3 Table Evaluation of qPCR assays. Table lists all primer pairs and relevant information including IDs and amplicon length. All assays were inventoried. Identical PCR primers were used in the pre-amplification step and the subsequent singleplex qPCR step. In addition, the amplification efficiency and limit of detection (LOD) of the qPCR assays are given. To evaluate efficiency and LOD, a 1:2 serial dilution was prepared from 18 cycles pre-amplified product from 10 ng RNA purified from EML progenitor cell population. Amplification efficiency was calculated according to: [10^(1/-S)-1] × 100%. The slope was obtained by linear regression of the standards curve. Efficiency was determined as average of two biological replicates with 6 qPCR technical replicates each. The Cq value for the LOD is defined as the most diluted sample that results in positive amplification for 5 out of 6 replicates.
(JPG)

S1 Data Single-cell and 100-cell samples quantification cycles (raw) data. The quantification cycles (Cq's) for all analyzed single-cells as well as 100-cell-pool control samples are reported. Single cells from untreated EML control cells as well as EML cells treated with EPO, GM-CSF/IL-3 or a combination of all cytokines on d1, d3 and d6 of stimulation. Gene expression data for single-cell samples sorted from α, β and γ subpopulations generated upon GM-CSF/IL-3 treatment of EML are also included. 6 replicates of the 100-cell samples were also sorted from each fraction and/or subpopulation and analyzed as control.
(XLSX)

S2 Data The numerical data that underlie the graphs in the figures in the main text. The relevant data used in the figures are in separate spreadsheets within this file, with the sheet names (tabs) indicating the associated figure.
(XLSX)

S3 Data Flow cytometry data for Fig 4 of main text. FCS-format data files for individual histograms of the two time courses (untreated and IL-3/GM-GM-CSF treated) as shown in Fig 4. Each folder represents a time point for the untreated and the IL-3/GM-CSF treated cells and contains two files for the sample histograms for Sca1 and the PI stained cells, respectively for the given time point. The day 0 (d0) folders also contain the data for the isotype control (3 files). The day 1 (d1) folders also contains the pre-sorting samples for both PI and Sca1 stained cells (4 files). Acronyms in folder/filenames:—‘EML’ = untreated EML cells—‘IL-3+GM-CSF’ or ‘MYL’ = cells treated to induce myeloid commitment—‘d3’ or ‘D3’ = flow cytometry performed at day 3 after treatment, as explained in the text—‘PI’ = stained with Propidium iodide (dead cells)—‘SCA1’ = stained for SCA1 Surface expression—‘LOW’ = cells sorted from the SCA1LOW fraction at d1 and recultured—‘UNSORTD’ = non-sorted cells as control.
(ZIP)

S1 Appendix Data Analysis. (PDF)

S2 Appendix Derivation of index *I*_C_ and pedagogical explanations. (PDF)

S3 Appendix Additional support from analysis of public data. (PDF)

## Abbreviations

EPO: erythropoietin
FACS: fluorescence-activated cell sorting
GM-CSF: granulocyte macrophage-colony stimulating factor
GRN: gene regulatory network
IL-3: interleukin-3
PCA: principal component analysis
PC: principal components

## References

[1] Macarthur BD, Ma’ayan A, Lemischka IR. Systems biology of stem cell fate and cellular reprogramming. Nat Rev Mol Cell Biol. 2009; 10:672–81. 10.1038/nrm2766 19738627 PMC2928569

[2] Huang S. Cell lineage determination in state space: a systems view brings flexibility to dogmatic canonical rules. PLoS Biol. 2010; 8(e1000380):1–4. 10.1371/journal.pbio.1000380 PMC2876052 20520792

[3] Wray J, Kalkan T, Smith AG. The ground state of pluripotency. Biochem Soc Trans. 2010; 38:1027–1032. 10.1042/BST0381027 20658998

[4] Chang HH, Hemberg M, Barahona M, Ingber DE, Huang S. Transcriptome-wide noise controls lineage choice in mammalian progenitor cells. Nature. 2008; 453:544–7. 10.1038/nature06965 18497826 PMC5546414

[5] Hough SR, Laslett AL, Grimmond SB, Kolle G, Pera MF. A continuum of cell states spans pluripotency and lineage commitment in human embryonic stem cells. PLoS ONE. 2009; 4(11):e7708 10.1371/journal.pone.0007708 19890402 PMC2768791

[6] Pina C, Fugazza C, Tipping AJ, Brown J, Soneji S, Teles J, et al Inferring rules of lineage commitment in haematopoiesis. Nat Cell Biol. 2010; 14:287–94. 10.1038/ncb2442 22344032

[7] Kauffman S. Homeostasis and differentiation in random genetic control networks. Nature. 1969; 224(215): 177–178. 5343519 10.1038/224177a0

[8] Huang S, Guo YP, May G, Enver T. Bifurcation dynamics of cell fate decision in bipotent progenitor cells. Dev Biol. 2007; 305:695–713. 10.1016/j.ydbio.2007.02.036 17412320

[9] Kaplan D, Glass L. Understanding Nonlinear Dynamics New York, Springer; 1995.

[10] Perko L, Differential Equations and Dynamical Systems Texts in Applied Mathematics. Marsden JE, Sirovich L, Golubisky M. editors. New York, Springer Book; 2006; 7:557.

[11] Scheffer M, Carpenter SR, Lenton TM, Bascompte J, Brock W, Dakos V, et al Anticipating critical transitions. Science. 2012; 338:344–8. 10.1126/science.1225244 23087241

[12] Scheffer M, Bascompte J, Brock WA, Brovkin V, Carpenter SR, Dakos V, et al Early-warning signals for critical transitions. Nature. 2009; 461(7260):53–59. 10.1038/nature08227 19727193

[13] Trefois C, Antony PM, Goncalves J, Skupin A, Balling R. Critical transitions in chronic disease: transferring concepts from ecology to systems medicine. Curr Opin Biotechnol. 2015; 34:48–55. 10.1016/j.copbio.2014.11.020 25498477

[14] Huang S, Eichler G, Bar-Yam Y, Ingber DE. Cell fates as high-dimensional attractor states of a complex gene regulatory network. Phys Rev Lett. 2005; 94(12):128701 10.1103/PhysRevLett.94.128701 15903968

[15] Chickarmane V, and Peterson C. A computational model for understanding stem cell, trophectoderm and endoderm lineage determination. PLoS ONE. 2008; 3:e3478 10.1371/journal.pone.0003478 18941526 PMC2566811

[16] Duff C, Smith-Miles K, Lopes L, Tian T. Mathematical modelling of stem cell differentiation: the PU.1-GATA-1 interaction. J Math Biol. 2011; 64(3):449–68. 10.1007/s00285-011-0419-3 21461760

[17] Ferrell JE. Bistability, bifurcations, and Waddington's epigenetic landscape. Current biology. 2012; CB 22:R458–466. 10.1016/j.cub.2012.03.045 22677291 PMC3372930

[18] Jolly MJ, Huang B, Lu M, Mani SA, Levine H, Ben-Jacob E. Towards elucidating the connection between epithelial-mesenchymal transitions and stemness. J R Soc Interface. 2014; 11:20140962 10.1098/rsif.2014.0962 25339690 PMC4223923

[19] Furusawa C. Kaneko k. A dynamical-systems view of stem cell biology. Science. 2012; 338(6104):215–217. 10.1126/science.1224311 23066073

[20] Pal M, Ghosh S, Bose I. Non-genetic heterogeneity, criticality and cell differentiation. Phys Biol. 2014; 12(1): 016001 10.1088/1478-3975/12/1/016001 25429686

[21] Chen L, Liu R, Liu ZP, Li M, Aihara K. Detecting early-warning signals for sudden deterioration of complex diseases by dynamical network biomarkers. Sci Rep. 2012; 2:18–20. 10.1038/srep00342 PMC3314989 22461973

[22] Tsai S, Bartelmez S, Sitnicka E, Collins S. Lymphohematopoietic pro-genitors immortalized by a retroviral vector harboring a dominant-negative ret-inoic acid receptor can recapitulate lymphoid, myeloid, and erythroid develop-ment. Genes Dev. 1994; 8(23):2831–2841. 7995521 10.1101/gad.8.23.2831

[23] Paul F, Arkin Y, Giladi A, Adhemar Jaitin D, Kenigsberg E, Keren-Shaul H, et al Transcriptional Heterogeneity and Lineage Commitment in Myeloid Progenitors. Cell. 2015; 163(7): 1663–1677. 10.1016/j.cell.2015.11.013 26627738

[24] Ying QL, Wray J, Nichols J, Batlle-Morera L, Doble B, Woodgett J, et al The ground state of embryonic stem cell self-renewal. Nature. 2008; 453(7194):519–23. 10.1038/nature06968 18497825 PMC5328678

[25] Grun D, Kester L, van Oudenaarden A. Validation of noise models for single-cell transcriptomics. Nature methods. 2014; 11(6):637–40. 10.1038/nmeth.2930 24747814

[26] Marinov GK, Williams BA, McCue K, Schroth GP, Gertz J, Myers RM, et al From single-cell to cell-pool transcriptomes: stochasticity in gene expression and RNA splicing. Genome Res. 2014; 24(3):496–510. 10.1101/gr.161034.113 24299736 PMC3941114

[27] Wu AR, Neff NF, Kalisky T, Dalerba P, Treutlein B, Rothenberg ME. Quantitative assessment of single-cell RNA-sequencing methods. Nature methods. 2014; 11(1):41–6. 10.1038/nmeth.2694 24141493 PMC4022966

[28] Kim JK, Kolodziejczyk AA, Illicic T, Teichmann SA, Marioni JC. Characterizing noise structure in single-cell RNA-seq distinguishes genuine from technical stochastic allelic expression. Nat Commun. 2015; 6:8687 10.1038/ncomms9687 26489834 PMC4627577

[29] Zhou JX, Aliyu MDS, Aurell E, Huang S. Quasi-potential landscape in complex multi—stable systems. J R Soc Interface. 2015; 12:1–15. 10.1098/rsif.2012.0434 PMC3481575 22933187

[30] Waddington CH. Principles of Embryology. Allen Unwin Ltd 1956.

[31] Gorban AN, Smirnova EV, Tyukina T. Correlations, risk and crisis: From physiology to finance. Phys A Stat Mech. its Appl. 2010; 389:3193–3217.

[32] Wang J, Xu L, Wang E, Huang S. The potential landscape of genetic circuits imposes the arrow of time in stem cell differentiation. Biophys J. 2010; 99:29–39. 10.1016/j.bpj.2010.03.058 20655830 PMC2895388

[33] Giuliani A. Statistical Mechanics of Gene Expression Networks: Increasing Connectivity as a Response to Stressful Condition. Adv Syst Biol. 2014; 3:1–4.

[34] Treutlein B, Brownfield DG, Wu AR, Neff NF, Mantalas GL, Espinoza FH, et al Reconstructing lineage hierarchies of the distal lung epithelium using single-cell RNA-seq. Nature. 2014; 509(7500):371–375. 10.1038/nature13173 24739965 PMC4145853

[35] Giuliani A, Zbilut JP, Conti F, Manetti C, Miccheli A. Invariant features of metabolic networks: a data analysis application on scaling properties of biochemical pathways. Physica A. 2014; 337(1–2):157–170.

[36] Patel AP, Tirosh I, Trombetta JJ, Shalek AK, Gillespie SM, Wakimoto H, et al Single-cell RNA-seq highlights intratumoral heterogeneity in primary glioblastoma. Science. 2014; 344(6190):1396–1401. 10.1126/science.1254257 24925914 PMC4123637

[37] Enver T, Heyworth CM, Dexter TM. Do stem cells play dice? Blood. 1998; 92: 358–41; discussion 352. 9657728

[38] Enver T, Jacobsen SEW. Instructions writ in blood. Nature. 2009; 461. 10.1038/461183a 19741696

[39] Coffman RL, Reiner SL. Instruction, selection, or tampering with the odds? Science. 1999; 284:1283–1285. 10383307 10.1126/science.284.5418.1283

[40] Callard RE. Decision-making by the immune response. Immunol. Cell Biol. 2007; 85:300–305. 10.1038/sj.icb.7100060 17471303

[41] Veraart AJ, Faassen EJ, Dakos V, Van nes EH, Lurling M, Scheffer M. Corrigendum: Recovery rates reflect distance to a tipping point in a living system. Nature. 2012; 484:404–404. 10.1038/nature10723 22198671

[42] Metcalf D. Stem cells, pre-progenitor cells and lineage-committed cells: Are our dogmas correct? Ann N Y Acad Sci. 1999; 872:289–304. 10372131 10.1111/j.1749-6632.1999.tb08473.x

[43] Ogawa M. Hemopoietic stem cells: stochastic differentiation and humoral control of prolifera-tion. Environ Health Perspect. 1989; 80:199–207. 2647480 10.1289/ehp.8980199 PMC1567604

[44] Eichler GS, Huang S, Ingber DE. Gene Expression Dynamics Inspector (GEDI): for integrative analysis of expression profiles. Bioinformatics. 2003; 22;19(17): 2321–2. 14630665 10.1093/bioinformatics/btg307

[45] Metcalf D. Lineage commitment and maturation in hematopoietic cells: the case for extrinsic regulation. Blood. 1998; 92(2):345–347; discussion 352. 9657727

[46] Marr C, Zhou JX, Huang S. Single-cell gene expression profiling and cell state dynamics: collecting data, correlating data points and connecting the dots. Curr Opin Biotechnol. 2016; 39:207–214. 10.1016/j.copbio.2016.04.015 27152696 PMC5130334

[47] Zhou JX, Huang S. Understanding gene circuits at cell-fate branch points for rational cell reprogramming. Trends Genet. 2011; 27(2):55–62. 10.1016/j.tig.2010.11.002 21146896

[48] Liu P, Shi J, Wang Y. Imperfect transcritical and pitchfork bifurcations. J Funct Anal. 2007; 251(2): 573–600.

