# Supplementary figures and images for: Cell Fate Decision as High-Dimensional Critical State Transition
Source: PLoS Biol. 2016 Dec 27;14(12):e2000640. doi: 10.1371/journal.pbio.2000640 (PMC5189937; doi:10.1371/journal.pbio.2000640)

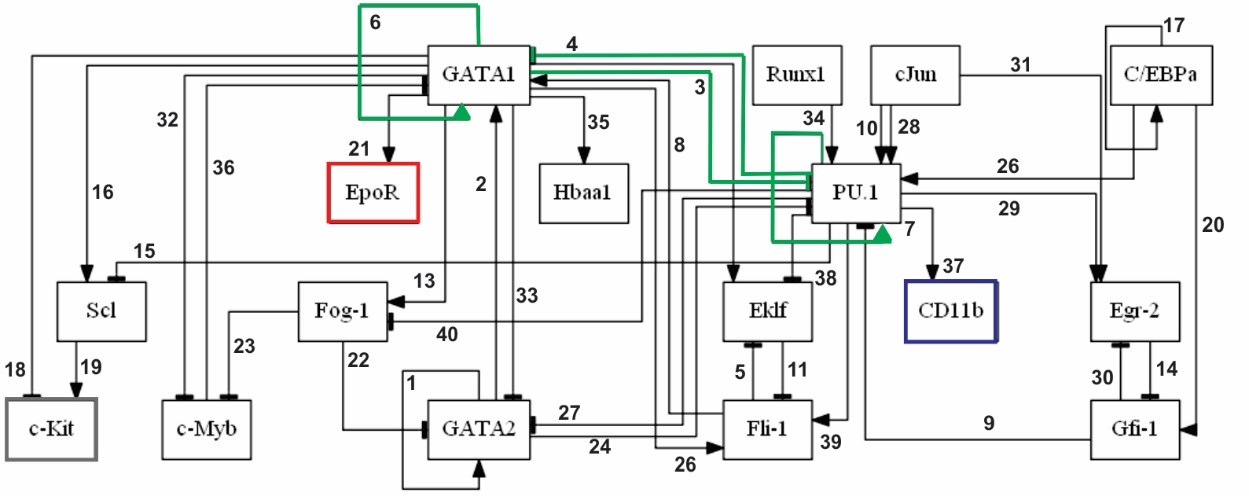

Supplement: S1 Fig — Network of experimentally verified regulatory interactions of transcription factors involved in multipotency of the CMP state, fate decision and differentiation to the erythroid and myeloid lineages (S1 Table). The canonical GATA1-PU.1 circuit is highlighted in green. A few surface markers including c-kit (progenitor, grey box), EpoR (erythroid, red box) and CD11b (myeloid, blue box) were included in the network to control the cell differentiation behavior and used as markers for lineage commitment in experiments. The numbers point to the row in the S1 Table that contains the references. (JPG) [file pbio.2000640.s001.jpg]

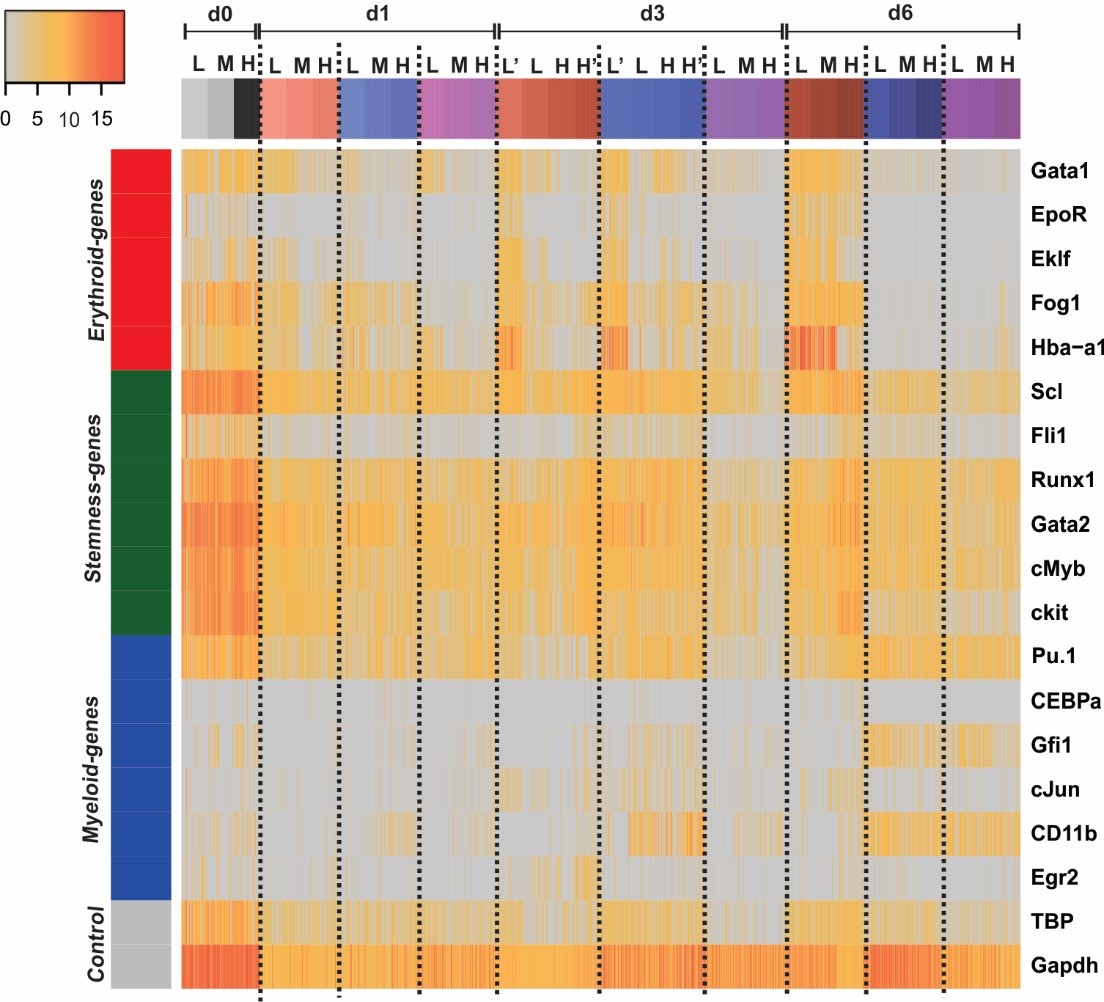

Supplement: S2 Fig — Expression profiles of 17 transcription factors and control genes (rows) in individual cells (columns) are visualized as a heatmap. Cell columns are arranged for days d1, d3 and d6 with respect to different treatments where grey shades correspond to untreated progenitors (d0), red shades to EPO treatment, blue shades indicate cells treated with GM-CSF/IL-3 and purple shades to combined treatment EPO+GM-CSF/IL-3 cytokines. The different shades of each color indicate the different Sca1 marker expression levels Sca1Low (L), Sca1Mid (M) and Sca1High (H) determined during FACS sorting where darker shades denote higher Sca1 expression. Gene rows were ordered according to their biological role as indicated on the left. (JPG) [file pbio.2000640.s002.jpg]

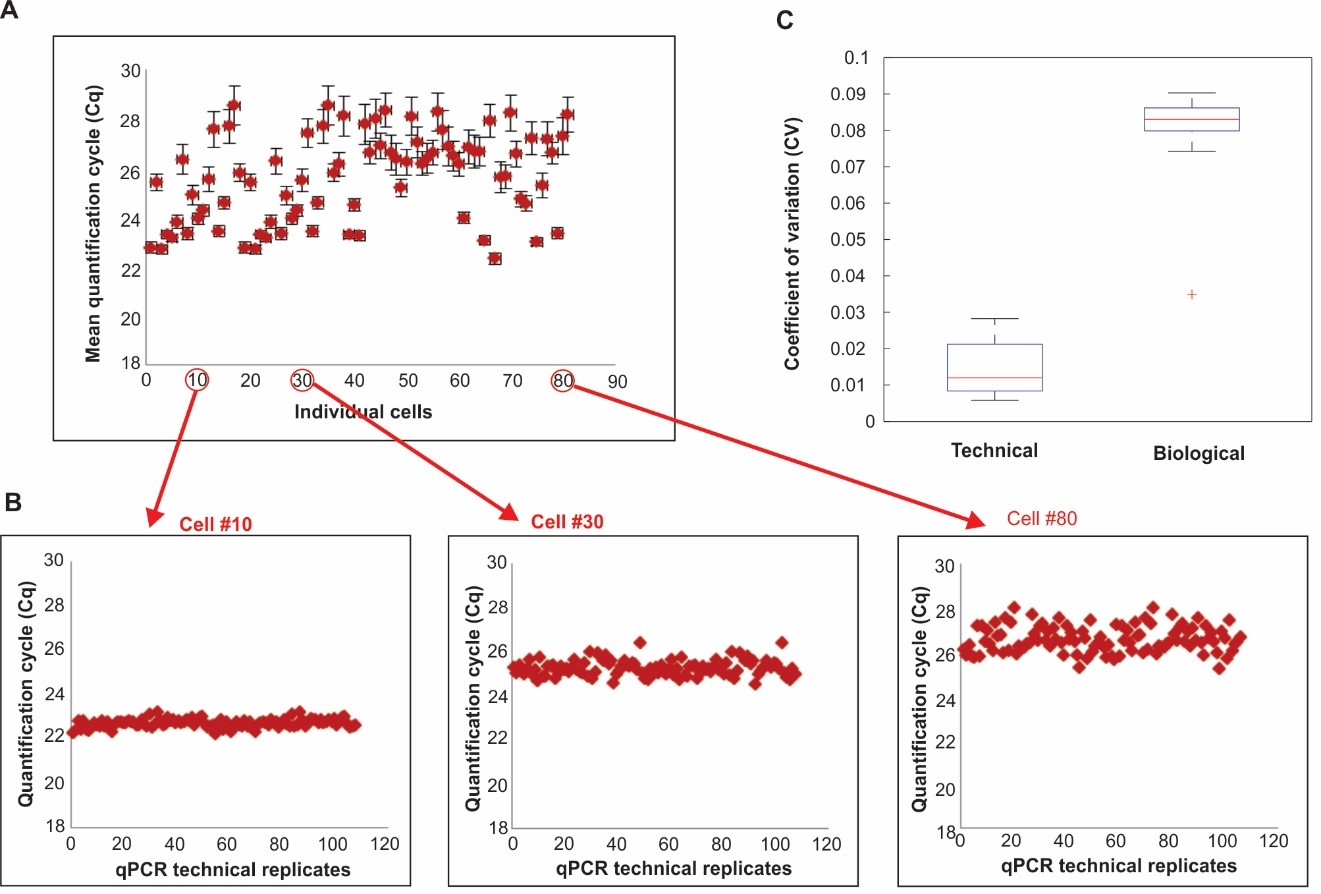

Supplement: S3 Fig — (A) Quantification cycles (Cq) of 80 individual EML cells for GATA1 expression is reported. Values are means ± STD for up to 128 technical replicates. (B) Quantification cycles (Cq) of up to 110 technical replicates are presented for 3 selected single-cells. Single-cell Cqs of biological samples clearly show a broader distribution relative to that of technical replicates. (C) Box plots represent the variability in terms of CV for technical replicates averaged over 110 realizations of the real-time PCR-steps on the ds-cDNA and the distribution of CV across all 80 individual EML progenitor cells for the GATA1 expression. The biological variation was significantly larger than the technical noise (p-value 2.2e-28, Mann-Whitney U test). Similar results were obtained for PU.1 (not shown). (JPG) [file pbio.2000640.s003.jpg]

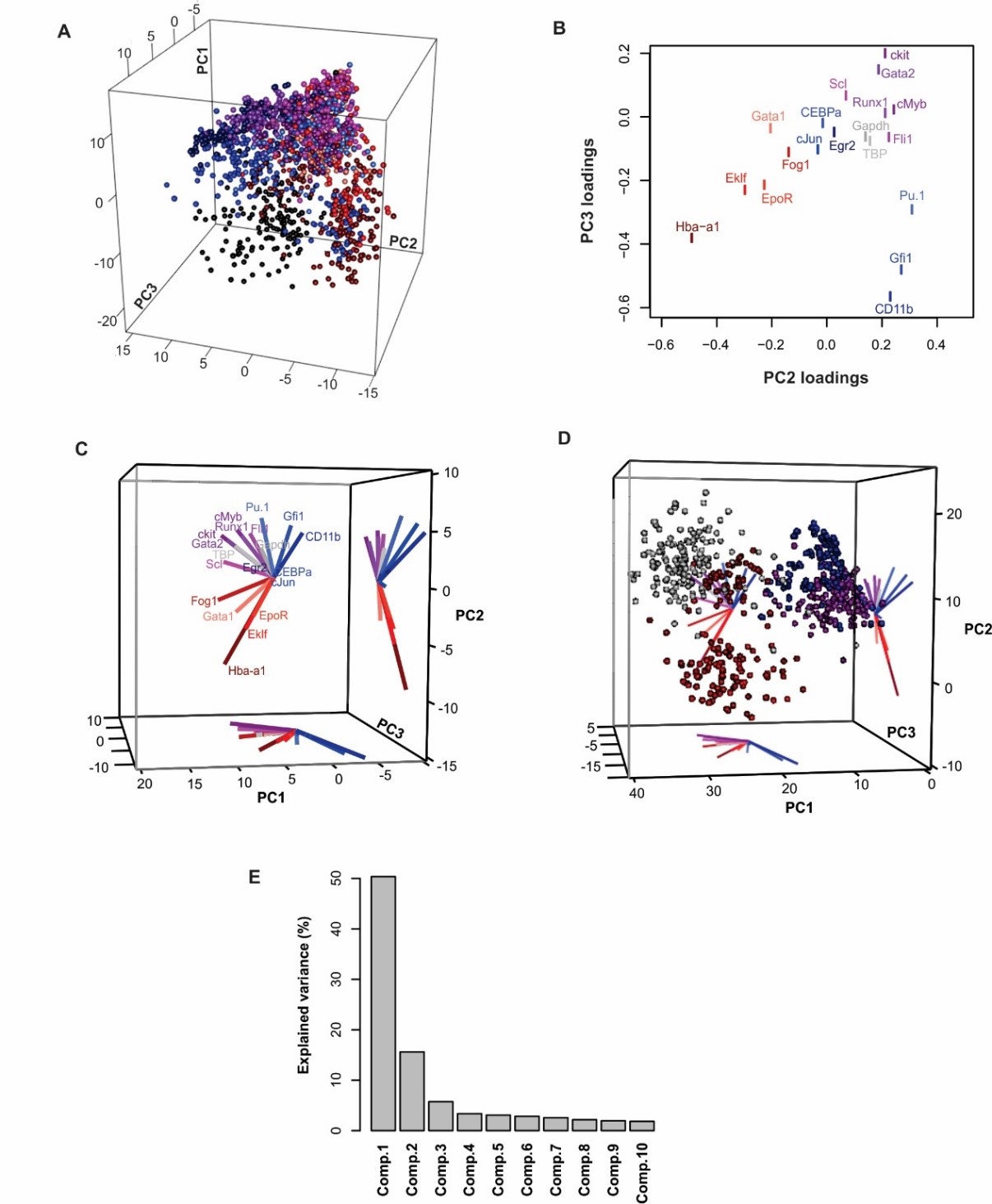

Supplement: S4 Fig — Principal component projections in a total of ~1600 haematopoietic cells including progenitor (black), single-EPO treated (red-shades), single-IL3/GM-CSF treated (blue-shades) and combined-treated (purple-shades) in the first three components determined from expression of all 17 transcription factors and endogenous control genes. (B) Principal component loadings for PC 2 and 3 indicate the extent to which each gene contributes to the separation of cells along each component. (C) PCA weights of genes for the first three PCs reveals the importance of the individual genes to explain the difference between the different treatments and corresponding cell fate. (D) Cells in their attractor states still exhibit heterogeneous transcription profiles that can be traced back to individual genes. Cells treated with GM-CSF/IL-3 for 6 days are clearly located within the state space defined by the myeloid genes and cells treated by EPO exhibit 2 clusters where the lower one is governed by erythroid genes and the higher one by stemness genes. (E) Variance explained by principal components show that the first three components jointly explain more than 70% of variation in the data. (JPG) [file pbio.2000640.s004.jpg]

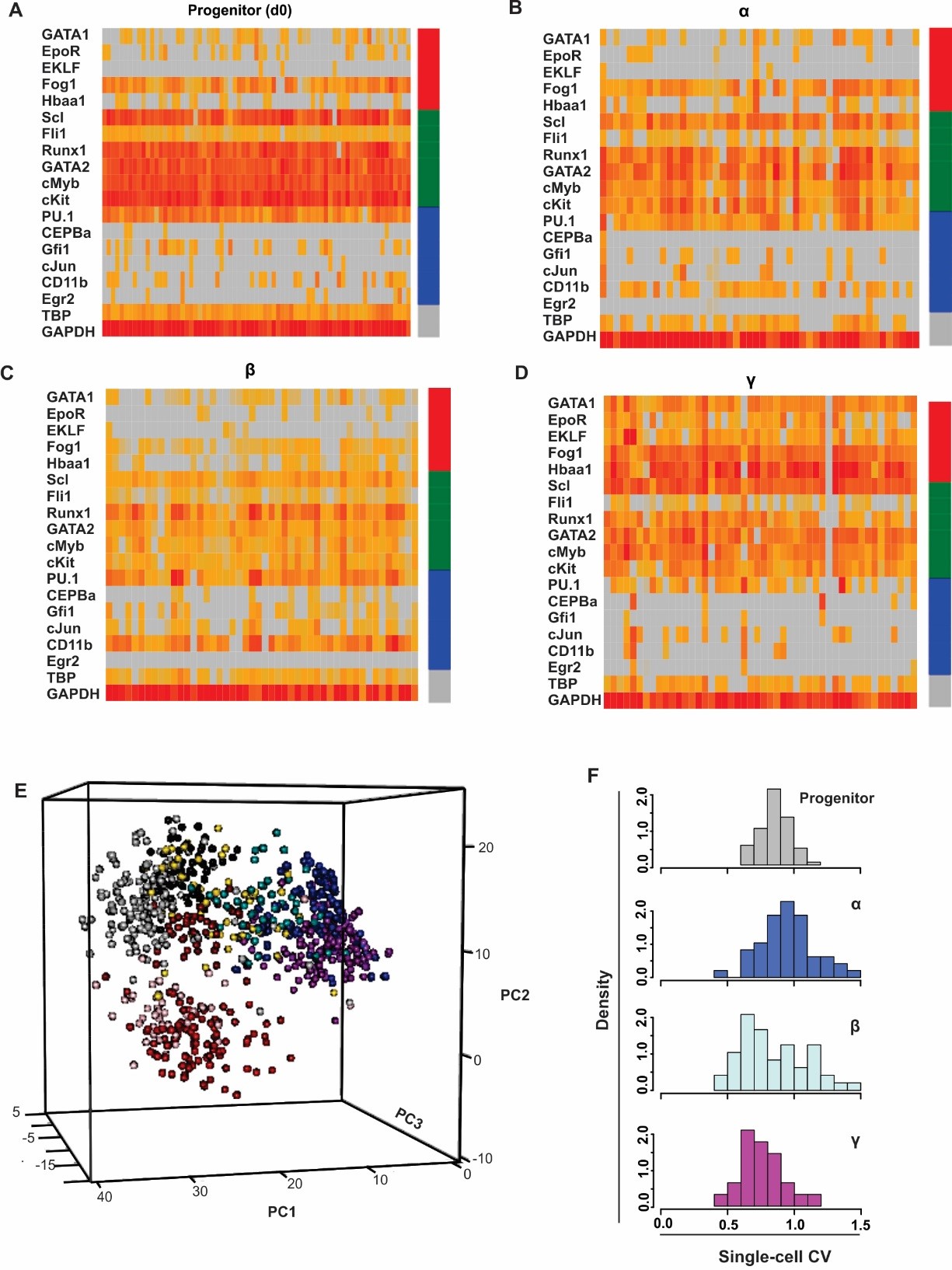

Supplement: S5 Fig — (A-D) Heatmap representation of gene expression profiles for the set of 17 genes of the curated network and 2 endogenous genes as control in total 216 single cells including 72 progenitor cells (panel A) and 48 single cells from each of the three subpopulations in the tri-modal Sca-1 population distribution on day 3 after GM-CSF/IL-3 treatment (Fig 3 in main text), α (B) β (C) and γ (D). Genes are ordered according to their reported biological role, as erythroid-associated (red box), stemness (green box), myloid-associated (blue box) and endogenous genes in all subplots. Based on the expressed genes, the β subpopulation seems to be committed to the myeloid lineage while the γ subpopulation is committed to the erythroid lineage. The α subpopulation exhibits an indeterminacy with a bias towards the myeloid lineage. (E) PCA of all attractor cells (d0 and d6) as shown in the S4 Fig combined with the cells from the α (yellow), β (green), and γ (pink) subpopulations support the above described similarity to the untreated EML, the GM-CSF/IL-3 stimulated and the EPO-stimulated cells, respectively. (F) Coefficient of variation CV of expression levels of distinct genes is used as a cell-specific quantity to expose population dispersion and has no direct physical meaning; it was calculated for each cell from the expression levels across all genes for each subpopulation. Histograms represent the number of cells at different level of the CV measure and show that cells in α subpopulation have higher spread of cellular CV values. (JPG) [file pbio.2000640.s005.jpg]

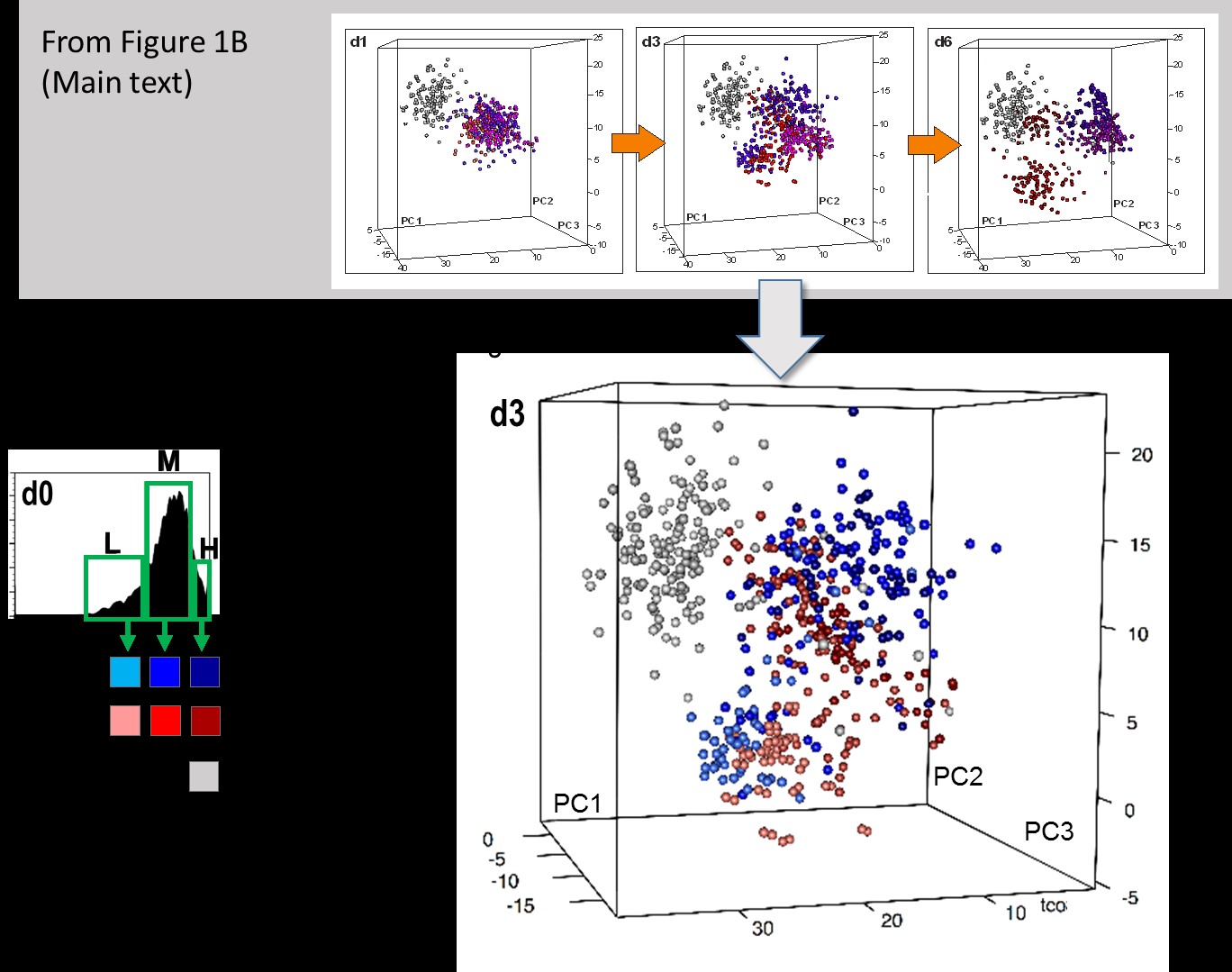

Supplement: S6 Fig — Colors of cells again (as in Fig 1) indicate treatment, but in addition, their provenience from the respective Sca-1 fraction in the progenitor population (d0). Rebellious cells are cells that shift toward the non-intended fate, i.e. EPO-treated cells moving towards the destination region of the differentiated myeloid cells in the top/right region and IL-3/GM-CSF-treated cells moving toward the prospective destination region of the differentiated erythroid cells in the bottom left region (see day 6 in top panel). To illustrate that the rebellious are recruited from the respectively primed states, the Sca1-fraction (LOW, MID, HIGH) in the progenitor population from which the cells originated was indicated by the color hue (see legend on left) for the day 3 stage: the darker, the higher was d0-Sca1 expression. Note that the perspective is slightly shifted from that of Fig 1 to show that there is no clean separation at d3 into two disjoint cluster. As previously noted (see MAIN TEXT), the Sca1HIGH cells are primed towards the myeloid cells, whereas Sca1LOW cells are primed towards the erythroid cells. Note that although at this time point at d3 the cloud spreads and begins to split, and there is no separation between IL-3/GM-CSF and EPO treated cells. However, rebellious cells tend to originate from the Sca1-fraction for which they are primed. For instance, Sca1LOW cells in the progenitor population which are known to be primed towards erythroid fate [4], are the source of the rebellious cells which despite treatment with IL-3/GM-CSF move towards the erythroid direction at d3 (light blue cells). Thus, priming determines fate more than instruction by the external signal. (JPG) [file pbio.2000640.s006.jpg]

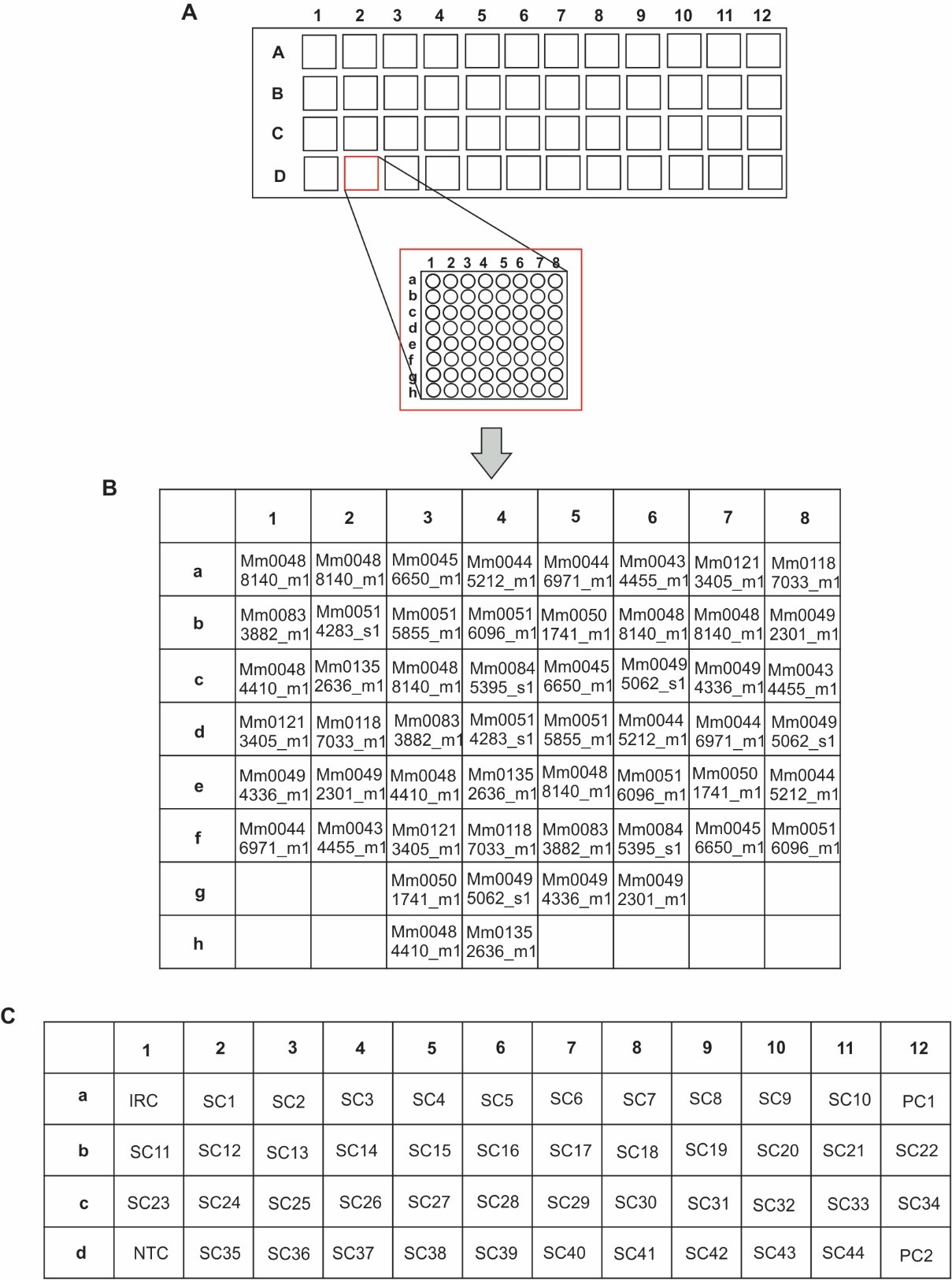

Supplement: S7 Fig — (A) Each OpenArray (Applied Biosystems) is the size of a microscope slide. It holds 48 groups (subarrays, red rectangular) of 64 holes of 33 nl volume in which one PCR reaction occurs. A hydrophilic layer is at the interior surface of each hole and a hydrophobic layer is at the exterior surface of the plate allowing for filling the hole by surface tension. In total, each array carries 3072 qPCR reactions. (B) Specific PCR primers are pre-immobilized in individual holes (by manufacturer, for customized assay patterns) and released by heat in the first cycle. (C) An example of the distribution of single-cell samples (SC) along with NTC (no template water control), IRC (inter-run calibrator) and 100-cell control (PC) samples on an OpenArray chip. (JPG) [file pbio.2000640.s007.jpg]

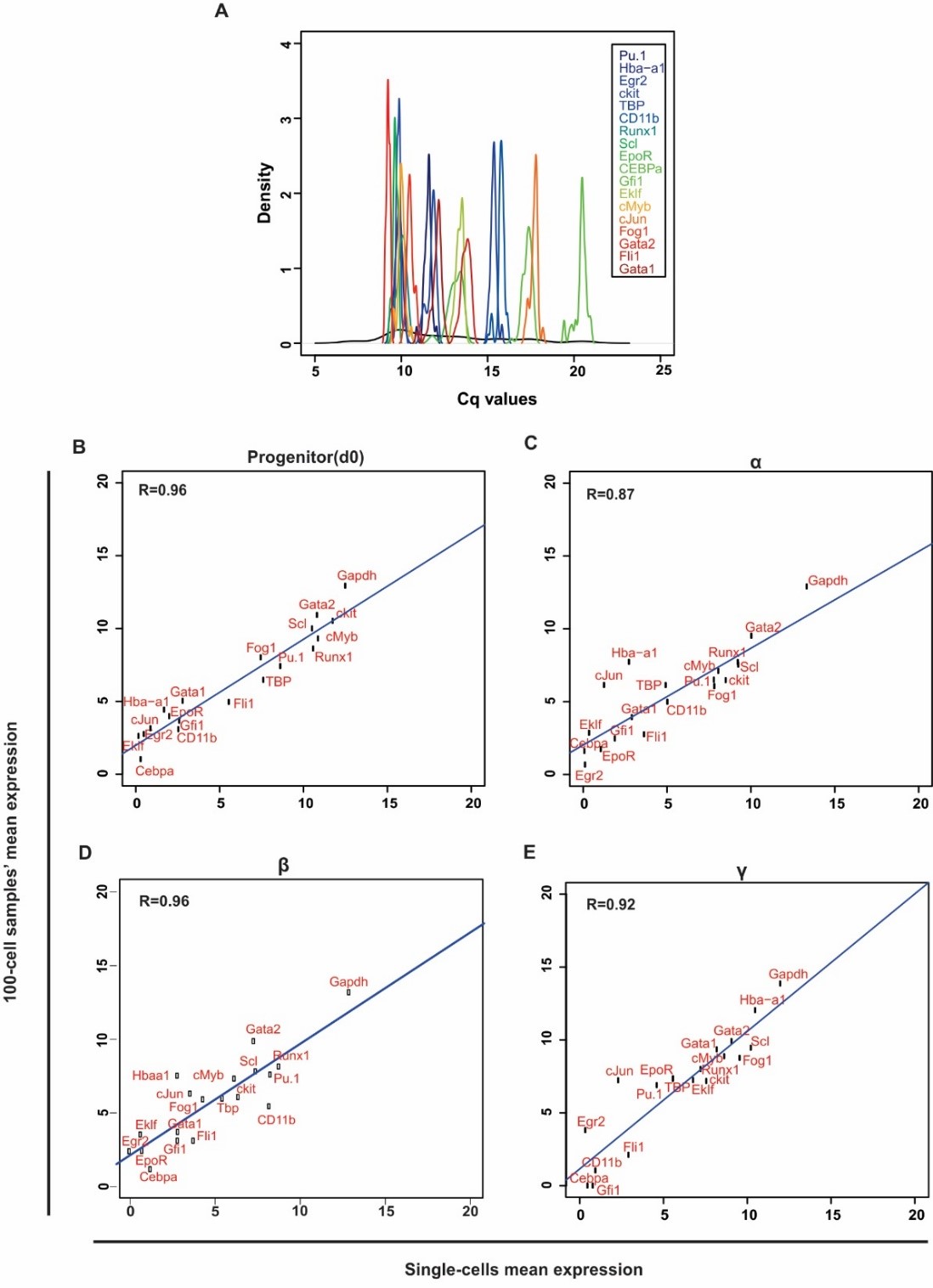

Supplement: S8 Fig — (A) Inter-chip variability is evaluated using inter-run calibrator (IRC) sample. Each curve represents the distribution of Cq values of each gene across all OpenArray chips. The flat black curve represents the distribution of all genes across all chips. The inter-gene differences are up to 2 orders of magnitude larger than the inter-chip variability of the same gene. The inter-run calibrator was a 10-fold diluted sample of 18 cycles pre-amplified cDNA of 10 ng isolated RNA from EML progenitor cell population. (B-D). Correlation between gene expression in an ensemble of 48 individual cells and 6 replicates of 100-cell pools is plotted. Cells used were from subpopulations α, β and γ (subplots b-d) as presented in Fig 3 and 19 genes as listed in S3 Table were measured in triplicate in all single cells and bulk (100-cell) samples from each subpopulation. Mean expression for each gene was calculated across all single cell or pool samples. Note that the scaled mean expression for 100-cells pool was plotted against mean expression for single-cells. In all cases a high correlation between single-cell data and bulk data with correlation coefficient of > 0.86 was observed. (JPG) [file pbio.2000640.s008.jpg]

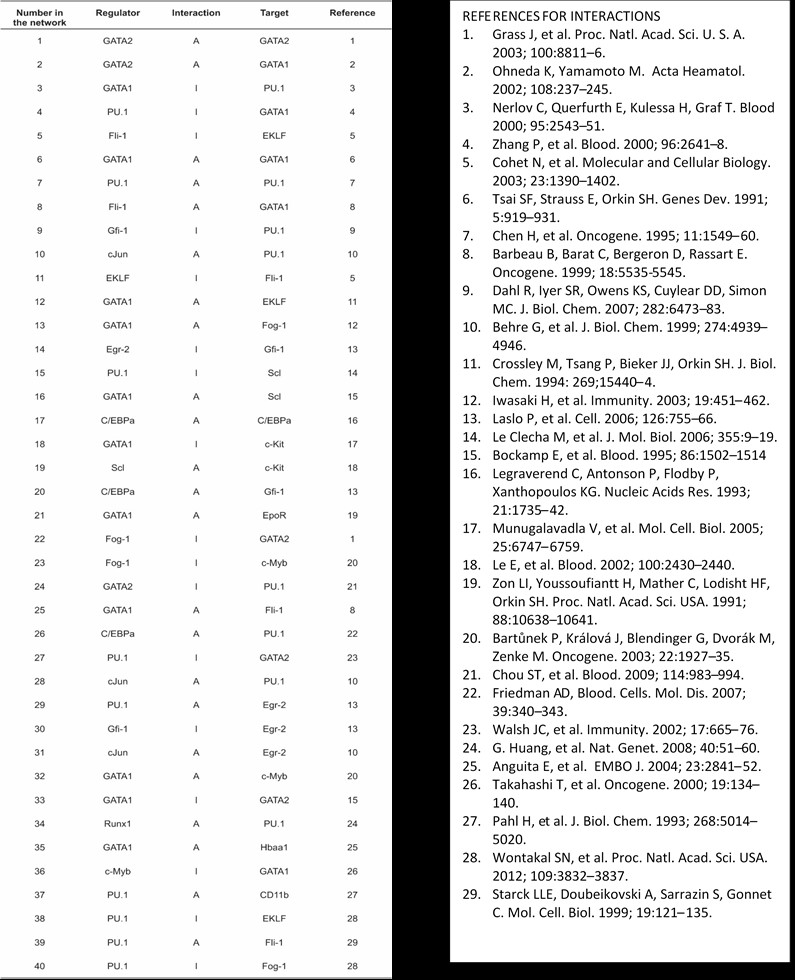

Supplement: S1 Table — Table of the regulatory interactions (either activating (A) or inhibiting (I)) between the genes. For each interaction, the literature is referenced (numbered list in the right panel). All interactions have been reported in for murine hematopoiesis. (JPG) [file pbio.2000640.s009.jpg]

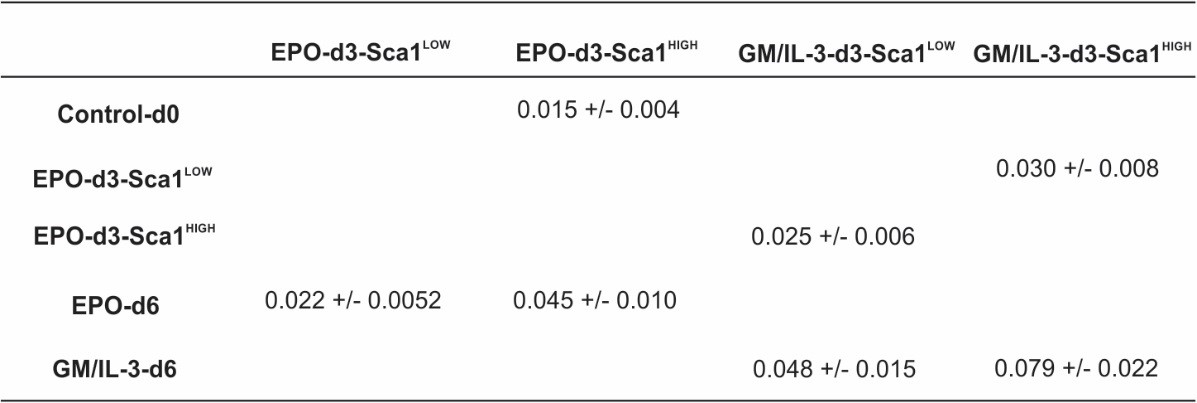

Supplement: S2 Table — Pair-wise dissimilarity between expression profiles (samples) was calculated based on the normalized gene expression levels for 6297 filtered genes (see Material and Methods) with 1–R where R is the Pearson’s correlation coefficient which ranges from 0 to 1, meaning that 0 correspond to highest similarity and 1 to most different expression. Bootstrapping was performed by randomly selecting 30% of the genes in any sample to calculate the pair-wise dissimilarity metric and repeating the procedure 10,000 times to generate the reported standard deviations. (JPG) [file pbio.2000640.s010.jpg]

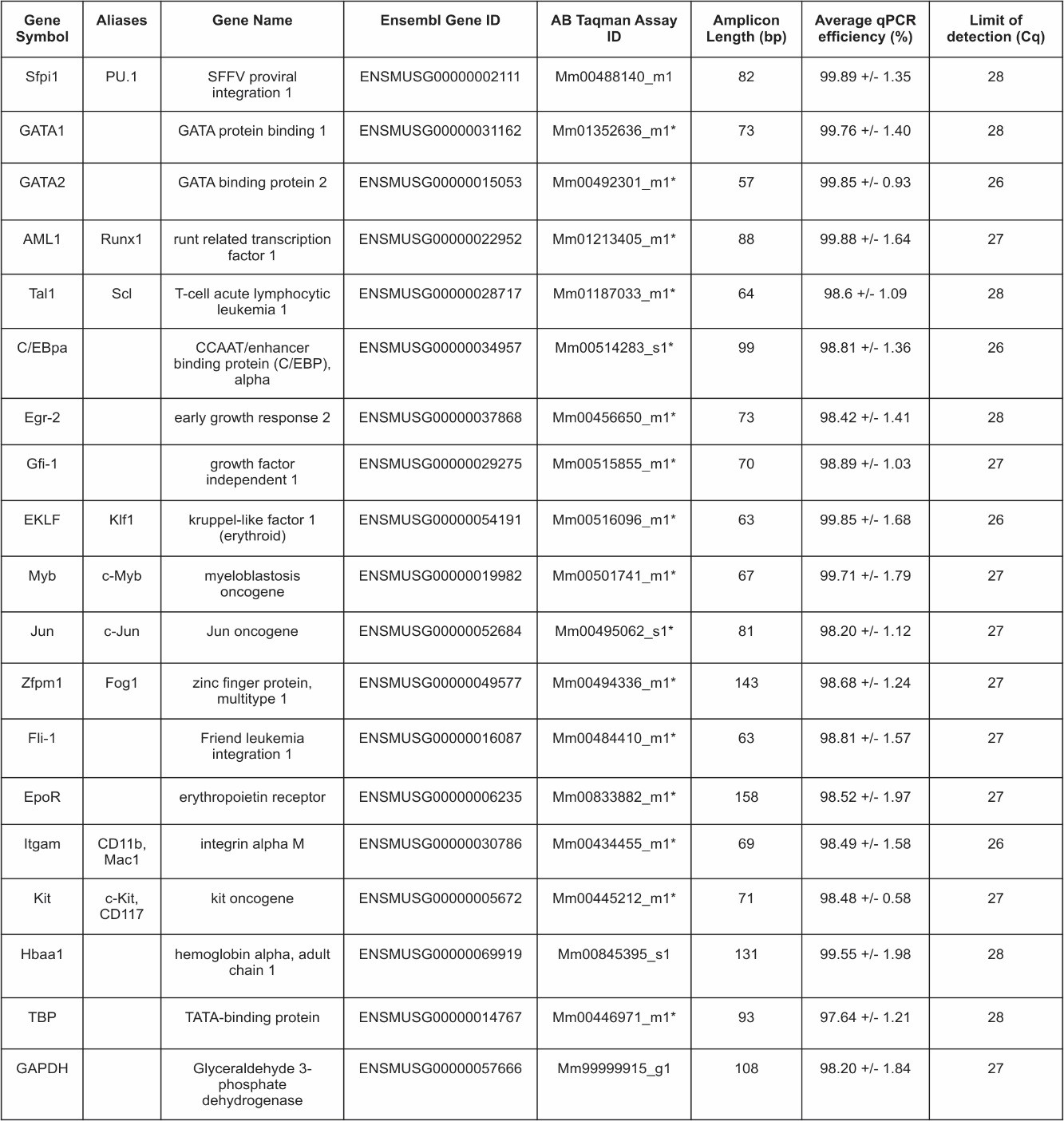

Supplement: S3 Table — Table lists all primer pairs and relevant information including IDs and amplicon length. All assays were inventoried. Identical PCR primers were used in the pre-amplification step and the subsequent singleplex qPCR step. In addition, the amplification efficiency and limit of detection (LOD) of the qPCR assays are given. To evaluate efficiency and LOD, a 1:2 serial dilution was prepared from 18 cycles pre-amplified product from 10 ng RNA purified from EML progenitor cell population. Amplification efficiency was calculated according to: [10^(1/-S)-1] × 100%. The slope was obtained by linear regression of the standards curve. Efficiency was determined as average of two biological replicates with 6 qPCR technical replicates each. The Cq value for the LOD is defined as the most diluted sample that results in positive amplification for 5 out of 6 replicates. (JPG) [file pbio.2000640.s011.jpg]
