# Supplementary material for: Cell Fate Decision as High-Dimensional Critical State Transition
Source: PLoS Biol. 2016 Dec 27;14(12):e2000640. doi: 10.1371/journal.pbio.2000640 (PMC5189937; doi:10.1371/journal.pbio.2000640)
Supplement: S1 Appendix — (PDF) [file pbio.2000640.s015.pdf]

S1 Appendix

A. Data Analysis

This S1 Appendix (Section A of Appendix) contains details on data analysis of the new results presented in the main text. For analysis of published data as discussed in the text to support robustness of the index I_C see S3 Appendix. Data analysis of single-cell qPCR transcript expression was performed, if not stated otherwise, using R version 2.15.0 with several packages listed below.

A.1. Data pre-processing overview

In brief, pre-processing of single-cell RT-qPCR data involved multiple steps including data arrangement, false positives elimination, missing and off scale data corrections, calculation of median for 3 qPCR technical replicates, calculation of transcripts copy numbers relative to the background, and linear transformation of relative quantities of the transcripts.

To allow for maximal comparability and compensation for potential readout variability, the material of individual cells originating from distinct subpopulations regarding treatments and time points was assigned in a systematic way to the spots on the BioTrove OpenArray (Life Technologies) plates to avoid local clustering. On each plate, additional negative template control (NTC), inter-run calibrator (IRC) controls and sorted 100-pooled cell samples were loaded (S8 Fig). The resulting single-cell qPCR data (Cq values) were exported from the OpenArray qPCR analysis software as csv files and subsequently organized in Microsoft Excel spreadsheets with single-cell samples in rows and genes in columns. The amplification curves of all PCR reactions were first manually analyzed to remove anomalous curves and false positives. Next, the values of the three technical replicates were inspected and when not available replaced by "NA" (Not Available). To determine the offset of the data, we found experimentally that the Cq-value (cutoff of amplification signal) was not reliable. We therefore determined the limit of detection (LOD) for all investigated 19 genes as the lowest amount of target DNA that can be accurately and reproducibly quantified (S3 Table). In the final pre-processing step, the median of each gene in single-cell samples was calculated for the three technical replicates (S1 Table) and only consistent Cq values between 16-28 estimated from 100-cell sample data were used for the statistical analysis. Higher or lower Cq value were only accepted based on examination of the corresponding amplification curves where least 2 out of 3 technical replicates must exhibit consistent values.

A.2. Data reassembly and expression conversion

The pre-processed data (Biomark, Material and Methods) was further processed with R. Based on the unique labeling of the samples, first single-cells, controls and 100-cell data were separated and, when applicable, subsequently sorted with respect to the Sca1 (the progenitor marker)-based FACS classifier, treatment and time point. To convert Cq values into expression values, the measured Cq values were subtracted from the experimentally determined LODs resulting in Log_2Expression (LOD-$Cq_{GOI=Gene\ of\ Interest}$). As a result, this calculation leads to a log expression where negative entities were set to zero, as suggested by the Fluidigm protocol [1]. To test if this procedure can induced artifacts in the correlation analysis, the negative entities were also replaced by small (< 5% of gene average) uniformly distributed random numbers. Neither this alternative treatment of negative values has shown any noticeable difference in the subsequent correlation analysis nor mutual information estimates. Therefore, negative values were set to zero for subsequent analysis. The processed data were then reassembled in various ways for the following analysis in accordance to the specific investigations.

A.3. Quality control

To analyze inter-plate variability and any potential correlation induced by technical bias, the IRC controls of each gene were compared for all plates. The investigation exhibited no significant correlations within a plate and the inter-gene differences were up to 2 orders of magnitude larger than the inter-plate variability of the same gene (S 8A Fig). As a more rigorous and quantitative control for accuracy of the single-cell analysis, the measured expressions of sorted 100-cell samples were compared to the average expression of tens of single cells. This approach compares the experimental average scenario based on a potentially heterogeneous mixture of 100-cell samples to an analytic average of tens of individually quantified single cells. The comparisons agreed very well for all different treatments, and the corresponding Pearson's correlation coefficients were always larger than $R^2 > 0.82$ (minimum value of coefficient) with typical values being larger than $R^2 > 0.9$ (average value of coefficient) (S 8B Fig). In summary, the quality control confirmed the high reproducibility and sensitivity of the single cell RT-qPCR platform, thereby ensuring that the expression data were of high quality without experimentally induced bias. In particular, the consistent IRC controls and the agreement between the 100-cell and single-cell averages demonstrated that (in a large majority of cases) a not-expressed gene within a single-cell is a real biological phenomenon and not caused by experimental detection issues.

A.4. Correlation analysis

To analyze inter- and intra-population relations between cells and genes (Fig 2 and 3), correlations were calculated with the R-function *rcorr* of the *Hmisc* package along with asymptotic P-values based on the sample size. For visualization and validation purposes, additional correlation investigations were performed using the *pair.panels* R-function of the *psych* package for scatterplot analysis. Final correlation plots were generated with the *corrgram* function.

For the temporal analysis of gene-gene correlation behaviour during a differentiation process, only significant gene correlation with p-values < 0.01 were considered. The analysis was performed for each time point and treatment. The analysis was applied to both the average number of significant correlations and the average absolute value of significant correlations. The qualitative agreement of these analogous approaches indicates the stability of this analysis method.

A.5. Principal Component Analysis for individual cells as statistical variables

To visualize the differentiation process in the cell state space, principal component analysis (PCA) was performed on the processed single-cell gene expression data using the R-function "princomp". A cell's state is defined by a gene expression vector, which is composed of the set of the 19 gene expression values as its components, and thus would correspond to a point in the 19-dimensional state space. In brief, the PCA identifies the directions in this space along which the variation of expression is maximal [2]. The resulting directions called principal components (PCs) are linear combinations of genes where more informative genes contribute with larger weights to the linear combination. PCA leads to new orthogonal reference system where the first PCs (i.e. dimensions) explain the majority of observed variation and therefore allows for mapping the data from original high-dimensional space (where each gene defines a dimension) onto a new dimension-reduced space spanned by fewer variables (PCs). In this way, PCA can help in the visualization and interpretation of the high dimensional data.

Since we are interested here in the transition of cells from the progenitor attractor state into the differentiated states, we adapted the classical PCA approach to temporal time series. Therefore, we determined the PCs and corresponding weights of genes for the subset of data that contains only cells in the assumed attractor states namely cells on day 0 and on day 6 treated with EPO and GM-CSF/IL-3, respectively. This approach leads to a maximal separation of the assumed attractor states and the first three PC explain close to 80% of the variance (S4 Fig). Interestingly, by analyzing the resulting gene weights for the first three PCs revealed that this unbiased approach associates stemness genes with the progenitor cells, typical erythroid genes with the EPO treated cells and

myeloid related genes with the GM-CSF/IL-3 treatment (S4 Fig). Based on this consistent result, we then used the resulted attractor state space for the transition analysis by transforming cells of day 1 and day 3 into the same reference system. Therefore, the weights w_m^i of gene m to PC i determined by PCA of the attractor data subset were multiplied with the expression value x_m^k of individual cell k and gene m from time point day 1 and 3. This leads to corresponding 3 dimensional coordinates $\mathbf{r}^k = (r_1^k, r_2^k, r_3^k)$ with $r_i = \sum_m w_m^i\, x_m^k$ for each cell k within the 3 dimensional attractor state space defined by the PCs of the attractor data set. Within the resulting Euclidian state space we can calculate consistent distances between attractors and corresponding variances that can be used for the transition analysis (Fig 2).

A.6. Coefficient of Variation (CV) analysis

To analyze the population heterogeneity in terms of gene expression variability we computed the Coefficient of Variation (CV, i.e., mean-normalized standard deviation) of the all gene expression values (across all genes) for each cell's state vector. This represents an aggregated cell-state variable can serve as a quantity to assess the increase in cell-cell variability during a critical transition (see S2 Appendix).

A.7. Comparison between qPCR and RNA-seq for single cell transcript analysis

The current gold standard method for whole-transcriptome in individual cell is widely assumed to be single-cell RNA-seq. However, this technology still suffers from technical limitations that makes it unsuited for the analysis of critical transitions that we seek because it demands higher accuracy and precision than descriptive studies. Moreover, as shown further below (Section C, and explained in the main text) there is no need, neither for statistics nor for formal reasons, to use the large number of transcripts the entire transcriptome provided by RNA-seq.

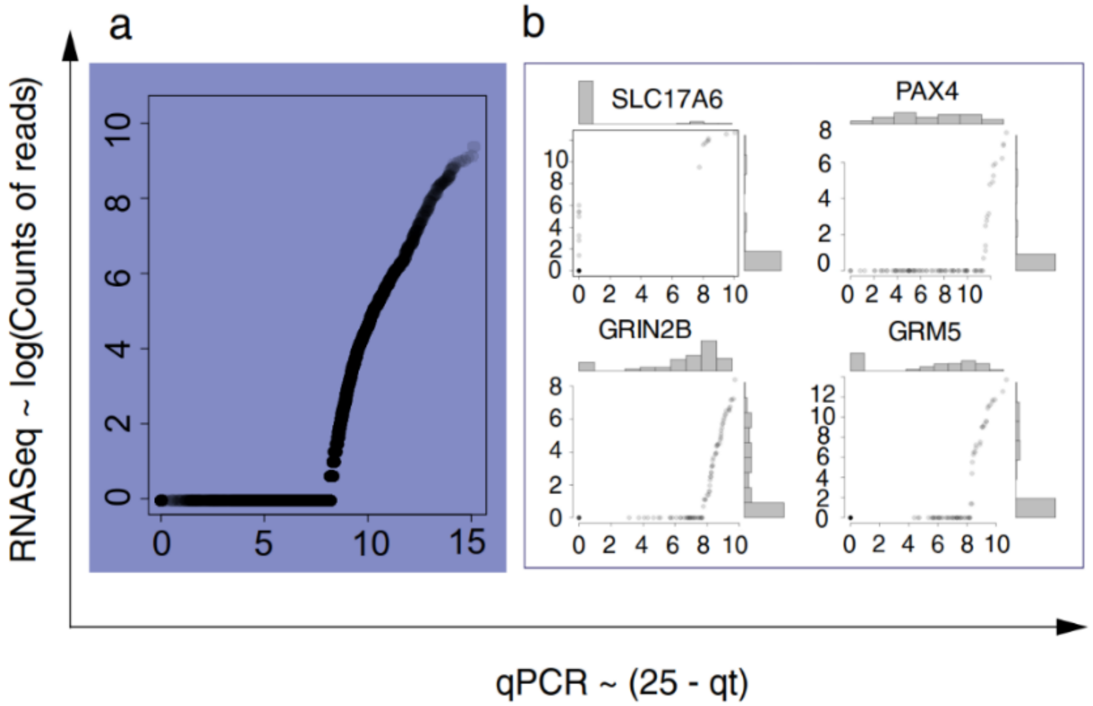

Figure A-1. Comparison of scRNA-seq and sc-qPCR in the analysis of 48 genes. Two sets of 96 Individual cells were separately analysed using either method for the same set of 48 genes. X-axis is the expression value in qPCR and Y-axis is the expression value in RNA-seq. Each dot is a cell. For each gene, cells were ranked within each method to allow for direct comparison and values of cells of the same rank in each method graphed as dot plot based in the standard units for expression level in the respective method. ***a***. Compilation of data of 46 (of the 48) genes in which qPCR outperformed scRNA-seq. Axis are in the units used routinely in the respective technologies (for qPCR, background was set at qt=25 cycles.) ***b.*** Top-left panel: the case of genes for w hich scRNA-seq performed better; other panels: three random samples in which qPCR was more sensitive. Histograms of frequencies of cells in the respective ranks shown along the two axes.

The main limitation of RNA-seq is its low sensitive compared to qPCR when used for single-cell transcript measurement. Published studies suggest that only ~10%, at best 40% of transcripts of a cell are captured –most likely due to the low efficiency of single-cell handling, lysis and reverse transcription (RT) [3-5]. This explains the vast blocks of 'zero'-entries in the data matrix which can cause problems in integrated analyses of entire transcriptomes. While qPCR suffers from part of the sane problems (sharing the steps up to RT) it has much less zero-entries due to higher sensitivity in detecting low-level transcripts. We performed a direct comparison by measuring 48 transcripts in two sets of 100 iPS cells using either sc-RNAseq (transcriptome-wide cDNA library preparation using SMART-seq [4] in Fluidigm C1 followed by Illumina NextSeq for sequencing) or single-cell qPCR (FACS, pooled pre-amplification and qPCR in Biomark using Fluidigm's VILO protocols). The **Fig A-1** above shows that for 46 of the 48 genes examined, qPCR offered a substantially broader dynamic range that is approximately twice that of sc-RNA-seq, covering low level expression that is below detection in sc-RNA-seq (at 3-6M reads per cell). In one case qPCR did not perform and in another case (shown as subpanels in the Figure above) it exhibited saturation at high level expression where RNA-seq was still quantitative. Thus, given that the correlation depends upon the 'range effect' (see S2 Appendix, **B.2.**) and the dynamic range of transcript levels in scRNA-seq is narrow, it is clear that the latter is an inferior technology compared to sc-qPCR for our purpose.

One reason for the higher sensitivity of qPCR for low-abundance transcripts may lie in the targeted preamplification that compensates for the low efficiency of the RT reaction which is further accentuated by the template-switch protocol used in current scRNA-seq library prep. In fact, internal tests of RNA-seq that employ a *targeted* amplification as used in qPCR achieved similar levels of sensitivity (unpublished observations).

A.8. Statistical robustness of the index I_C (see also special analysis section ***C*** on other data)

The following is a pedagogical dissection of one subset of the single-cell transcript data to demonstrate the statistics-based argument behind index I_C and its robustness, as explained in Section **B.2**. of this Suppl. Information (Appendix S2).

We focus on the time point when the cell population is in the attractor (=d0, untreated) to explain the data structure. This is an equilibrium state and the behavior of cells is straightforward: each cell's gene expression is only exposed to random fluctuations around an expected steady-state gene expression level for each gene.

In this state we expect that the numerator of I_C (which summarizes the GENE-GENE correlations) is low because each gene fluctuates symmetrically around its characteristic "set point" due to gene expression noise, and thus it is uncorrelated to any other gene. As a consequence, the GENE-GENE *correlations* (positive and negative) have low absolute values [6]. In contrary, since each gene has a *typical* (state-defining) *average* expression value across all the cells, we do expect that, by the action of this BETWEEN-GENES *variability* in expression, the various *cells* will be very much (and only positively) correlated. The above condition corresponds to the elementary definition of the strength of correlation in linear models as the ratio of the variability between traits (e.g. gene expression values) and their variability within a group (e.g., of cells).

It is then evident that the central influence on the index I_C is NOT the NUMBER OF GENES taken into consideration but the fact that the differences of *distinct* genes in their values averaged over all cells (variability BETWEEN genes) are higher than their internal variance, i.e. the *same* gene across all N cells (variability WITHIN genes). This is in fact the case, as the descriptive statistics over the 17 genes at equilibrium (for t = day 0) shows:

GENES	#CELLS	MEAN	VARIANCE
Gata1	150	5.7497	9.3804
EpoR	150	1.807	10.629
Eklf	150	3.2716	15.177
Fog1	150	7.9945	14.469
Hba-a1	150	5.9451	8.0083
Scl	150	11.813	2.2295
Fli1	150	5.5103	18.552
Runx1	150	10.332	5.866
Gata2	150	12.34	3.6827
cMyb	150	11.034	5.6037
ckit	150	11.224	5.826
sfpi1	150	8.7699	7.0705
CEBPa	150	0.2276	1.5689
Gfi1	150	1.6146	9.5376
cJun	150	0.6756	5.0336
CD11b	150	1.7553	10.962
Egr2	150	1.3159	8.3395
		**Variance:**	**Average:**
		**18.95**	**3.3491**

The BETWEEN-GENES variance for all genes (=variance of the '*Mean'* column in the above table –the mean of a gene across all cells) is **18.95**; on the other hand, the "WITHIN-GENE" variance averaged over all genes (=average of the '*Variance*' column –the variance of a given gene across all cells) is **8.35.** The ratio of variance 'BETWEEN' and variance 'WITHIN' the GENES, 18.95/8.35, is sufficiently high (far away form 1.0) and is the basis of the *high* "CELL-CELL" *correlation* (building on the *high* BETWEEN-GENES variance) and the *low "*GENE-GENE" *correlation* (building on the *low* WITHIN-GENES variance) in the attractor state that serves as a starting point of the index I_C.

The statistical significance can be understood as follows: Given that a correlation depends on the *range* of explored variation and few genes spanning a high variation of expression give rise to higher correlation than many genes with very similar expression values, it is evident from the above that a range spanning a huge expression space going from **0.23** to **12.3** (in qt = log2 units, see Table above) affords optimal condition for estimating the profile of cell-cell correlations. This well-known *strict dependence of correlation from variance* is the '**range (restriction) effect**' and is explained in more detail in [7] (see paragraph 'basic pillars of linear correlation') and used in [8].

The data in the table above tells us that these 17 genes, not because they are 17 but because they have a very high BETWEEN-GENES *variability* with respect to WITHIN-GENE *variability* provide a solid basis for the analysis of correlations and their changes. (The reason for these changes is formally demonstrated in Supplementary text S2.). Thus, from this elementary consideration alone there is *no need* to analyze more genes because the 17 genes covered sufficient variability. On the other hand, for random noise to produce the results it would have to meet the *two orthogonal* requirements at once: increasing BETWEEN-GENES variability (in each cell) while at the same time decreasing the WITHIN-GENE variability (across all the cells).

We can show now that this leads to statistically robust **correlation coefficients** for I_C. As expected from the above discussion and the main text, in the stable attractor (day = 0) the average CELLS-CELL *correlation* (correlation coefficients for all pairs of cells, then averaged) was maximal with a value of r = 0.70, while the average GENE-GENE correlation was minimal, at r = 0.10. To get a 'probabilistic' appreciation of the ratio as defined by I_C we used the data matrix $\mathbf{X}(T)$ of the same time point and generated 30 randomly shuffled versions of it. We shuffled the internal order of the *columns* (genes), an operation that maintained both mean and standard deviation of GENES but destroyed any CELL-CELL *correlation* not dependent from the pure effect of BETWEEN GENES *variability*. The maintaining of the average value of expression of each gene (column) is the only 'biologically motivated' constraint and permissive given the ubiquitously observed near-unity correlation between genome-wide expression profiles of independent samples of the *same* kind of tissue (but see discussion the example in Section C. for *heterogeneous* tumor

cells). The 30 different simulations were evaluated for both average GENES-GENE *correlation* and CELL-CELLS *correlation*, across the 30 samples, revealed very robust results:

	Average	Std. Dev.	Std. Err.
CELL-CELL Correlation:	0.691	0.004	0.0008
GENE-GENE Correlation:	0.063 0.	0016	0.0003

This implies that at in the attractor state we have an average value of the index I_C equal to 0.063/0.691 = *0.09* –similar to the one obtained for the entire set of data at day 0 in Fig. 2 of main text. In the results we found an increase of I_C to *0.4* (Fig. 2 of the manuscript) and thus a signature of a critical transition; this value is far outside the Standard Error for I_C and corresponds to a $p < 10^{-10}$. (This is analysis is equivalent to the bootstrap analysis performed in the manuscript.)

References for S1 Appendix (A)

1. Fluidigm. Application Guidance: Single-Cell Data Analysis-RevA1. 2012; 1-40.
2. Bergkvist A, Ruskanova V, Sindelka R, Garda JAM, Sjogreen B, Lindhl D, et al. Gene expression profiling-Clusters of possibilities. Methods. 2010; 50:323–35.
3. Grun D, Kester L, van Oudenaarden A. Validation of noise models for single-cell transcriptomics. Nature methods. 2014; 11(6):637-40.
4. Marinov GK, Williams BA, McCue K, Schroth GP, Gertz J, Myers RM, et al. From single-cell to cell-pool transcriptomes: stochasticity in gene expression and RNA splicing. Genome Res. 2014; 24(3):496-510.
5. Wu AR, Neff NF, Kalisky T, Dalerba P, Treutlein B, Rothenberg ME, et al. Quantitative assessment of single-cell RNA-sequencing methods. Nature methods. 2014; 11(1):41-6.
6. Giuliani A. Statistical mechanics of gene expression networks: Increasing connectivity as a response to stressful condition. Adv Systems Biol. 2014; 3(1):3-6.
7. Giuliani A, Zbilut JP, Conti F, Manetti C, Miccheli A. Invariant features of metabolic networks: a data analysis application on scaling properties of biochemical pathways. Physica A. 2004; 337(1-2):157-70.
8. Gorban AN, Smirnova EV, Tyukina T. (2010). "Correlations, risk and crisis: From physiology to finance." Physica A: Statistical Mechanics and its Applications. 389(16): 3193-3217s
