# Supplementary material for: Cell Fate Decision as High-Dimensional Critical State Transition
Source: PLoS Biol. 2016 Dec 27;14(12):e2000640. doi: 10.1371/journal.pbio.2000640 (PMC5189937; doi:10.1371/journal.pbio.2000640)
Supplement: S2 Appendix — (PDF) [file pbio.2000640.s016.pdf]

S2 Appendix

B. Derivations and Explanation

*This second Appendix explains the idea behind critical state transitions in a statistical ensemble of high-dimensional dynamical systems. Section **B1** summarizes the phenomenology and basic formalism underlying the index I_C; section **B2** presents a plausible argumentation to explain the index I_C based on elementary statistics; and section **B3** is a mathematical derivation of the I_C from the perspective of dynamical systems theory. Experimental biologists should at least read B1, the more quantitatively inclined, also B2, while B3 is for theorists. The thinking is still developing and feedback, with corrections or extensions are welcome.*

B.1. Phenomenological signature of critical state transition for a high-dimensional system

We consider here the state $\boldsymbol{S}^k$ of a cell k = 1, 2, ..n as generated by a dynamical system, the gene regulatory network (GRN) encompassing the m=17 genes, and that hence the *observable* state $\boldsymbol{S}^k$ of a cell k, $\boldsymbol{S}^k$ =[$x_1^k, x_2^k, .. x_m^k$], maps to a point in the m-dimensional state space spanned by the m=17genes studied, as discussed in conjunction with Fig 1. Herein x_i^k (i=1,2, ..m=17), referred to as "gene expression value" throughout, is the cellular abundance of transcript i in Log_2Expression units (see **B.2**), as measured by qPCR for all the m=17 genes in cell k.

However, unlike in typical dynamical systems models, we deal with an *ensemble* of n systems, that is, although cells are nominally identical, they differ at any moment in time t in their gene expression profiles (m state variables) due to gene expression noise (stochastic fluctuation in x at various time scales not further considered here). With regard to experimental reality, we can measure the statistical distribution of $\boldsymbol{S}^k$ as population "snapshot" at single-cell resolution but we cannot monitor the very same ensemble member (cell) over time.

While the deterministic dynamics of a cell is still governed by an ODE system $\dot{\boldsymbol{x}}(t) = \boldsymbol{F}(\boldsymbol{x}(t))$ that captures the regulatory interactions of the GRN through which x_i influence each other according to the rule defined by the GRN, we consider the form of $\boldsymbol{F}(\boldsymbol{x})$ unknown and currently unknowable, and is not further discussed. By contrast, we are concerned with the *observable* $\boldsymbol{S}^k(t)$ = $\boldsymbol{x}^k(t)$ for the cell k where t is a measurement point of a given (sub)population in a particular culture condition treatment at a given time.

As the multipotent progenitor (untreated EML) cells commit to a lineage, the "quasi-potential landscape" U(x) [1], which manifests the regulatory constraints imposed by the GRN on the collective change of the gene expression values in each cell, must change such that the attractor of the progenitor state is destabilized and undergoes a critical state transition (possibly, a pitch-fork type bifurcation [2]. In this process cells exit the destabilized progenitor state, which at some point abruptly vanishes, and they gain access to (new or newly accessible) attractor states that establish the stable gene expression patterns of the committed erythroid and the myeloid cells, respectively. Without knowledge of the systems specification, $\boldsymbol{F}(\boldsymbol{x}(t))$, that govern the dynamics of $\boldsymbol{S}$, hence, without knowledge of any bifurcation parameter(s) P, how can one detect a critical state transition by measuring $\boldsymbol{S}^k$ for n = 1000s of cells k?

In the spirit of phenomenological (mechanism-free) analysis of dynamical systems [3], and, inspired by the concepts of empirical study of critical state transitions in low-dimensional systems [4, 5] we postulate, in analogy to the "early warning signals" which characterize the approach to a critical transition point, the following for a higher-dimensional system, namely, that:

> *the index $I_c(t)$ at sampling time point t increases to a maximum as the cell population approaches and undergoes the critical state transition (~bifurcation).*

Herein, the index $I_c(t)$ is defined as follows:

$$I_C(t) = \frac{\langle |R(g_i,g_j)| \rangle}{\langle R(S^k, S^l) \rangle} \quad (1)$$

where R is Pearson's correlation coefficient between the two observed cell state vectors S^k and S^l or between the two "gene" vectors g_i and g_j respectively, from the gene expression data matrix of the state of a "cell ensemble" $\boldsymbol{X}(t)$ (a (sub)population of cells in a given treatment/condition at a given time point t):

$$\boldsymbol{X}(t) = \begin{bmatrix} x_1^1 & \cdots & x_m^1 \\ \vdots & \ddots & \vdots \\ x_1^n & \cdots & x_m^n \end{bmatrix} \quad (2)$$

$\boldsymbol{X}(t)$ thus represents the data of a "measurement point" in traditional whole-cell population experiments but here we have access to a finer-grained layer of information due to single-cell measurement: each row vector represents a cell in its state k within the cell-ensemble $\boldsymbol{X}(t)$ of n cells in m-dimensional gene state space: $\boldsymbol{S}^k = [x_1^k, x_2^k, .. x_m^k]$ and each column vector represents a gene i 's expression values across the n cells of said cell-ensemble $\boldsymbol{X}(t)$: $\boldsymbol{g}_i = [x_i^1, x_i^2, .. x_i^n]$. The brackets <...> in (1) denote the average of all the correlation coefficients R between all the pairs of state vectors S or the gene vectors g in the matrix $\boldsymbol{X}(t)$, respectively.

Below we provide two explanations for our postulate on the meaning of $I_c(T)$: one based on linear approximation (section **B.2**), considering the Pearson's correlation coefficient and variability of gene expression values (see the **Fig B-1** below), without usage of dynamical systems concepts, and the other based on non-linear dynamical systems theory (**B.3**.).

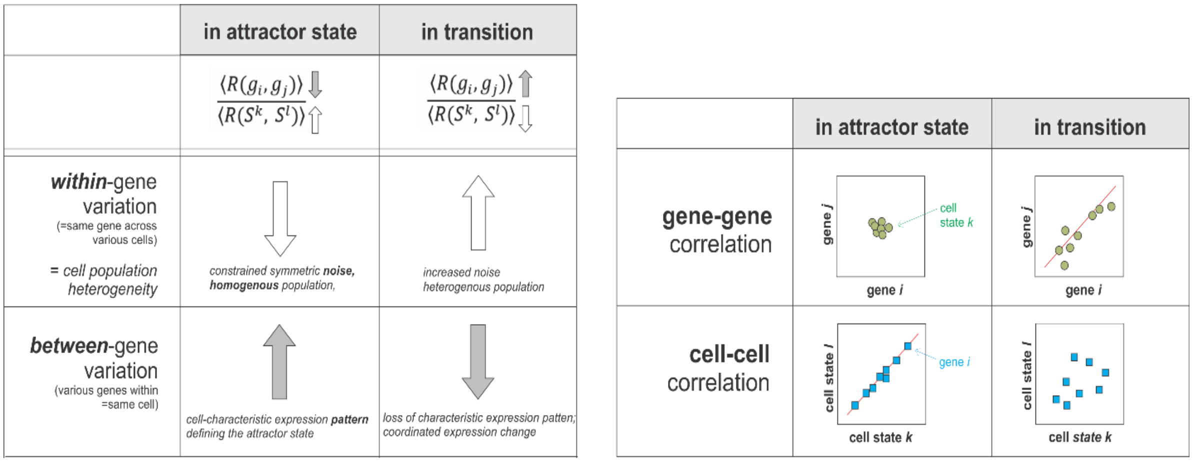

Figure B-1. Schematic explanation of the relationship between 'within-gene' and 'between-gene' variation and between 'gene-gene' and 'cell-cell' correlation – in the context of whether the cell populations ins in an attractor state of in transition. Large arrows indicate increase of decrease of the respective quantities.

B.2. Plausibility argument for $I_C(t)$ as indicator of state transition based on linear statistics

A first (intuitive) way to understand why a local/transient increase of $I_C(t)$ in which the denominator and numerator contain the Pearson coefficients computed from the same but transposed data matrix, marks a critical transition rests in the interpretation of Pearson's correlation coefficient. Here we need to first consider the different nature of variability of expression values. From a gene-centric point of view we distinguish between, on the one hand, the variability of gene expression (x) across *distinct* genes *within a given cell* ("***between***-gene variability") and on the

other hand, the variability of a *given gene* across *distinct cells* ("***within***-gene variability"). Note that the presence of only positive correlations allows us to use direct Pearson metrics.

Generally speaking, the Pearson's correlation coefficient builds upon the intuitive idea that the degree of association (coupling) of two *statistical variables* X and Y (for this consideration, X and Y stand for cell state vectors S^k and S^l, or alternatively, gene vector g_i and g_j) can be measured by the departure from the pure symmetry of the "bigger than mean" and "lower than mean" deviations of the XY-couple relative to the different *statistical units* (attributes of a given statistical variable). The Pearson correlation coefficient has the following formula:

$$R(X,Y) = \frac{X.Y}{|X||Y|} = \frac{\sum_{i=1}^{n}(x_i - \bar{x})\,(y_i - \bar{y})}{\sqrt{\sum_{i=1}^{n}(x_i - \bar{x})^2 \sum_{i=1}^{n}(y_i - \bar{y})^2}}$$

The formula exposes the leading role that deviations from the mean play in the determination of the correlation: for a significant correlation to be scored, X and Y variables must vary on a relatively large range (numerator of the R), otherwise the lack of a sufficient amount of variance will obscure the presence of any coupling between X and Y. This effect is well known to statisticians as the "*range restriction effect*" [6]. For small random fluctuations, the presence of a correlation is obscured by the symmetric character of noise-driven deviations from the mean, making the numerator going to zero.

Now, let us first consider the ***denominator*** $\langle R(S^k, S^l)\rangle$ in (1). It is easy to see why it is *high* for an ensemble in the attractor state and *decreases* towards the transition: In the stable attractor state each cell has the nominally the same distinct gene expression pattern except for random near-symmetrical deviations from it due to stochastic fluctuations – the population is relatively homogeneous ("compact cloud" of cells in state space, Fig 1B). The average of the Pearson correlation coefficients $\langle R(S^k, S^l)\rangle$ between all possible pairs of state vectors S in a cell ensemble $\boldsymbol{X}(t)$is a general measure of the 'homogeneity' of said cell ensemble, a measure we have used in Fig 2A, since $R(S^k, S^l)$ reflects the similarity of two cell states S^k and S^l. An alternative view is that we rely on a relatively high variation range corresponding to the *high* ***between***-gene variability, because each gene (as the statistical unit of a cell as a variable) has a *distinct characteristic* expression value that defines the attractor state and is similarly expressed in each cell. We thus expect *high* values of *cell-cell correlations*. But as the attractor "flattens" towards the bifurcation [2], that is, the attracting force decreases [1,7], the dispersion of cells increases which results in an overall decrease of cell-cell correlation (loss of the characteristic cell state *).

Second, why is the ***numerator*** $\langle |R(g_i, g_j)|\rangle$ *low* in the attractor and *increases* with its destabilization towards the transition? On contrary to the above, the gene-gene correlation R computed on the transposed space with gene couples as the statistical variables and the cell states as the statistical units, relies on ***within***-gene variability to produce a high value. This deviation from the mean of one gene across various cells is a manifestation of the symmetric random fluctuations of each gene around the characteristic expression value of the attractor state. Thus, in the attractor the *gene-gene coupling* is dominated by noise, which results in a strong range restriction effect [6] and consequently, $\langle |R(g_i, g_j)|\rangle$ is low. Therefore, in a homogenous situation within a stable attractor ("compact cloud" in gene expression state space), we expect a relatively *low gene-gene correlation* going hand-in-hand with the high cell-cell correlation. By contrast, as the attractor flattens during the transition, cells within the same population can (in a simplified view) be considered occupying nominally more different states. Biologically this is due to the *coordinated* (regulated) change in expression of *subsets* of genes, that is, a non-random shift of gene expression patterns that overrides the symmetric noise. Even if gene expression noise still abounds, *between*-gene variability of gene expression is not anymore solely due to random independent fluctuations of individual genes as it is around the attractor state (the case of homogenous cultures) but due to the onset of nominally distinct states inside the same culture. Thus a cell ensemble exposed to state

destabilization and transition is expected to be marked not only by a decrease in cell-cell correlation $\langle R(S^k, S^l)\rangle$ and but also a contemporary increase in gene-gene correlation $\langle R(g_i, g_j)\rangle$.

This explains the behavior of the index $I_C(T)$ as the cell population undergoes an abrupt state transition asynchronously. It is worth recalling, as discussed in section A8, that this behavior is a universal feature of systems under stress [See ref. 6-8 in S1 Appendix (A)].

B.3. Dynamical systems-based derivation of the index $I_C(t)$ as indicator for approaching bifurcation

Here we derive $I_C(t)$ (Eq. 1) from dynamical systems theory. We will do so separately for the numerator and denominator. First we show that overall "gene-gene correlation", taken as the average over all absolute correlation $\langle R(g_i, g_j)\rangle$ increases and reaches a maximum at the critical transition point. Second we will show that the transpose quantity on the same data matrix $\boldsymbol{X}(t)$, the overall "cell-cell correlation" $\langle R(S^k, S^l)\rangle$ reaches a minimum. The latter is intuitively plausible as manifestation of the dispersion of individual cells.

The central idea is that instead of having one deterministic dynamical system and its state *S*(*t*), single-cell analysis of the state transition of an entire population of nominal replicates of "deterministically identical" but due stochastic fluctuations distinct cells, provides data for a *statistical ensemble* that explores the state space around the deterministic trajectory and whose distribution will reflect the change of the vector field as a parameter *P* that is a characteristic of the dynamical system (e.g. strength of regulatory interactions between genes) changes. One tacit assumption, as in recent work on critical phase transitions and "early warning signs" [8] is that the physical process of state transition (here, the cell fate switch) is driven by a gradual, monotonical change of the bifurcation parameter *P* in "real time" [2, 9].

Our data matrix $\boldsymbol{X}(t)$ represents the ensemble of individual dynamical systems each of which is a row vector in the data matrix (a cell). We ask how aggregate quantities of *X*, namely the coefficients for row-by-row and column-by-column correlation that is here of interest, change as the system *X* undergoes a bifurcation-driven state transition as one bifurcation parameter *P* changes.

Since each row is a statistical replicate of the same deterministic dynamical system, we first collapse $\boldsymbol{X}$ to a single state vector representing a dynamical system $\boldsymbol{X}(t)$ in m dimensions (m genes) and discretize time to obtain the following difference equation for an m-dimensional dynamical system:

$$\boldsymbol{X}(t+1) = \boldsymbol{F}(\boldsymbol{X}(t); \boldsymbol{P}(\tau)) \,. \qquad (4)$$

The state of a cell at time t is represented by $\boldsymbol{X}(t)$ which is now a state *vector*, $\boldsymbol{X}(t) = (x_1\ (t), x_2\ (t), \cdots, x_m(t))$. $\boldsymbol{P}(\tau)$ is a parameter vector $\boldsymbol{P}(\tau) = (p_1(\tau), p_2(\tau), \cdots, p_s(\tau))$ which represents the factors which monotonically change with time τ and drive the dynamical system from an attractor state $\boldsymbol{X}^*$ to an unstable state through a bifurcation. ($\boldsymbol{X}^*$ is the stable state of untreated cells prior to placing them in differentiating condition upon which *P* begins to change.)

Recently Chen, Liu and colleagues [4, 10] have solved a similar problem for microarray data in which naturally only the ensemble average of the system state is available and thus, the stochastic exploration of the state space is achieved by the use of measurement replicates (e.g. in distinct samples or in repeats in time). Another difference is that in Liu et al. (2012) [10], because of use of microarrays, the dimensionality *m* is immense (genome-scale) and it was assumed that a subset of genes x_i do not contribute to the dynamics. Hence genes were partitioned into two groups, only a subset of which form a (sub)network that drives the state change. Here all genes are expected to participate in the state transition because they were selected for it. Therefore, the *a priori* distinction is not made here and all genes are expected to contribute to some (however small) degree to the dynamics.

We assume that $\boldsymbol{X}^*(\boldsymbol{P})$ (later we will drop $\boldsymbol{P}$ for simplicity) is a hyperbolic fixed-point attractor, thus, $\boldsymbol{X}^* = \boldsymbol{F}(\boldsymbol{X}^*(\boldsymbol{P}); \boldsymbol{P})$. We further assume, in loose analogy to the quasi-equilibrium approach in chemical thermodynamics, that during fate decision as $\boldsymbol{P}$ changes in real time, the system $\boldsymbol{X}(t)$ stays in the hyperbolic attractor. Thus, according to the Hartman-Grobman theorem the dynamics of the original system can, at any time during our differentiation process (up to the bifurcation), be approximated by the linearized system near the fixed point attractor [11]. (We do not consider more complex attractors, although it has been suggested, notably in conjunction of cell diversification, that cell types could be represented by strange attractors [12].)

To consider the stochastic exploration of the state space near the curve of $\boldsymbol{X}^*(\boldsymbol{P})$ we define $\Delta\boldsymbol{X} = \boldsymbol{X} - \boldsymbol{X}^*$ then substitute $\boldsymbol{X} = \Delta\boldsymbol{X} + \boldsymbol{X}^*$ into Eq. (4) [10]:

$$\begin{aligned}\Delta\boldsymbol{X}(\boldsymbol{t}+\mathbf{1}) \quad + \boldsymbol{X}^* &= \boldsymbol{F}(\boldsymbol{X}^* + \Delta\boldsymbol{X}(\boldsymbol{t})) \\ &= \boldsymbol{F}(\boldsymbol{X}^*) + \boldsymbol{J}\Delta\boldsymbol{X}(t) + \boldsymbol{O}\left(\Delta\boldsymbol{X}^{\mathbf{2}}\right)\end{aligned}$$

with the Jacobian matrix $\boldsymbol{J} = \left.\frac{\partial \boldsymbol{F}(\boldsymbol{X};\boldsymbol{P})}{\partial \boldsymbol{X}}\right|_{\boldsymbol{X}=\boldsymbol{X}^*}$ evaluated at the attractor state $\boldsymbol{X}^*$. Neglecting the higher order terms $\boldsymbol{O}(\Delta\boldsymbol{X}^{\mathbf{2}})$ and with $\boldsymbol{F}(\boldsymbol{X}^*) = \boldsymbol{X}^*$ for the quasi-equilibrium condition we have

$$\Delta\boldsymbol{X}(\boldsymbol{t}+\mathbf{1}) \quad = \boldsymbol{J}\Delta\boldsymbol{X}(\boldsymbol{t}) \tag{6}$$

Since $\boldsymbol{X}^*$ is a hyperbolic fixed-point attractor up to the bifurcation, there must exist a critical $\boldsymbol{P_c}$ for this discrete dynamical system such that one of the eigenvalues of Jacobian matrix $\boldsymbol{J} = \left.\frac{\partial \boldsymbol{F}(\boldsymbol{X};\boldsymbol{P})}{\partial \boldsymbol{X}}\right|_{\boldsymbol{X}=\boldsymbol{X}^*}$ equals 1 while for $\boldsymbol{P} \neq \boldsymbol{P_c}$, no eigenvalues of Jacobian matrix equals 1.

We now need to expand our state vector back to the gene expression data matrix $\boldsymbol{X}(t)$ (*n* rows = *n* cells, *m* columns = *m* genes) as in (1), representing the gene expression values x of a cell ensemble in a given condition at a given time t. Since we deal with a statistical ensemble we convert the deterministic system to a stochastic system by adding the random perturbation $\boldsymbol{\zeta}(t)$ to each cell state variable. With this framework we now first consider the gene-gene correlation and subsequently the cell-cell correlation.

(a) Gene-gene correlation $\langle \boldsymbol{R}(\boldsymbol{g_i}, \boldsymbol{g_j})\rangle$

Since Jacobian matrix $\boldsymbol{J}$ is of full rank, there exists a full-rank normal matrix S satisfying $\boldsymbol{J} = \boldsymbol{S\Lambda S^{-1}}$, where $\boldsymbol{\Lambda}$ is a diagonal matrix with m eigenvalues as the entries. We define $\boldsymbol{Y}(\boldsymbol{t}) = \boldsymbol{S^{-1}}\Delta\boldsymbol{X}(\boldsymbol{t})$ to express the data matrix in terms of eigenvalues. With Eq. (6) and introducing here the random perturbation $\boldsymbol{\zeta}(t)$ we obtain:

$$\boldsymbol{Y}(t+1) = \boldsymbol{\Lambda Y}(t) + \boldsymbol{\zeta}(t) \tag{7}$$

where $\boldsymbol{Y}(t) = (y_1\ (t), y_2\ (t), \cdots, y_m(t))$. $\boldsymbol{\zeta}(t)$ represents Gaussian noise with zero mean and covariance $Cov(\zeta_i, \zeta_j) = \kappa_{ij}$. Since $\Delta\boldsymbol{X}$ are small symmetric perturbations of gene expression values, expectation $\boldsymbol{E}(\Delta\boldsymbol{X}) = 0$ and $\boldsymbol{E}(\boldsymbol{Y}) = 0$.

With the stationarity condition, we calculate the variance of the ith component of $\boldsymbol{Y}(t)$:

$$\begin{aligned}Var(y_i) &= Var(y_i(t+1)) \\ &= \boldsymbol{E}\left(y_i^2(t+1)\right) - \boldsymbol{E}\left(y_i\ \ (t+1)\right)^2 = \boldsymbol{E}\left([\lambda_i y_i(t) + \zeta_{\boldsymbol{i}}(\boldsymbol{t})]^{\mathbf{2}}\right) \\ &= \lambda_i^{\mathbf{2}}\boldsymbol{E}\left(y_i^2(t)\right) + \kappa_{ii} = \lambda_i^{\mathbf{2}} Var(y_i) + \kappa_{ii}\end{aligned} \tag{8}$$

Since $\boldsymbol{E}\left(y_i^2(t)\right) \neq 0$ and $\kappa_{ii} > 0$

$$Var(y_i) = \frac{\kappa_{ii}}{1-\lambda_i^2} \tag{9}$$

When $\lambda_1 \to 1$ (bifurcation in discrete dynamics) and κ_{ii} holding constant, $Var(y_i) \to \infty$. Thus, when a dynamical system approaches the critical transition, the variances of genes related to $\lambda_1 \to 1$ will significantly increase. As the other eigenvalues $\lambda_i (i = 2,3,\cdots m)$ satisfy$0 \leq |\lambda_i| < 1$, the variances of other genes approach positive finite values.

The covariance of y_i and y_j is:

$$Cov(y_i, y_j) = \boldsymbol{E}(y_i y_j) - \boldsymbol{E}(y_i)\boldsymbol{E}(y_j) = \boldsymbol{E}(y_i y_j). \tag{10}$$

Using the diagonalised matrix $\boldsymbol{\Lambda}$ with the eigenvalues we have

$$\begin{aligned} Cov(y_i, y_j) &= Cov(y_i(t+1), y_j(t+1)) \\ &= \boldsymbol{E}\left(y_i\ (t+1) y_j\ (t+1)\right) = \boldsymbol{E}\left((\lambda_i y_i(t) + \zeta_{\boldsymbol{i}}(\boldsymbol{t}))\ \ (\lambda_j y_j(t) + \zeta_{\boldsymbol{j}}(\boldsymbol{t}))\ \right) \\ &= \lambda_i\ \lambda_j\ \boldsymbol{E}(y_i\ \ y_j) + \kappa_{ij} = \lambda_i\ \lambda_j\ Cov(y_i, y_j) + \kappa_{ij} \end{aligned}$$

Since $\boldsymbol{E}(y_i\ \ , y_j) \neq 0$ and $\kappa_{ij} > 0$

$$Cov(y_i, y_j) = \frac{\kappa_{ij}}{1-\lambda_i\lambda_j} \tag{11}$$

For calculating the Pearson correlation coefficient R between the original gene expression vectors $\boldsymbol{x}(t)$ (columns in $\boldsymbol{X}(t)$)

$$R(x_i, x_j) = \frac{Cov(x_i, x_j)}{\sqrt{Var(x_i)Var(x_j)}} \tag{12}$$

we use the transformation $\boldsymbol{X} - \boldsymbol{X}^* = \Delta\boldsymbol{X} = \boldsymbol{SY}$ from above:

$$\begin{aligned} \boldsymbol{x}_i &= s_{i1}y_1 + \cdots + s_{im}y_m + \boldsymbol{x}_i^* \\ \boldsymbol{x}_j &= s_{j1}y_1 + \cdots + s_{jm}y_m + \boldsymbol{x}_j^* \end{aligned} \tag{13}$$

Here we calculate the variance of original gene expression $\boldsymbol{X}$ column-wise:

$$Var(\boldsymbol{x}_i) = \sum_{i=1}^{m} s_{i1}^2 Var(y_i) + \sum_{k,l=1,k\neq l}^{m} s_{ik}s_{il}\, Cov(y_k, y_l) \tag{14}$$

$$\begin{aligned} Cov(\boldsymbol{x}_i, \boldsymbol{x}_j) &= \boldsymbol{E}\left((s_{i1}y_1 + \cdots + s_{im}y_m)(s_{j1}y_1 + \cdots + s_{jm}y_m)\right) \\ &= s_{i1}s_{j1}Var(y_1) + \cdots + s_{im}s_{jm}Var(y_m) \\ &\quad + \sum_{k,l=1,k\neq l}^{m} s_{ik}s_{il}\, Cov(y_k, y_l) \end{aligned} \tag{15}$$

$$R(\boldsymbol{x}_i, \boldsymbol{x}_j) \quad = \quad \frac{Cov(x_i, x_j)}{\sqrt{Var(x_i)Var(x_j)}}$$

$$= \frac{s_{i1}s_{j1}\frac{\kappa_{11}}{1-\lambda_1^2} + \cdots + s_{im}s_{jm}\frac{\kappa_{mm}}{1-\lambda_m^2} + \sum_{k,l=1,k\neq l}^{m} s_{ik}s_{il}\,\frac{\kappa_{kl}}{1-\lambda_k\lambda_{jl}}}{\sqrt{s_{i1}^2\frac{\kappa_{11}}{1-\lambda_1^2} + \cdots + s_{im}^2\frac{\kappa_{mm}}{1-\lambda_m^2} + \sum_{k,l=1,k\neq l}^{m} s_{ik}s_{il}\,\frac{\kappa_{kl}}{1-\lambda_k\lambda_{jl}}}} * \frac{1}{\sqrt{s_{j1}^2\frac{\kappa_{11}}{1-\lambda_1^2} + \cdots + s_{jm}^2\frac{\kappa_{mm}}{1-\lambda_m^2} + \sum_{k,l=1,k\neq l}^{m} s_{jk}s_{jl}\,\frac{\kappa_{kl}}{1-\lambda_k\lambda_{jl}}}}$$

for $\lambda_1 \to 1$, the term $\frac{\kappa_{11}}{1-\lambda_1^2}$ becomes the dominating term, and all other terms $s_{im}s_{jm}\frac{\kappa_{mm}}{1-\lambda_m^2}$, $\sum_{k,l=1,k\neq l}^{m} s_{ik}s_{il}\,\frac{\kappa_{kl}}{1-\lambda_k\lambda_{jl}}$ can be neglected. For the gene pair (x_i, x_j), if s_{i1}, s_{j1} are not zeros, the Pearson correlation coefficient for gene-gene correlation is:

$$R(x_i, x_j) \to \frac{s_{i1}s_{j1}\frac{\kappa_{11}}{1-\lambda_1^2}}{\sqrt{s_{i1}^2\frac{\kappa_{11}}{1-\lambda_1^2}}\sqrt{s_{j1}^2\frac{\kappa_{11}}{1-\lambda_1^2}}} \to 1 \;\; or -1 \qquad (17)$$

Note that if either s_{i1} or s_{j1} is zero, i.e. a gene does not change along λ_1 (the "reaction coordinate"), then $(\boldsymbol{x}_i, \boldsymbol{x}_j) = 0$.

(b) Cell-cell correlation $\langle \boldsymbol{R}(\boldsymbol{S}^k, \boldsymbol{S}^l)\rangle$

Using the same gene expression data matrix (*n* rows – *n* cells, *m* columns – *m* genes) representing the gene expressions of a cell ensemble in a given condition at a given time t,

$$\boldsymbol{X}(t) = \begin{bmatrix} x_1^1 & \cdots & x_m^1 \\ \vdots & \ddots & \vdots \\ x_1^n & \dots & x_m^n \end{bmatrix}$$

We now calculate the "transpose" quantity for our index I_C, the row-wise Pearson correlation of gene expression value vectors of individual cells $\boldsymbol{S}^k$, $\boldsymbol{S}^l$ $\dots$ or $\boldsymbol{x}^k, \boldsymbol{x}^l$.. in terms of matrix $\boldsymbol{X}(t)$:

$$R(\boldsymbol{x}^k, \boldsymbol{x}^l) = \frac{Cov(\boldsymbol{x}^k, \boldsymbol{x}^l)}{\sqrt{Var(\boldsymbol{x}^k)Var(\boldsymbol{x}^l)}} \qquad (18)$$

Through the transformation $\boldsymbol{x}^{\boldsymbol{k}} - \boldsymbol{x}^{\boldsymbol{k}^*} = \boldsymbol{z}^{\boldsymbol{k}}$, i.e. we move the hyperbolic fixed point attractor to the coordinate origin $\boldsymbol{O}$. $\boldsymbol{Z}^{\boldsymbol{k}}$ are small independent perturbations on m genes in k^{th} cell: $\boldsymbol{E}(\boldsymbol{z}^k) = 0, (\mathrm{k} = 1, \cdots, \mathrm{n})$

$$\begin{aligned} \boldsymbol{x}^k &= (z_1^k, \cdots, z_m^k) \qquad + \boldsymbol{x}^{k*} \\ \boldsymbol{x}^l &= (z_1^l, \cdots, z_m^l) \qquad + \boldsymbol{x}^{l*} \end{aligned} \qquad (19)$$

The variances of the row vector – m gene expressions of kth cell $\boldsymbol{x}^k = (x_1^k, \cdots, x_m^k)$ and m gene expressions of lth $\boldsymbol{x}^l = (x_1^l, \cdots, x_m^l)$ are:

$$\begin{aligned} Var(\boldsymbol{x}^k) &= \boldsymbol{E}\left(\left(\boldsymbol{z}^k\right)^2\right) - \left(\boldsymbol{E}(\boldsymbol{z}^k)\right)^{\mathbf{2}} \\ &= \frac{1}{m}((z_1^k)^2 + \cdots + (z_m^k)^2) - \left(\boldsymbol{E}(\boldsymbol{z}^k)\right)^{\mathbf{2}} \\ &= \frac{1}{m}((z_1^k)^2 + \cdots + (z_m^k)^2) \end{aligned} \tag{20}$$

$$\begin{aligned} Var(\boldsymbol{x}^l) &= \boldsymbol{E}\left(\left(\boldsymbol{z}^l\right)^2\right) - \left(\boldsymbol{E}(\boldsymbol{z}^l)\right)^{\mathbf{2}} \\ &= \frac{1}{m}((z_1^l)^2 + \cdots + (z_m^l)^2) - \left(\boldsymbol{E}(\boldsymbol{z}^l)\right)^{\mathbf{2}} \\ &= \frac{1}{m}((z_1^l)^2 + \cdots + (z_m^l)^2) \end{aligned} \tag{21}$$

If $(z_i^1, \cdots, z_i^k, \cdots, z_i^l, \cdots, z_i^n)$ contributes to the eigenevector y_1

$$Var(y_1) = \frac{\kappa_{11}}{1-\lambda_1^2} \tag{22}$$

for $\lambda_1 \to 1$ and κ_{ii} holding constant, $\frac{\kappa_{11}}{1-\lambda_1^2}$ becomes ∞, $(z_i^k)^2$ and $(z_i^l)^2$ will significantly increase. Therefore $Var(\boldsymbol{x}^k) \cdot Var(\boldsymbol{x}^l)$ will also significantly increase.

The covariance of row vector $\boldsymbol{x}^k$ and $\boldsymbol{x}^l$

$$\begin{aligned} Cov(\boldsymbol{x}^k, \boldsymbol{x}^l) &= \boldsymbol{E}(\boldsymbol{z}^k\, \boldsymbol{z}^l) - \boldsymbol{E}(\boldsymbol{z}^l)\boldsymbol{E}(\boldsymbol{z}^k) \\ &= \boldsymbol{E}(\boldsymbol{z}^k\, \boldsymbol{z}^l) \\ &= \frac{1}{m}(z_1^k z_1^l + \cdots + z_m^k z_m^l) \end{aligned} \tag{23}$$

Even $(z_i^1, \cdots, z_i^k, \cdots, z_i^l, \cdots, z_i^n)$ contributes to the eigenevectory_1, $z_i^k z_i^l$ does not necessarily increase. If $(z_i^1, \cdots, z_i^k, \cdots, z_i^l, \cdots, z_i^n)$ relate to other $\lambda_i (i = 2,3, \cdots m)$ which satisfies $0 \le |\lambda_i| < 1$, the covariance of row vector $\boldsymbol{x}^k$ and $\boldsymbol{x}^l$ will approach finite values.

Therefore, the row-wise Pearson correlation coefficients of cells' gene expression $PCC(\boldsymbol{x}^k, \boldsymbol{x}^l)$ decrease towards the critical transition point as cell states diversify:*

$$R(\boldsymbol{x}^k, \boldsymbol{x}^l) = \frac{Cov(\boldsymbol{x}^k, \boldsymbol{x}^l)}{\sqrt{Var(\boldsymbol{x}^k)Var(\boldsymbol{x}^l)}} \to \frac{\text{not necessarily increase}}{significant\ increase} \to decrease \tag{24}$$

----

* Using *average of correlation coefficients* between cell state vectors, as opposed to simply taking the multivariate *variance* of the cell expression vectors, to capture cell diversity and its change, will also reflect a change in a bifurcation parameter if the position of the attractor state also changes with the bifurcation parameter. Here the difference between the two methods was not relevant.

References for S2 Appendix (B)

1. Zhou JX, Aliyu MDS, Aurell E, Huang S. Quasi - potential landscape in complex multi - stable systems. J. R. Soc. Interface. 2015; 12:1-15.
2. Huang S, Guo YP, May G, Enver T. Bifurcation dynamics in lineage-commitment in bipotent progenitor cells. Dev. Biol. 2007; 305:695–713.
3. Ye H, Beamish RJ, Glaser SM, Grant SCH, Hsieh CH, Richards LJ, et al. Equation-free mechanistic ecosystem forecasting using empirical dynamic modeling. Proc. Natl. Acad. Sci. 2015; 112:E1569–1576.
4. Chen L, Liu R, Liu ZP, Li M, Aihara K. Detecting early-warning signals for sudden deterioration of complex diseases by dynamical network biomarkers. Sci. Rep. 2012 2:18–20.
5. Scheffer M, Bascompte J, Brock WA, Brovkin V, Carpenter SR, Dakos V, et al. Early-warning signals for critical transitions. Nature. 2009; 461:53–9.
6. Guilford JP. Fundamental Statistics in Psychology and Education. McGraw-Hill, New York; 1965.
7. Wang J, Xu L, Wang E, Huang S. The potential landscape of genetic circuits imposes the arrow of time in stem cell differentiation. Biophys. J. 2010; 99:29–39.
8. Scheffer M, Carpenter SR, Lenton TM, Bascompte J, Brock W, Dakos V, et al., Anticipating critical transitions. Science. 2012;338:344–8.
9. Zhou JX, Brusch L, Huang S. Predicting pancreas cell fate decisions and reprogramming with a hierarchical multi-attractor model. PLoS One. 2011; 6:e14752.
10. Liu R, Li M, Liu ZP, Wu J, Chen L, Aihara K. Identifying critical transitions and their leading biomolecular networks in complex diseases. Sci. Rep. 2012; 2:813.
11. Perko L. Texts in Applied Mathematics. Vol. Book 7. eds J.E. Marsden, L. Sirovich, & M. Golubisky; 557; Springer New York; 2006.
12. Furusawa, C. and K. Kaneko (2012). "A dynamical-systems view of stem cell biology." Science 338 (6104): 215-217.
