# Supplementary material for: Cell Fate Decision as High-Dimensional Critical State Transition
Source: PLoS Biol. 2016 Dec 27;14(12):e2000640. doi: 10.1371/journal.pbio.2000640 (PMC5189937; doi:10.1371/journal.pbio.2000640)
Supplement: S3 Appendix — (PDF) [file pbio.2000640.s017.pdf]

# S3 Appendix

## C. Additional support from analysis of public data

*As discussed in the main text, in this Appendix we present the details of the analysis of two published data sets for additional support of robustness (see also A.8.) and validity of the index $I_C$. We show how the index $I_C$ is statistically robust: (**1**) $I_C$ does not fluctuate randomly, making it unlikely that the observed increase of $I_C$ before the critical transition is due to a chance event; (**2**) using a small number of genes (e.g., m=20 genes instead of the entire transcriptome) to compute $I_C$ yields robust results. The first example of lung cell differentiation also displays the increase of $I_C$ before the fate commitment for an epithelial cell.*

### C.1. Lung epithelium development (from Treutlein et al. [1])

In this example from [1] the transition of lung-epithelium progenitor cells into one of two differentiated cell types, here AT2, is examined. Four time points (embryonic days, E) with sufficient data are available for our analysis: E14.5, E16.5 (=around the critical transition into AT2), 18.5 (transitioned to AT2) and adult (see scheme **Fig C-1a**). We re-analyzed their scRNA-Seq data (with the caveats explained in Section A.7 of this Supplement) in new ways: (*i*) to confirm that $I_C$ predicts the impending critical transition (fate commitment to the AT2 lineage) after E16.5 and (*ii*) to demonstrate that the number of genes used does not affect the outcome. After processing the raw data as described in [1], we continued our analysis with ~15.000 genes for 163 cells (sum across all time points) (see **Fig C-1a**). First, we calculated $I_c$ for each time point using all ~15,079 genes (not shown). To demonstrate that the number of genes does not affect the expected pattern (increase of $I_c$ before the bifurcation after E16.5) we used 20, 200 or 2000 genes selected by random subsampling from the total of ~ 15,000 genes to compute $I_C$. In all cases the average value of $I_C$ significantly increased from E14.5 to E16.5 –just before the fate branch point which marks the critical transition (** in **Fig C-1b**, **c**). [Note that the subsequent decrease of $I_C$ after the transition is not guaranteed by the mathematical derivation of $I_C$ but plausible because it reflects the widely held idea that differentiated cells are in deeper attractors]. Thus, in this *in vivo* example, $I_C$ behaves as predicted by theory.

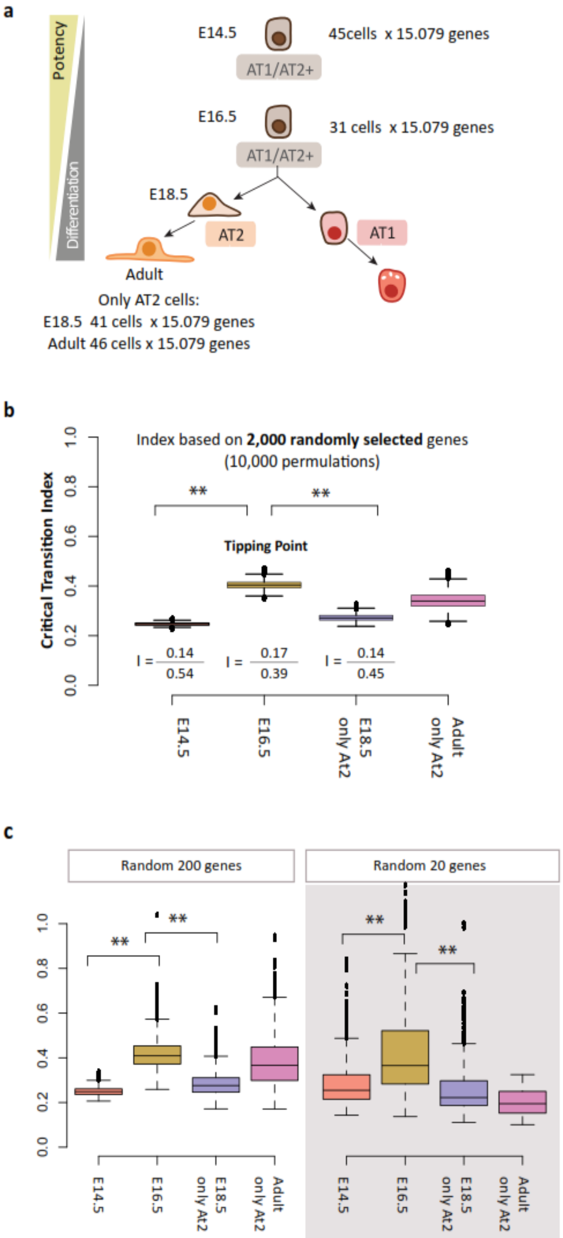


**Figure C-1**. Increase of critical transition index $I_C$ for alveolar cell differentiation. ***a.*** The bifurcation event takes place around E16.5. Scheme from [20]. $I_C$ values computed from single-cell RNA-Seq measurement of whole transcriptomes, snapshots at the indicated 4 time points. $I_C$ (y-axis) was computed from 2000 (***b***), 200 and 20 (***c***) randomly selected genes in 10,000 permutations and results displayed as box plots (box indicates 25th to 75th percentile, whiskers indicate the upper and lower adjacent values, and dots are outliers). **indicate $p$-value < 2E-10 for comparison between time points (nonparametric tests, see text).

Recall that from how $I_C$ is computed it is very difficult for it to produce a change of the magnitude observed just by chance, as the elementary *p*-value calculations in Section **A.8**. and in example **C.1.** show. Reducing the number of genes from ~15000 to 2000, 200 and 20 for calculating index $I_C$ preserves the predicted relative change of $I_C$ before E18.5 despite random selection of genes.

Note that, as expected, the variance in $I_C$ is substantially higher when fewer genes are used to compute $I_C$ (**Fig C-1c**) but the change in $I_C$ is still statistically significant (Kolmogorov-Smirnov test, $p$<2e-16; Mann-Whitney test, $p$<2.2E-10). Also, remember that in scRNA-seq the vast majority of transcripts of the transcriptome is not detected in any given cell or detected in only a subfraction of cells (as discussed in Section **A.7**). Data were filtered for genes with FPKM>0.5 in at least 5% of cells. For this reason, random selection from the entire transcriptome of a small subset of genes can produce noise, as seen in this example. **Fig. C2** shows the numerator and denominator for computing $I_C$ in Figs C-1, for *all* genes, revealing the expected trend.

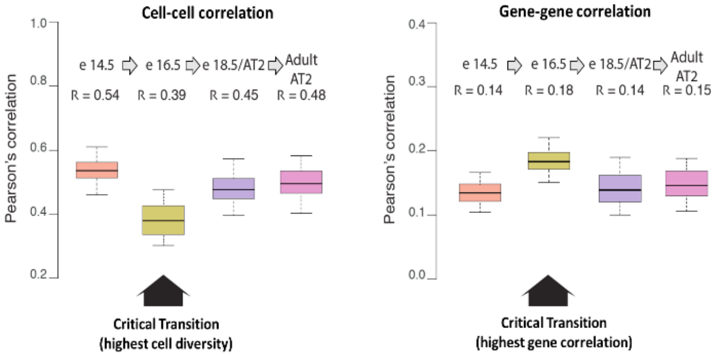


**Figure C-2**. The average cell-cell and gene-gene correlation coefficients *R* that underlie the index $I_C$ in Fig C-1. Mean and distribution of the average cell-cell correlation (LEFT) and of the average gene-gene correlation (RIGHT) as in Fig. C-1; but for all ~15,000 genes. Boxplots calculated as in Fig. 1C.

By contrast, in the main text only the genes that are expressed and known to be involved in the transition are used. The fact that we used *specifically* selected genes to demonstrate the increase of $I_C$ is a strength of the approach, not a weakness: The goal is precisely to examine a predicted concerted change of expression levels of the set of genes $x_i$ that are known to be *members* of the dynamical system $F(\mathbf{x})$ with $\mathbf{x}=\{x_i\}$ –the core idea that underlies the derivation of $I_C$. The fact that randomly selected genes also, albeit to a lesser extent, display the predicted behavior in $I_C$ suggests a possible generalizability. It is however plausible given the well-known overall correlated expression behavior of all genes across the gnome, manifesting the fact that the underlying gene regulatory network acts as an integrated dynamical system. One thus expects that using genes of the core network to compute $I_C$ as in the main text will perform much better than randomly chosen genes, which in fact was the case: The increase of $I_C$ observed for 17 selected genes in the main text was far more pronounced (>2-fold, Fig 2B in main text) and significant than the case of 20 random genes shown here (**Fig C-1c**).

## C.2. Glioblastoma multiforme (GBM) cells (from Patel et al. [2])

To analyse the dependence of $I_C$ from the gene number in more detail, we next used scRNA-Seq data from static tumor samples to challenge the robustness of $I_C$ by considering a case with very heterogeneous cells (inherently low cell-to-cell correlation). Thus, we computed the $I_C$ values for four cell populations of samples of primary tumor cells as well and cell lines of glioblastoma multiforme (GBM) –a tumor known for its extreme cellular heterogeneity– using the data from Patel et al. [2]. The provenience of the sample from such a heterogeneous tumor causes the departure from the usual relative homogeneity of cells in non-tumor cell lines that creates a global correlation between cells. The data are from a static measurement of patient samples, not from the

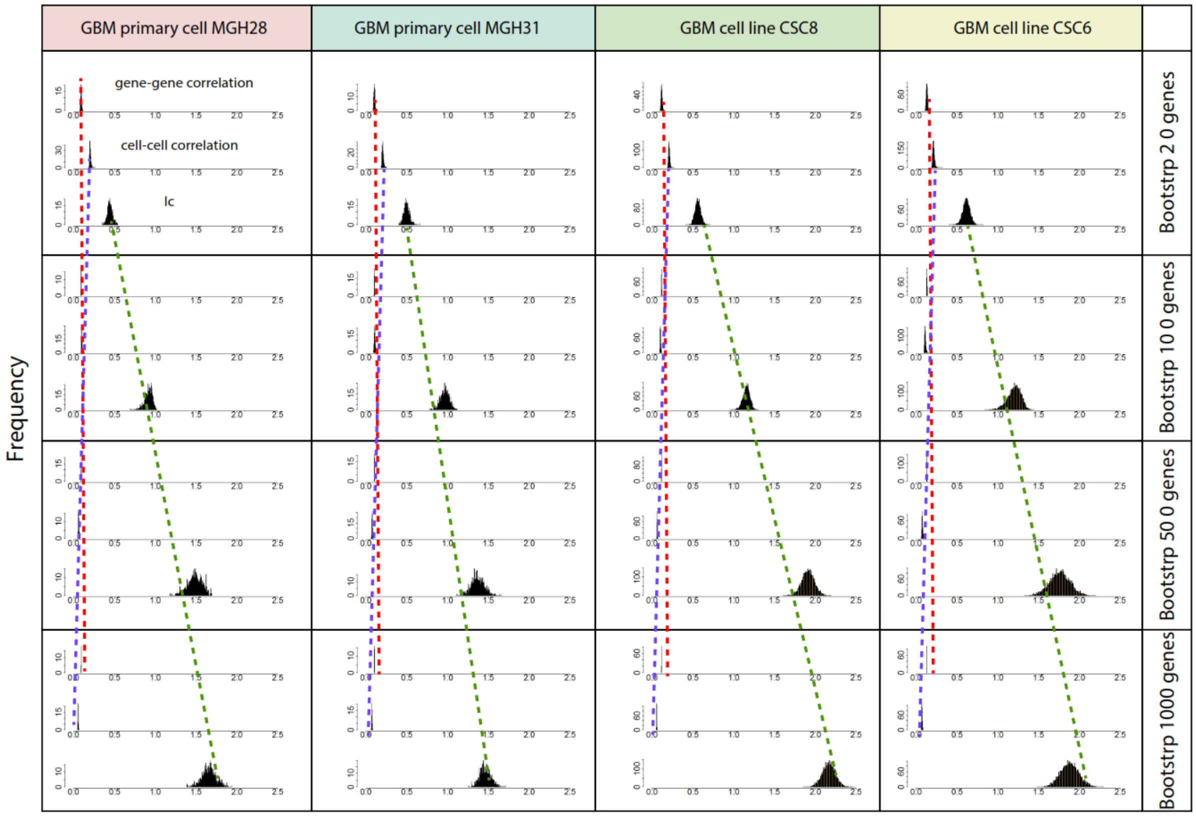


**Figure C-3.** Dissection of the $I_C$ value for static cell populations of gliablastoma multiforme cells. Each column represents primary GBM tumor-derived cells or cells from established GBM line. Each subplots row represents the number of genes used to compute the gene-gene correlations (top row histogram of each subplot), cell-cell correlations (middle row) and their ratio, the index $I_C$ (bottom row). The histograms represent the distribution of the results obtained from 1000 runs of sampling of genes for each bootstrap analysis. Dashed lines serve as optical guides to show that with increasing number of genes used because of the heterogeneity of cells: the cell-cell correlation (blue) (the denominator of $I_C$) decreases more drastically than gene-gene correlation (red) –resulting in an increase of the index $I_C$ *(green)* Note that the spread of $I_C$ at 20 genes, even in these heterogeneous cells, is only 0.5±0.2 and cannot explain a doubling of $I_C$ as a chance event ($p$<<0.01).

study of a dynamical process, and hence, we do not evaluate the prediction of critical transitions by $I_C$. We computed $I_C$ for four subpopulations of GBM cells (columns in **Fig C-3**), each consisting of around $N$=80 cells on average (total 430 cells), in order to evaluate the robustness of $I_C$ as a function of number of genes used in the presence of inherently heterogeneous cell populations.

We used randomly chosen sets of $m$=20, 100, 500 and 1000 genes from the transcriptome as input for computing the cell-cell, gene-gene correlation averages (numerator and denominator of $I_C$) and $I_C$; we repeated the calculation 1000 times. **Fig C-3** shows the average cell-cell correlation (top rows in each subplot), gene-gene correlation (middle rows) and their ratio = $I_C$ (bottom row) as histograms of distributions of the values obtained for the 1000 runs. If only 20 genes were used to compute $I_C$, the spread of $I_C$ was around ~0.25 and it increased to ~0.5 as the gene number used increased to $m$=1000 genes. There was also an increase in the absolute value of $I_C$ when more genes were included –a trend not seen in the previous example for normal lung cells. Comparison of the subplots (see guiding dashed lines in **Fig 3-C**) show that this increase in $I_C$ for larger gene numbers is caused by the supra-proportional decrease of cell-cell correlation (blue dashed line). This trend is opposite to the standard case explained in **A.8**. where there is substantial correlation among cells and reflects the extreme *heterogeneity* of the GBM cells such that inclusion of more genes *accentuates* the measure of their diversity.

This is an important demonstration of the reliability of the proposed index: when there is no strong cell-cell correlation (extreme high heterogeneity of GBM cells), increasing the number of genes decreases the apparent average cell-cell correlation because of the increased "sampling efficiency" of the system – the opposite of the range effect (as discussed in **A.8** and **B.2**). That is to say that the cell-cell correlation cannot be reached by the pure 'brute force' of increasing the range (using more genes to compute the correlation). Note that the range effect only holds when some correlation is already there, in which case increasing the expression range considered will permit to overcome noise. Otherwise, as in the case of GBM with dominating biological cell-cell variability (no common deep attractor) correlation cannot emerge by chance when increasing gene numbers.

The absolute value of $I_C$ which is inflated when here is low baseline cell-cell correlation, however, is not relevant for the detection of critical transitions. What matters is its *increase* of $I_C$ between time points (not considered in t is static example). A key result here is that the fluctuation of $I_C$ due to the random sampling of genes in the bootstrap analysis was minimal: Considering the observed distributions, the *p*-value for a change of $I_C$ by 2-fold (the magnitude that we have observed in our data in the manuscript) is $p < 0.01$ for all cases of gene numbers used. (*p*-value was calculated from the $I_c$ density distribution estimated from 1000 random samples). Thus, even a highly heterogeneous population of cells and randomly selecting 20 genes gave rise to fluctuations in $I_C$ that are far too low to explain that the change of $I_C$ by 2-fold occurred by chance –as bootstrap analysis in our manuscript (error bars in Fig 2B of main text) already suggested. In more general terms, the underlying reason for the robustness of $I_C$ lies in the averaging of a large number of correlation coefficients between high-dimensional vectors in both directions of the same data matrix.

Finally, we note that most data in the past literature, as in the two examples shown here, typically contain 50-100 (or less) cells in total which precludes a boot-strap analysis in the dimension of cells. By contrast, in the main text $I_C$ was computed from 150 cells at each time point. Moreover, the gene expression profiles for subpopulations were verified by microarrays.

**References for S3 Appendix (C)**

1. Treutlein B, Brownfield DG, Wu AR, Neff NF, Mantalas GL, Espinoza FH, et al. Reconstructing lineage hierarchies of the distal lung epithelium using single-cell RNA-seq. Nature. 2014; 509(7500):371-375.
2. Patel AP, Tirosh I, Trombetta JJ, Shalek AK, Gillespie SM, Wakimoto H, et al. Single-cell RNA-seq highlights intratumoral heterogeneity in primary glioblastoma. Science. 2014; 344(6190):1396-1401
